# Synthesis and Catalytic Properties of a Very Latent Selenium-Chelated Ruthenium Benzylidene Olefin Metathesis Catalyst

**DOI:** 10.1021/acs.organomet.1c00484

**Published:** 2021-10-25

**Authors:** Louis Monsigny, Joel Cejas Sánchez, Jakub Piątkowski, Anna Kajetanowicz, Karol Grela

**Affiliations:** Biological and Chemical Research Centre, Faculty of Chemistry, University of Warsaw, Żwirki i Wigury 101, 02-089 Warsaw, Poland

## Abstract

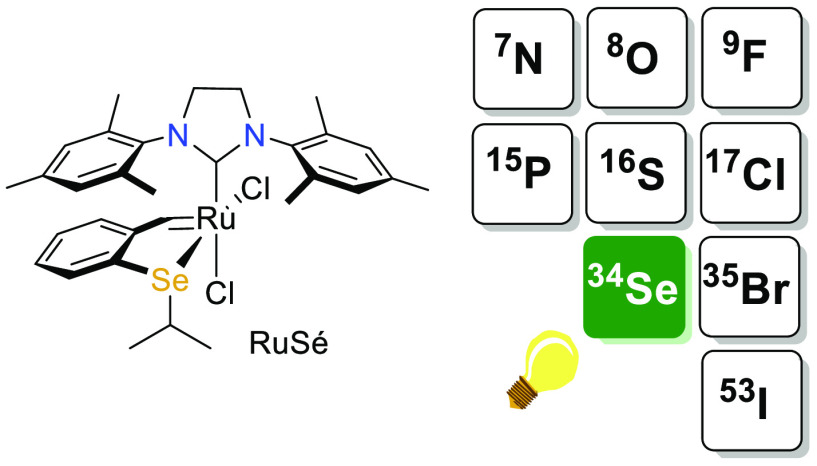

Herein, we describe a study of the synthesis, characterization, and catalytic properties of a *cis*-dichlorido seleno-chelated Hoveyda–Grubbs type complex (**Ru8**). Such a complex has been obtained through a straightforward and high-yielding synthetic protocol in three steps from the commercially available 2-bromobenzaldehyde in good overall yield (54%). The catalytic profile, especially the latency of this complex, has been probed through selected olefin metathesis reactions such as ring-closing metathesis (RCM), self-cross-metathesis (self-CM) and ring-opening metathesis polymerization (ROMP). In addition to its high latency, the selenium Hoveyda-type complex **Ru8** exhibits a switchable behavior upon thermal activation. Of interest, while the corresponding sulfur-chelated Hoveyda type catalyst is reported to be only activated by heat, the selenium analogue was found to be active upon both heat and light irradiation.

## Introduction

**Figure 1:**
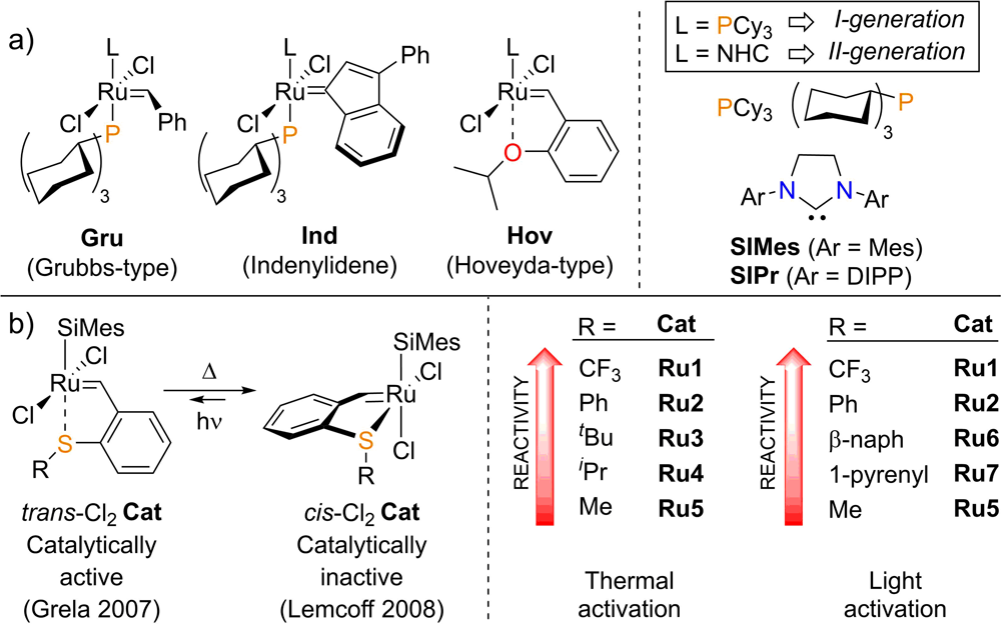
(a) Selected ruthenium-based catalysts for olefin metathesis and the neutral ligands. (b) *trans*- and *cis*-dichlorido *S*-chelated Ru benzylidene complexes and the influence of the *S* substituent on the reactivity. (Mes = mesityl (2,4,6-trimethylphenyl), DIPP = 2,6-diisopropylphenyl, NHC = *N*-heterocyclic carbene, SIMes =1,3-bis-mesityl-2-imidazolidin-2-ylidene, SIPr = 1,3-bis-(2,6-triisopropylphenyl)-2-imidazolidinylidene).

Since carbon–carbon bond formation is a central aim in organic chemistry, olefin metathesis takes a crucial place in this field. The versatility and the wide application scope of metathesis allowed its use for compounds relevant as pharmaceuticals and the preparation of well-defined polymers as well as challenging target-oriented synthesis, on both the academic and industrial level.^1^ This methodology became widespread along with the successive enhancement of the catalyst of olefin metathesis, starting from the first well-defined ruthenium based Grubbs complex (PCy_3_)_2_(Cl)_2_Ru=CHPh (**Gru-I**, Figure 1)^2^ followed by the introduction of an *N*-heterocylic ligand (NHC) yielding to the second-generation Grubbs type catalyst (NHC)(PCy_3_)(Cl)_2_Ru=CHPh (**Gru-II**)^3^ to the phosphine-free Ru complex bearing a bidentate 2-alkoxybenzylidene ligand [(NHC)(2-isopropoxy-κ-*O*-benzylidene)RuCl_2_] (**Hov-II**, Figure 1).^4^ Furthermore, the structural flexibility of these complexes allowed a great adjustability of their properties^5^ such as an increased immunity toward unwanted C=C bond shifting,^6^*E*/*Z* selectivity,^7^ immobilization/heterogenization of molecular complexes,^8^ compatibility with green and aqueous media,^5f^ latency,^9^ and light-activated olefin metathesis.^10^

A new class of complexes emerged with the simple replacement of the chelating oxygen atom in the **Hov-II** structure by other main-group elements such as N,^11^ S,^12−14^ P,^15^ or halogen^16^ atoms (Figure 1b). These catalysts exist in two isomeric forms, *trans*- and *cis*- dichlorido ruthenium complexes, as depicted in Figure 1. Among all of these novel catalysts, the sulfur-chelated Ru-Hoveyda type complexes are some of the most extensively studied, notably by Lemcoff and co-workers.^17^ Of interest, this group demonstrated that the *trans*-Cl_2_ form of these complexes was the active precatalyst in olefin metathesis transformations but readily isomerized in solution to the thermodynamically more stable and inactive *cis*-Cl_2_ complexes.^14a^ The very same group explained this stabilization of the *cis* form by the deleterious strong *trans* influence of the NHC ligand preventing the coordination/stabilization of a second σ-donor ligand in a *trans* position.^15,18^ Remarkably, while all of these *cis*-Cl_2_ catalysts can be activated by heat and exhibit a thermal-switchable behavior, the *cis*-Cl_2_ complex can easily isomerize to the catalytically active *trans* form upon irradiation with UV-A light. Such light-activated catalytic systems were found to be the most reactive in apolar solvents such as benzene due to a higher rate of isomerization and a significant stabilization of the *trans*-Cl_2_ form of the complexes.^18^

Lemcoff and co-workers demonstrated that the reactivity of such *S*-chelated complexes was strongly dependent on the steric^14b,19^ and the electronic properties^14b^ of the *S*-substituent (denoted R in Figure 1) with the reactivity trend CF_3_ > Ph > ^*t*^Bu > ^*i*^Pr > Et > Me^19^ with thermal activation and CF_3_ > Ph > β-naphthyl > 1-pyrenyl > ^*i*^Pr^11g,14b^ with 350 nm irradiation. On the basis of this observation, we assumed that the replacement of the *S* atom by the larger *Se* atom could afford some alteration of the reactivity of the corresponding Ru catalyst in comparison to **Ru4** (S^*i*^Pr).^18,20^ While the synthesis of such a seleno-chelated Ru complex has been already reported by the Lemcoff group,^15^ the reactivity profile of such a system has never been explored.^15^ Indeed, Lemcoff and co-workers synthesized and partially characterized the selenium Hoveyda–Grubbs complex to confirm the theoretical predictions of the favored *cis* conformation of such catalyst but did not study the preferred activation method or the *cis–trans* equilibrium of such a complex. In addition to not being the primary goal of the study, the absence of an in-depth catalytic examination was probably precipitated by the tedious low-yield synthesis of the required selenium-containing benzylidene ligand.

Herein, we report the improved synthesis of the selenium variant of the Hoveyda–Grubbs catalyst and its characterization and catalytic profile, including its latency and activity in selected olefin metathesis benchmark reactions.

## Results and Discussion

**Scheme 1:**
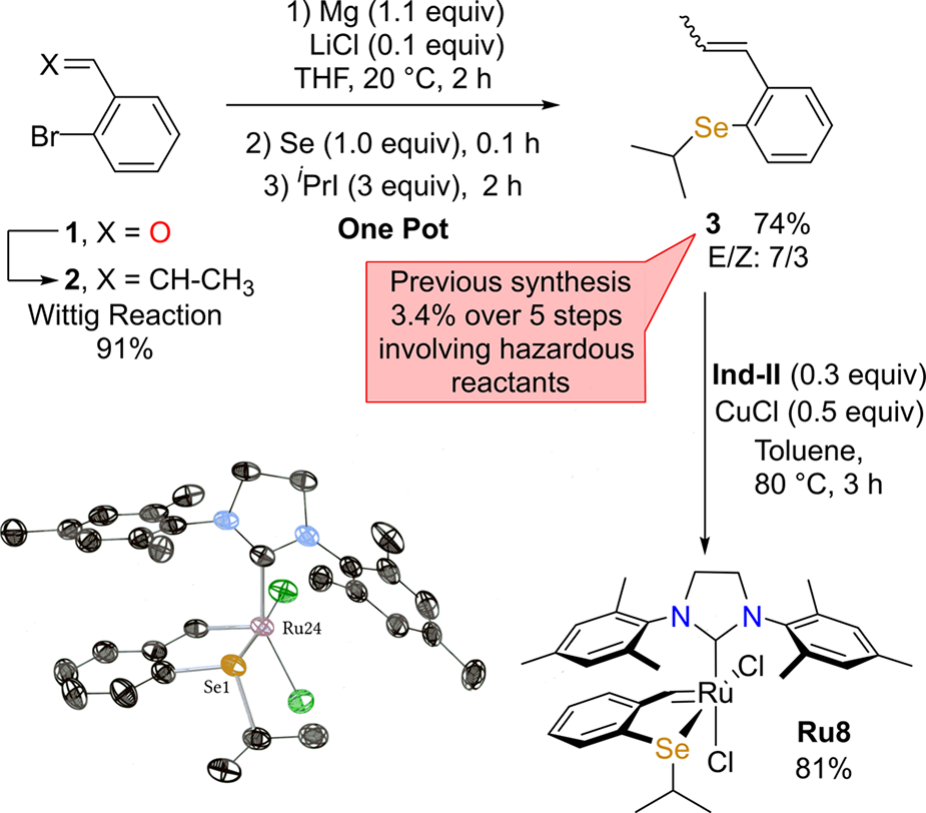
Synthesis of the Selenide **3** and Corresponding Ru Complex **Ru8** and ORTEP Diagram of **Ru8** with 50% Probability Ellipsoids Hydrogen atoms are omitted for clarity.

The first synthesis of the selenium-bearing benzylidene ligand precursor was disclosed in 2009 using methyl 2-aminobenzoate as the starting material.^15,21^ In addition to being laborious and very low-yielding (3.4%), this preparation involved some hazardous compounds such as potassium selenocyanate in acidic media. Furthermore, this pathway included the selenation step at the very beginning of the synthesis and forced the handling of potentially toxic volatile organoselenium compounds.^22^ We thus considered to revisit the synthesis of such a molecule with two main aims in mind: (1) avoiding most of these aforementioned steps and hazardous reactants and also (2) introducing the selenium atom at a late stage of the synthesis. Therefore, we considered the synthesis of selenide **3** from the corresponding bromo derivative **2** via a simple one-step formation of a Grignard reagent and its subsequent reaction with metallic selenium as described by Tang^23^ (Scheme 1). To do so, bromostyrene **2** was treated with 1.1 equiv of Mg chips in freshly distilled THF at room temperature in the presence of a substoichiometric amount of lithium chloride (0.1 equiv).^24^ Once the organomagnesium derivative was formed, the gray selenium (1.0 equiv) was added in one portion followed by an excess of isopropyl iodide (3 equiv).^25^ The product **3** was then obtained in 74% yield with a simple extraction in DCM and could be used without further purification. Of note, the use of 1 equiv of LiCl for the first step of this reaction (instead of 0.1 equiv) proved to be deleterious for the formation of the selenolate intermediates Ar-Se-MgBr and prevented any further reaction. In comparison with the previous synthesis, such a methodology allowed the isolation of the selenide derivative **3** in a 67% yield from the commercially available 2-bromobenzaldehyde **1** and the selenium atom was introduced in the very last step. Of importance, such a late introduction of the heteroatom in the organic synthesis renders it safer than previous syntheses, since no further required step forces the handling and manipulation of potentially toxic volatile organoselenium compounds.

A simple ligand exchange of **3** (1 equiv) with the second-generation ruthenium indenylidene complex **Ind-II** (0.3 equiv) in the presence of CuCl (0.5 equiv) as a phosphine scavenger allowed the isolation of the corresponding *cis*-dichlorido seleno-chelated complex **Ru8** (depicted in Scheme 1) as unique isomer within 81% yield. ^1^H and ^13^C NMR as well as a single-crystal X-ray analysis of **Ru8** confirmed the *cis*-dichlorido geometry. The selenium–ruthenium bond length was 2.4479(7) Å, in line with reported L-type ligand aryl/alkyl–Se–Ru(II) bonds in the Cambridge Crystallographic Database.^15,26^ Of note, ^77^Se NMR spectroscopy confirmed the coordination of the selenium atoms to the Ru(II) center, since the ^77^Se peaks of **3** (δ 368.8 and 379.5 ppm for the *E* and *Z* isomers, respectively) are shifted downfield to δ 518.9 ppm in **Ru8** (Δδ = 150.1 and 139.3 ppm).

The activity and the stability of complex **Ru8** were then probed through the RCM reaction of the benchmark substrate^27^ diethyl diallylmalonate (**4**). The first attempts of an RCM reaction of **4** were undertaken with 1 mol % of **Ru8** (0.5 M in DCM or C_6_D_6_, Table 1, entries 1 and 4). Whether in DCM or in benzene-*d*_6_ (0.5 M), the complex **Ru8** was found to be completely inactive at room temperature. Remarkably, such solutions of **Ru8** in the presence of RCM substrates could be stored for more than 1 month without any sign of reaction or of decomposition of the complex, demonstrating the high latency as well as the exceptional stability of **Ru8**. A further study of this complex led us to consider a thermal activation of **Ru8** as reported by Lemcoff for the sulfur-containing counterpart **Ru4**.^14a,28^ The reaction mixture of **4** in the presence of **Ru8** was thus heated at 40 °C in DCM for 48 h, but no conversion of **4** could be observed under such conditions. When DCM was replaced with benzene, the reaction mixture could be heated to 80 °C and this temperature allowed the activation of **Ru8**, which promoted the RCM of **4** and led to a modest conversion of 29% within 7 h. Of high interest, while the activation of **Ru8** is demonstrated by the conversion of **4**, the *trans*-Cl_2_ species, the active form of this type of catalyst, has never been observed in ^1^H NMR of the crude mixture in such an activation mode (*vide infra* and in the Supporting Information).

**Table 1:**
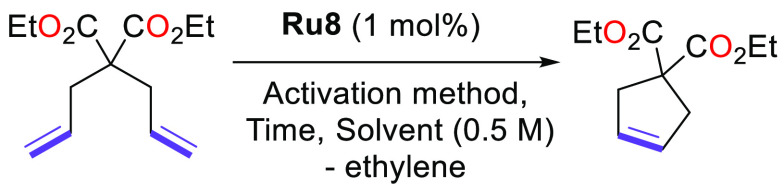
RCM of **4** Promoted by Complex **Ru8** upon Thermal or Light Activation

entry	solvent	activation	time (h)	conversn (%)
1	CD_2_Cl_2_	none, 20 °C	720	<5
2	CD_2_Cl_2_	heat, reflux	48	<5
3	CD_2_Cl_2_	*h*ν, 20 °C	24	25
4	C_6_D_6_	none, 20 °C	720	<5
5	C_6_D_6_	heat, 80 °C	7	29^a^
6	C_6_D_6_	heat, 80 °C	24	63^a^
7	C_6_D_5_CD_3_	heat, 80 °C	24	79
8	C_6_D_5_CD_3_	heat, 110 °C	5	78
9	C_6_D_6_	*h*ν, 20 °C	12	73

a The formation of a side product, namely the product resulting from cycloisomerization, was observed.

A longer reaction time at 80 °C (up to 24 h) allowed an improved final conversion of 63% at the expense of the selectivity of the reaction (entry 6, Table 1). Indeed, in the case of prolonged heating of the **Ru8** solution with **4**, the product of cycloisomerization (ca. 7%) was observed in ^1^H NMR, which is probably due to the decomposition of the Ru species. Replacing benzene with deuterated toluene (C_6_D_5_CD_3_) within the same conditions of temperature (80 °C) and catalyst loading of **Ru8** (1 mol %) offered a conversion rate of the reaction similar to that in benzene, leading to a conversion of 30% after 6 h of heating and an increased final conversion of 79% yield after 24 h at 80 °C (entry 8, Table 1). Gratifyingly, the rate of the reaction dramatically increased when the mixture was heated to 110 °C, offering a conversion of 78% within only 5 h (entry 8, Table 1).

Finally, light irradiation was then considered to activate **Ru8** and promote the RCM of diene **4**. To assess the feasibility of such an activation, a solution of **Ru8** in DCM was first irradiated at 365 nm and the reaction progress was monitored by NMR spectroscopy. Gratifyingly, ^1^H NMR unveiled the formation of the *trans*-Cl_2_ isomer of **Ru8** with an alkylidene proton signal at 17.72 ppm (vs 17.46 ppm for the *cis* isomer). Such a species has been detected in a small quantity of ca. 4% after 3 h of irradiation at 365 nm (*K*_eq_ determined as 4.2 × 10^–2^). On the basis of this successful activation, a mixture of **4** in the presence of 1 mol % of **Ru8** in DCM was placed in a UV reactor and irradiated at 365 nm. Such an activation method allowed the RCM of **4** to occur, leading to a conversion of 25% after 24 h of light irradiation. A longer irradiation time did not considerably improve the conversion of the reaction, since after 48 h only 32% of **4** was consumed. On the other hand, in benzene, RCM of **4** proceeded more quickly and was more productive on exposure to light in comparison to the same transformation in DCM or that activated by heat, allowing for 73% conversion within 12 h. Such a result is consistent with the higher rate of activation of **Ru8** in benzene than in DCM under light irradiation with a *K*_eq_ value determined to be 8.7 × 10^–2^. Of note, ^1^H NMR of the crude RCM reaction mixture in benzene unveiled a similar activation of **Ru8** with an isomerization rate of up to 9% after 3 h of irradiation. After 12 h under such conditions (Table 1, entry 9), the catalyst **Ru8** was completely decomposed and no alkylidene proton signal could be observed in ^1^H NMR, preventing an improvement in the yield upon a longer time of irradiation (24 h). To the best of our knowledge, such light activation has never been described with the *S*-chelated Hoveyda type complex **Ru4** containing an isopropyl moiety as the sulfur substituent. Lemcoff and co-workers showed that **Ru4**, in contrast to the *S*-trifluoromethyl- or *S*-aromatic-functionalized **Ru1**–**2** and **Ru6–7**, did not promote the RCM of diene **4** upon light activation regardless of the reaction conditions of time and solvent.^14b,17^ This successful light activation of **Ru8** demonstrated that, as postulated earlier, the replacement of the sulfur with a selenium atom in the *S*-chelated Hoveyda type complexes afford some additional features, even with the simple ^*i*^Pr moiety as a substituent of the heteroatom.

**Figure 2:**
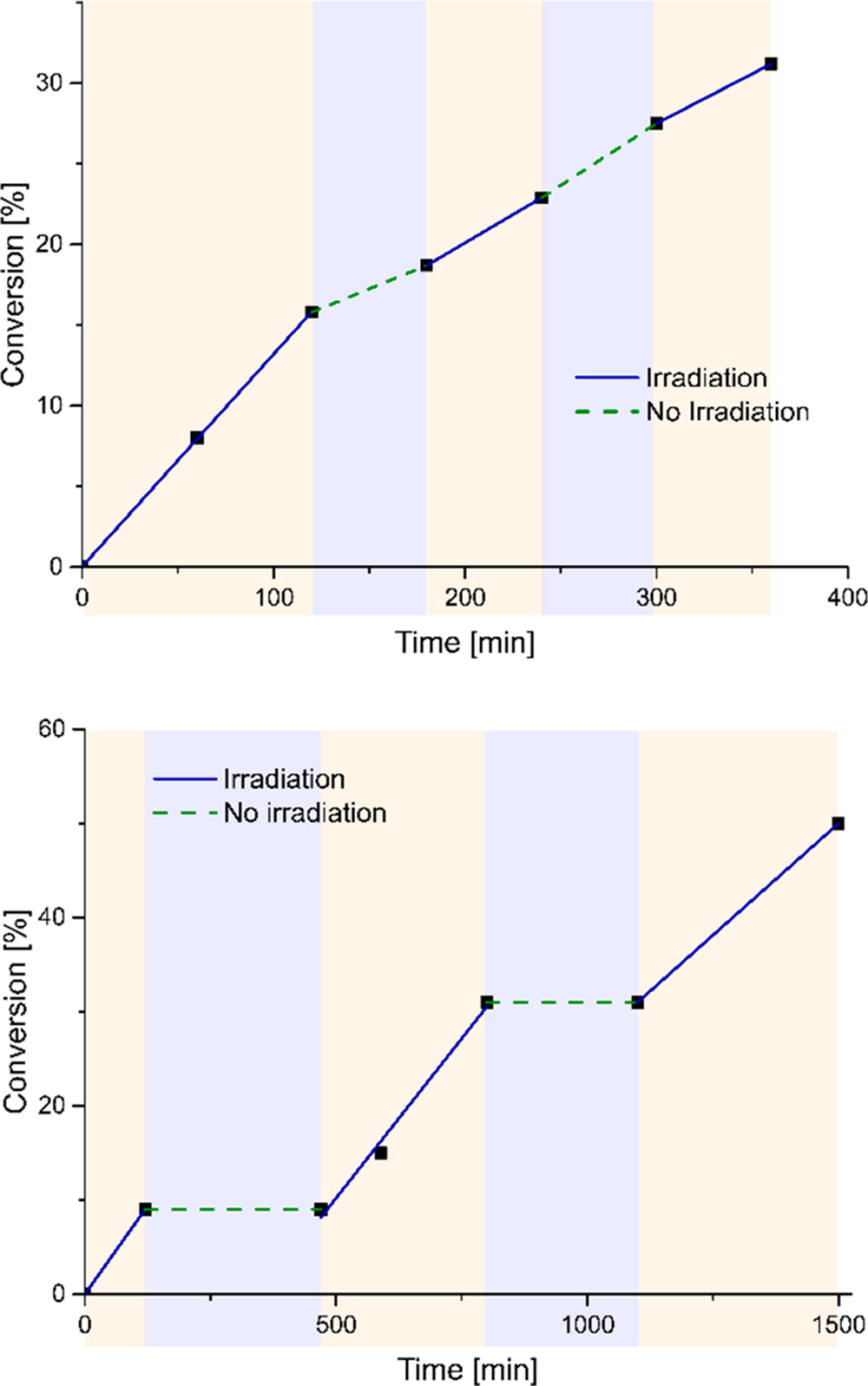
Evolution of the UV-activated RCM of **4** catalyzed by **Ru8** (1 mol %). (top) Cycle of irradiation at 365 nm (blue line) and no irradiation (green dashed line). (bottom) Application of the activation–deactivation protocol: cycle of UV irradiation followed by 5 min of heating at 80 °C (blue line) and resting period at room temperature with no irradiation (green dashed line). The conversion was measured by ^1^H NMR. Lines are visual aids only.

Of interest, in the case of heat activation, complex **Ru8** demonstrated a thermally switchable behavior similar to that of its sulfur-chelating counterpart **Ru4**: i.e., as soon as the reaction mixture was cooled to room temperature, the RCM of **4** was detained and no further evolution could be observed for days at 23 °C. Such a reaction could be resumed by heating the reaction mixture once again at 80 °C, and the complex **Ru8** could sustain several heating–cooling cycles without any sign of decomposition.^14a,28^ Such behavior is consistent with the absence of the *trans*-Cl_2_ form of **Ru8** observed under thermal conditions. In contrast, this switchable behavior of **Ru8** was lost with the irradiation activation mode (Figure 2, top graph). Indeed, even after the end of the irradiation cycle, we could observe that the RCM of **4** was still evolving, as depicted in Figure 2 (top graph). This absence of switchable behavior upon light activation of **Ru8** has been attributed to the persistence of the reactive *trans*-Cl_2_ isomer in benzene,^1b,2,10^ which exhibited a half-life time of *trans*-**Ru8** of ca. 6 h.^11c,11g,14b,29^ The switchable behavior of **Ru8** under the irradiation mode of activation could be reached only through a catalyst activation–deactivation protocol elegantly developed by Lemcoff and co-workers.^14b^ The latter group proposed that heating the reaction mixture after the irradiation cycle with UV would “turn off” the RCM of **4** by forcing the isomerization of the *trans*-Cl_2_ catalyst to the thermodynamically more stable inactive *cis*-Cl_2_ species. To do so, the reaction mixture was simply heated at 80 °C for a short period of time of 5 min after each light activation cycle (Figure 2, bottom graph). Gratifyingly, in the case of the RCM of **4** catalyzed by **Ru8**, all *trans* species isomerized back to the metathesis-inactive *cis*-Cl_2_ complex and the reaction stopped. As in the case of the thermal-switchable reaction, the reaction conversion resumed with a new cycle of irradiation at 365 nm as, depicted in Figure 2.

The reactivity and the latency of the *cis*-dichlorido complex **Ru8** were further probed through a series of ROMP reactions with different monomers offering variable ring strain, such as cyclooctene (COE, low ring strain) and dicyclopentadiene (DCPD, high ring strain). First, the ROMP of COE was carried out under several conditions of activation methods, solvents, and catalyst loadings, as reported in Table 2. The catalyst **Ru8** demonstrated a perfect latency for the ROMP of COE with 150 ppm (0.015 mol %) catalyst loading in toluene (0.5 M), since no conversion of COE could be observed in ^1^H NMR or GC without irradiation (Table 2, entry 1). On the other hand, when the reaction mixture was exposed to UV, the ROMP of COE reached completion within 2 h, yielding the corresponding polymer in quantitative yield (*E*/*Z* ratio 3.5/1, *M*_n_ = 188.5 kDa, PDi = 2.21). When similar reactions were undertaken under neat conditions (150 ppm of **Ru8**), the catalyst conserved its property of latency (Table 2, entry 3) and the monomer was consumed only under light activation within 1 h (Table 2, entry 4). In both cases, ROMP of COE with **Ru8** did not offer well-defined polymeric structures, since high polydispersity indexes from 1.74 to 2.21 were observed. Such a range of PDi can be explained by a high propagation rate and a slow initiation rate of the reaction, which is in line with the aforementioned results with regard to the very slow activation of the catalyst (*vide supra*).^30^

**Table 2:**
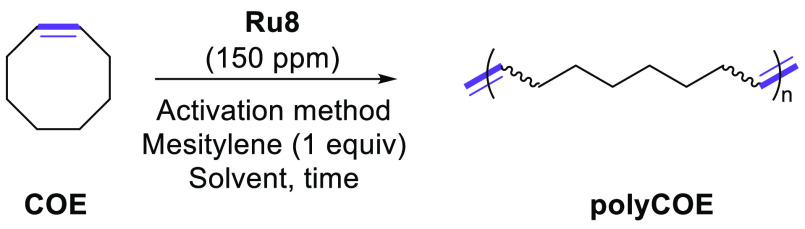
Evaluation of the Performance of **Ru8** in ROMP of COE

entry	activation	solvent	time (h)	conversn^a^^,^^b^ (%)	*E*/*Z*^a^	theor *M*_n_ (kDa)	*M*_n_ (kDa)	PDi
1	dark	Tol	72	<5				
2	*h*ν	Tol	2	>99	3.5/1	726	188.4	2.21
3	dark	neat	72	<5				
4	*h*ν	neat	1	>99	4/1	726	173.9	1.74
5	*h*ν	DCM	20	37	nd	271	6445	2.26
6	80 °C	neat	72	>99	nd	726	127	≥7.66
7^c^	*h*ν	neat	24	82	nd	9900	21.5	2.64

a Determined by ^1^H NMR spectroscopy. nd denotes not determined.

b The conversion is the average value of at least two reactions. GPC measurements have been performed on one sample for each entry.

c 10 ppm of **Ru8**.

Replacing toluene with DCM as well as changing the activation source to heat (80 °C) dramatically affected the outcome of the reaction (Table 2, entries 5 and 6). When DCM was used as the solvent for the ROMP of COE (150 ppm of **Ru8**), the conversion of the starting material did not exceed 37%, which is reminiscent of the low conversion of **4** in DCM presented in Table 1. In contrast, heating a reaction mixture of COE and catalyst **Ru8** under neat conditions at 80 °C did not prevent the reaction from reaching completion (99% conversion within 72 h). Nevertheless, this activation method dramatically affected the size distribution of the polymers, since the polydispersity index of the corresponding polymer was ≥7.66 (vs 1.74 in the case of light activation). Here again, this high value of PDi is in agreement with the very low activation rate of the catalyst, as previously observed for the RCM reaction of diallylmalonate **4** at 80 °C with catalyst **Ru8**, for which the *trans*-Cl_2_ form of **Ru8** was not detectable in ^1^H NMR (see Table 1, entry 5). Furthermore, such a high PDi value could indicate the presence of a side reaction with a high kinetic rate, such as isomerization of the C=C bonds as previously described in the thermal activation of **Ru8**. It is noteworthy that the catalyst loading was decreased to as little as 10 ppm for the ROMP reaction of COE under neat conditions, and the reaction still offered a satisfactory yield of polyCOE of 82%.

**Scheme 2:**
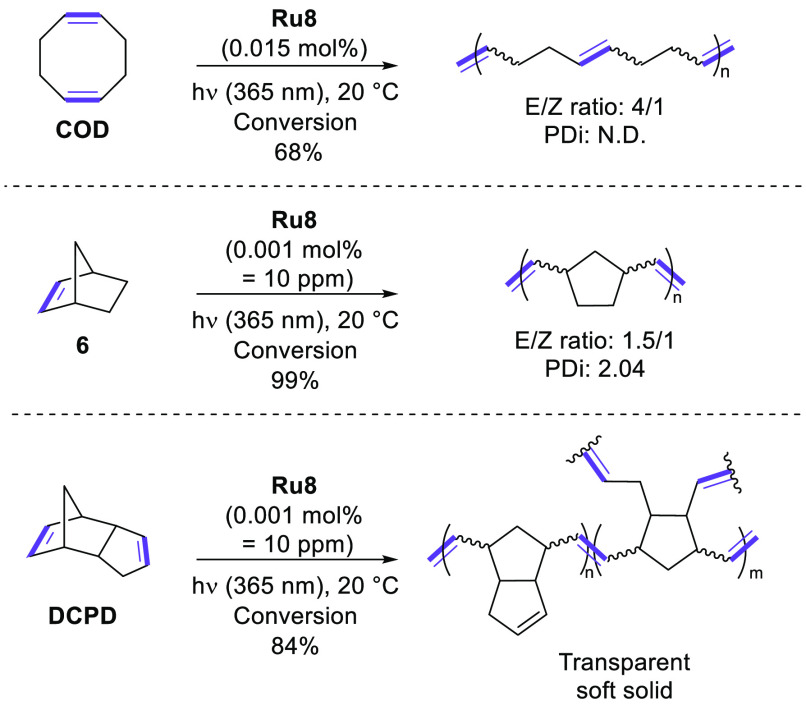
Scope of the ROMP with **Ru8** with Light Activation N.D. denotes not detected.

Using **Ru8** (150 ppm) under neat conditions in the presence of 1 equiv of mesitylene as an internal standard, we decided to study the scope of the ROMP reaction. First, cyclooctadiene (COD) was selected for this study. While COD is structurally similar to COE and exhibits a higher ring strain, the ROMP of COD (Scheme 2) with light activation proved to be less efficient than that of COE, leading to a maximum yield of 68% after 6 h of reaction. Moreover, while ^1^H NMR spectra confirmed the formation of the corresponding polymer, its size distribution was so broad that we were not able to determine the corresponding polydispersity index. On the other hand, monomers presenting a higher ring constraint in comparison to COE and COD were found to be very reactive under the conditions depicted in Scheme 2. Indeed, within only 1 h of irradiation and with 150 ppm of **Ru8**, the reaction of polymerization of both norbornene (**6**) and DCPD reached completion (vs 2 h for COE and 6 h for COD). In the case of the highly reactive monomer **6**, the catalyst loading could be decreased to as little as 10 ppm without any deletion of the conversion (Scheme 2). Indeed, within 12 h of irradiation, the very low loading of **Ru8** allowed the reaction to reach completion and the polydispersity was determined to be 2.04. Such PDi is higher than that of COE (1.74), in accordance with the respective ring constraints and consequently the respective reactivities of the monomers. Finally, the reaction of DCPD with 150 ppm of **Ru8** led to the isolation of a rigid colorless material demonstrating the cross-linking of the corresponding polymers. When the catalyst loading was decreased to 10 ppm, the ROMP reaction of DCPD did not reach completion (84% conversion within 12 h) and led to the isolation of a soft solid.

**Scheme 3:**
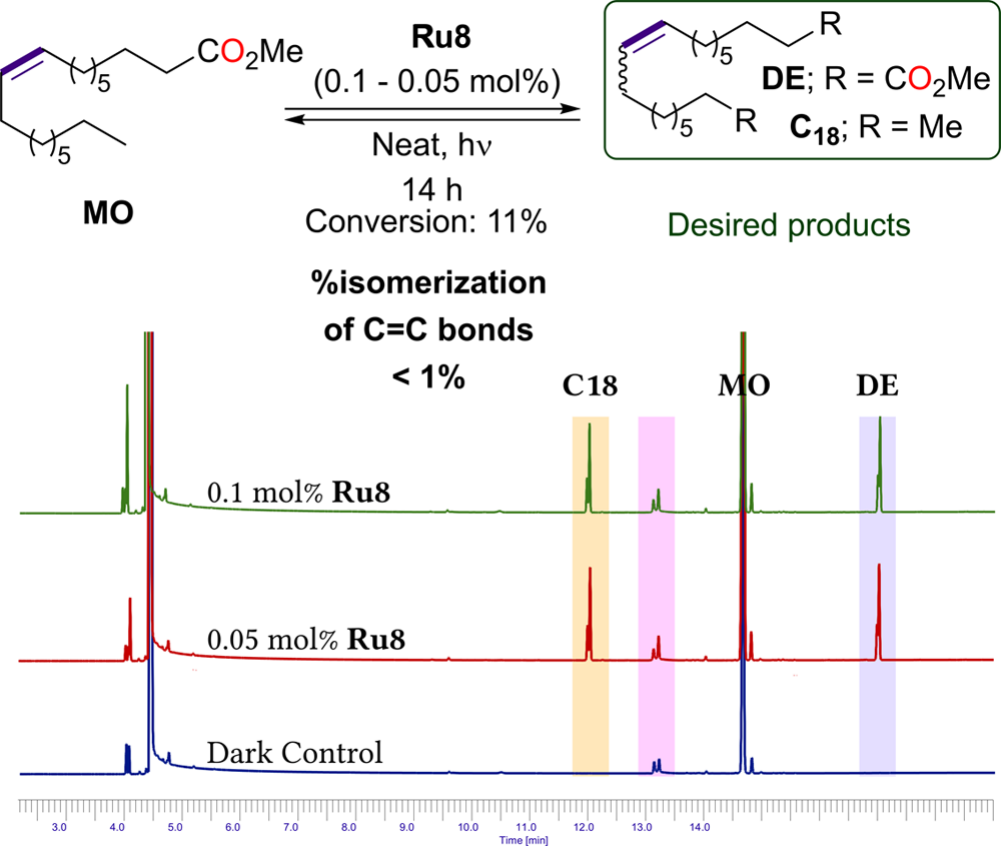
Self-CM of Methyl Oleate (**MO**) Promoted by **Ru8** under Light Activation GC traces of the crude reaction. In pink, presence of contamination in the starting material.

On the basis of the observed stability and reactivity of the complex **Ru8**, the industrially relevant self-CM of methyl oleate (**MO**) was then considered. The group of Lemcoff demonstrated that the light activation used for self-CM was extremely beneficial to avoid a fast catalyst decomposition and removed the main limitation of the efficiency of these reactions: the isomerization of the double bonds (C=C bonds shifting, caused by Ru-decomposition products) leading to a complex mixture of products.^14f^ Indeed, in a previous work, Lemcoff and co-workers used the trifluoromethyl-substituted sulfur-chelating Hoveyda type complex **Ru1** to promote this reaction under light activation and achieved a perfect selectivity toward unwanted C=C bond shifting of the self-CM, yielding only the corresponding products: octadec-9-ene (**C18**) and the diester (**DE**) (no homologues of **C18** as well as **DE** were observed). We wanted to compare the outcome of the reaction of **MO** catalyzed by **Ru1** with the equilibrium obtained when **Ru8** was used as a catalyst (Scheme 3).

To do so, the self-CM of **MO** was undertaken under conditions similar to those previously reported: 1000 ppm of catalyst under neat condtions and under irradiation for 14 h (Scheme 3). Satisfyingly, the GC traces unveiled a very high selectivity (>99%) toward the expected products **C18** and **DE**, since no isomerization products of the C=C bond shifting could be observed (Scheme 3). Of note, when the catalyst loading was decreased to 500 ppm (0.05 mol %), the same selectivity was obtained, demonstrating the robustness of such an activation method for the self-CM of **MO**.

## Conclusions

In summary, we reported herein a novel, straightforward synthesis of the selenium-containing styrene derivative **3** in an excellent overall yield of 67% starting from commercially available 2-bromobenzaldehyde. The synthesis of the corresponding complex **Ru8** was successfully achieved (81% yield) and thus allowed us to study in detail its latency, activation, and reactivity through a set of benchmark RCM, ROMP, and self-CM reactions. Of interest, while the corresponding *S*-chelated Ru complex **Ru4** was reported to be activated only by heat, complex **Ru8** demonstrated its versatility upon both thermal and light activation. Similarly to **Ru4**, the seleno-chelated ruthenium catalyst exhibited a thermal-switchable behavior for the RCM of diethyl diallylmalonate. On the other hand, on activation by irradiation, **Ru8** lost its switchable property due to the persistence of the active *trans*-Cl_2_ form of this selenium-containing catalyst in benzene. The latency of **Ru8** was proven as well in the ROMP of COE and self-CM of methyl oleate. In all of these reactions, **Ru8** was found to be very active and efficiently promoted polymerization reactions of norbornene and COE with a catalyst loading as low as 10 ppm. Furthermore, the self-CM of methyl oleate demonstrated an excellent selectivity of >99% toward C=C bond shifting.

We demonstrated that the replacement of the *S* atom with *Se* in the Hoveyda–Grubbs type structure endowed the resulting catalyst with an interesting behavior of light activation. Further development of such seleno-chelated complexes may follow the same successful strategy that was used by Lemcoff and co-workers for the sulfur-chelating Hoveyda–Grubbs family catalysts: variation of the selenoether fragment in the benzylidene ligand, such as the introduction of phenylselenolate or trifluoromethylselenolate in place of Se^*i*^Pr in **Ru8**.

## Experimental Section

### General Remarks

Unless otherwise noted, all materials were purchased from commercial suppliers and used as received. All reactions requiring exclusion of oxygen and moisture were carried out in dry glassware with dry solvents (purified using the MBraun SPS) under a dry and oxygen-free atmosphere using Schlenk technique. The bottles with ruthenium catalysts were stored under argon atmosphere, but no special precautions were taken to avoid air or moisture exposure in the moment of extracting catalysts from the bottles. Nuclear magnetic resonance (NMR; ^1^H, ^13^C, and ^77^Se) spectra were recorded on Agilent Mercury 400 MHz spectrometer at ambient temperature with CDCl_3_ or CD_2_Cl_2_ as the solvent. Chemical shifts (δ) are given in parts per million (ppm) downfield from tetramethylsilane with a residual undeuterated solvent peak used as a reference: CDCl_3_ (δ_H_ 7.26 ppm, δ_C_ 77.16 ppm), CD_2_Cl_2_ (δ_H_ 5.32 ppm, δ_C_ 53.84 ppm). Coupling constants (*J*) are reported in hertz (Hz) and refer to *H*,*H* couplings. The following abbreviations are used in order to indicate the multiplicity of the signal: s (singlet), d (doublet), t (triplet), q (quartet), quin (quintet), sext (sextet), hept (heptet), dd (doublet of doublets), dt (doublet of triplets), ddd (doublet of doublets of doublets), etc., bs (broad signal), m (multiplet). The data obtained were processed with the software MestReNova. Gas chromatography (GC) measurements were carried out using a PerkinElmer Clarus 580 instrument employing an InertCap 5MS-Sil column and helium as a carrier gas. Infrared (IR) spectra were recorded using a JASCO FT/IR-6200 spectrometer. The substances were examined as a film or in solution. Wavenumbers (ν̃) are reported in cm^–1^. The data obtained were processed with the software Omni32. High-resolution mass spectrometry (HRMS) measurements were carried out using an AutoSpec Premier spectrometer. Column chromatography was performed using Merck Millipore silica gel (60, particle size 0.043–0.063 nm). Size exclusion chromatography was performed by a specialized laboratory at the University of Warsaw (Dr. Elżbieta Megiel, Faculty of Chemistry, Poland), using a Shimadzu Nexera high-pressure liquid chromatograph (Shimadzu Corporation) in THF at 40 °C with a flow rate of 1.0 mL min^–1^. Two columns placed in series were used: ReproGel 1000 and 10000 (Dr. Maisch), 5 μm, 300 mm × 8 mm. Samples were detected with a Shimadzu Model RID-20A refractive index detector.

### 1-Bromo-2-(prop-1-en-1-yl)benzene (**2**)

Ethyltriphenylphosphonium bromide (8.7 g, 23.1 mmol, 2.3 equiv) and potassium *tert*-butoxide (2.7 g, 23.1 mmol, 2.3 equiv) were placed in an oven-dried flask. A 20 mL portion of anhydrous THF was added. The reaction mixture was stirred at room temperature for 30 min. Then the mixture was cooled to −78 °C and the appropriate aldehyde **1** (1.9 g, 10.1 mmol, 1 equiv) was added dropwise. Stirring was continued over 2 h at rt. The reaction mixture was quenched with a saturated aqueous solution of NH_4_Cl (50 mL), and the THF was removed under reduced pressure. DCM (50 mL) was added, and the layers were separated. The aqueous phase was extracted with DCM (2 × 50 mL). The organic layer was dried over anhydrous MgSO_4_, and afterward, the solid was filtered off and the organic solution was concentrated by the evaporation of DCM. The crude product was purified using quick column chromatography (stationary phase SiO_2_, eluent DCM) in order to remove triphenylphosphine oxide from the alkene. The product was obtained as a yellow oil (1.81 g, 91%) and could be used without further purification. Further purification was performed by distillation using a Kugelrohr apparatus (*p* ≈ 10 mbar, *T* = 145 °C) to give pure product **2** (1.55 g, 78%, *E*/*Z* = 1.7:1). Major isomer (*E*): ^1^H NMR (400 MHz, CDCl_3_) δ 7.53 (dd, *J* = 8.0, 1.3 Hz, 1H, H_Ar_), 7.47 (dd, *J* = 7.8, 1.7 Hz, 1H), 7.27–7.20 (m, 1H), 7.06 (ddd, *J* = 7.9, 7.3, 1.7 Hz, 1H), 6.74 (dq, *J* = 15.7, 1.8 Hz, 1H), 6.19 (dq, *J* = 15.6, 6.7 Hz, 1H), 1.93 (dd, *J* = 6.7, 1.8 Hz, 3H); ^13^C NMR (101 MHz, CDCl_3_) δ 137.8, 132.9, 130.1, 129.0, 128.2, 127.5, 126.9, 123.1, 18.8.

### Isopropyl(2-(prop-1-en-1-yl)phenyl)selane (**3**)

In a glovebox filled with argon, a 25 mL round-bottom flask was charged with Mg (finely ground, 26 mg, 1.1 mmol, 1.1 equiv), lithium chloride (4.8 mg, 0.1 mmol, 0.1 equiv), and THF (3 mL). To the resulting suspension was added a solution of bromostyrene derivative **2** (200 mg, 1.0 mmol, 1.0 equiv) in THF (2 mL) dropwise. The reaction mixture was stirred at room temperature until the complete consumption of magnesium (leading to a clear colorless to pale yellow solution). A Grignard reagent was thus formed, and gray Se (80 mg, 1.0 mmol, 1.0 equiv) was added in one portion with vigorous stirring. The dark suspension of selenium in the reaction mixture turned rapidly (after 15 min) into a homogeneous clear orange solution, indicating the full conversion of selenium. The flask was taken outside of the glovebox, and 2-iodopropane (509 mg, 3 mmol, 3 equiv) was added to the reaction mixture. After the addition of alkylating agent, a precipitate appeared instantly, leading to a gray to yellow turbid suspension. The reaction mixture was stirred at room temperature overnight, and afterward water was added (5 mL). The product was extracted with DCM (3 × 10 mL). The organic phases were combined, washed with brine, and dried over anhydrous Na_2_SO_4_. The volatiles were removed under reduced pressure, and the yellow oil was dried overnight under high vacuum (1 × 10^–3^ mbar). The product **3** was obtained as a yellow oil (177.6 mg, 74%, *E*/*Z* = 1.7:1) and could be used without further purification. Major isomer (*E*): ^1^H NMR (400 MHz, CDCl_3_) δ 7.55 (dd, *J* = 7.7, 1.1 Hz, 1H), 7.47 (dd, *J* = 7.8, 1.2 Hz, 1H), 7.13 (m, 2H), 6.95 and 6.79 (dd, *J* = 15.6, 1.5 Hz, 1H), 6.22–6.04 (m, 1H), 3.41 (sept, *J* = 6.8 Hz, 1H), 1.91 (dd, *J* = 6.6, 1.7 Hz, 3H) 1.39 (d, *J* = 6.8 Hz, 6H); ^13^C NMR (101 MHz, CDCl_3_) δ 141.2, 135.8, 131.4, 127.9, 127.6, 127.15, 126.8, 33.9, 24.2, 18.8, 14.5, 14.4; ^77^Se NMR (76 MHz, CDCl_3_) δ 368.82. HRMS (ESI): calculated mass for C_12_H_17_Se [M + H]^+^, 241.0490; found, 241.0490(3).

### Catalyst **Ru8**

A dry Schlenk vessel was charged with ruthenium complex **Ind-II** (100 mg, 0.105 mmol, 1 equiv) under an argon atmosphere. Then, 15 mL of anhydrous toluene was added, resulting in a deep red solution. Selenium-containing styrene **3** (62 mg, 0.315 mmol, 3 equiv) and anhydrous CuCl (15 mg, 0.158 mmol, 1.5 equiv) were added to the reaction mixture, and then it was stirred for 3 h at 80 °C. Complete conversion of the **Ind-II** catalyst was observed using TLC (stationary phase SiO_2_, eluent EtOAc/*n*-hexane 10/90 v/v, *R*_f,**Ind-II**_ = 0.39, *R*_f,**Ru8**_ = 0) after that time. All of the volatiles were removed under reduced pressure, and the crude product was purified using column chromatography (stationary phase SiO_2_, eluent EtOAc/*n*-hexane from 20/80 to 80/20 v/v). The product complex **Ru8** was obtained as a green solid (67 mg, 81%). ^1^H NMR (400 MHz, CD_2_Cl_2_): δ 17.47 (s, 1H), 7.52–7.41 (m, 2H), 7.20 (ddd, *J* = 7.7, 5.4, 3.0 Hz, 1H), 7.12 (s, 1H), 7.01 (s, 1H), 6.89 (s, 1H), 6.83 (d, *J* = 7.5 Hz, 1H), 5.94 (s, 1H), 4.25–3.72 (m, 5H), 2.67 (s, 1H), 2.53 (s, 1H), 2.47 (s, 1H), 2.36 (s, 1H), 2.15 (s, 1H), 1.54 (d, *J* = 4.4 Hz, 4H), 1.30 (t, *J* = 5.0 Hz, 2H), 1.02 (d, *J* = 6.8 Hz, 1H). ^13^C NMR (101 MHz, CD_2_Cl_2_): δ δ 284.6, 215.0, 157.5, 140.3, 140.2, 138.4, 137.8, 136.8, 135.4, 132.4, 131.8, 130.7, 129.8, 129.7, 129.6, 129.2, 128.7, 124.9, 51.6, 51.3, 36.4, 23.7, 21.9, 21.1, 20.9, 20.3, 20.1, 18.7, 17.8. ^77^Se NMR (76 MHz, CD_2_Cl_2_): δ 518.9.

### General Procedure for the RCM Reaction of **4**

In a glovebox filled with argon, diene **4** (48.5 mg, 50 μL, 0.2 mmol, 1 equiv) was added to a solution of **Ru8** (1.38 mg, 0.002 mmol, 1 mol %) in the appropriate solvent (0.4 mL) in a screw-cap NMR tube. The NMR tube was taken outside of the glovebox, and the reaction mixture was stirred at 20 °C or heated or placed in a UV reactor for the required time. Data were recorded using 32 scans with a D1 delay time minimum of 5 s between each pulse. The progress of the reaction was monitored through the disappearance of the methylene signals of **4** (2.63 ppm) and the growth of the methylene proton signal of the product **5** (3.01 ppm).

### General Procedure for the ROMP Reactions

A 15 mL vial equipped with a screw cap was charged with a cyclic substrate (1 equiv). Unless the reaction was carried out under neat conditions, an appropriate solvent (0.5 M) was added (DCM or toluene). To the resulting solution was added mesitylene (1 equiv) as an internal standard followed by the addition of a **Ru8** catalyst stock solution in DCM (1 M, 0.0150 or 0.001 mol %). For the reactions carried out under neat conditions, the solvent (DCM) was removed under reduced pressure before photochemical activation. The vial was then placed in a UV reactor (365 nm) or in a heated oil bath (80 °C) and was left for the required time. The NMR spectrum of the crude solution was recorded, and it highlighted the presence of the polymer. The resulting polymer was precipitated out with acetone and methanol and then filtered off.

### General Procedure for the Reaction of Self-CM of **MO**

Methyl oleate (**MO**) (341 μL, 1 mmol) was introduced into a 1.5 mL screw-capped vial. The air in the flask was replaced by argon. The catalyst (0.69 mg, 0.001 equiv, 1 μmol, 0.1 mol % or 0.35 mg, 0.5 μmol, 0.05 mol %) was added as a CH_2_Cl_2_ solution. CH_2_Cl_2_ was evaporated from the vessel by high vacuum followed by refilling the vial with argon before placing the vial in a UV reactor. Then, the reaction mixture was irradiated for 14 h. After this time, a solution of SnatchCat was added to the reaction mixture and diluted in toluene. The resulting solution was injected into the GC-MS.

## Supporting Information Available

Additional experimental details, crystal data for **Ru8**, and NMR spectra for compounds **3** and **Ru8** (PDF)

## Accession Codes

CCDC 2105961 contains the supplementary crystallographic data for this paper. These data can be obtained free of charge via www.ccdc.cam.ac.uk/data_request/cif, or by emailing data_request@ccdc.cam.ac.uk, or by contacting The Cambridge Crystallographic Data Centre, 12 Union Road, Cambridge CB2 1EZ, UK; fax: +44 1223 336033.
