# Supplementary material for: Synthesis and Catalytic Properties of a Very Latent Selenium-Chelated Ruthenium Benzylidene Olefin Metathesis Catalyst
Source: Organometallics. 2021 Oct 25;40(21):3608–16. doi: 10.1021/acs.organomet.1c00484 (PMC8579520; doi:10.1021/acs.organomet.1c00484)
Supplement: Supplementary file 1 — om1c00484_si_001.pdf [file om1c00484_si_001.pdf]

Synthesis and Catalytic Properties of a Very Latent Selenium-Chelated Ruthenium Benzylidene Olefin Metathesis Catalyst

Louis Monsigny[a], Joel Cejas Sánchez[a], Jakub Piątkowski[a], Anna Kajetanowicz[a] and Karol Grela*[a]*

[a] Biological and Chemical Research Centre, Faculty of Chemistry, University of Warsaw, Żwirki i Wigury 101, 02-089 Warsaw, Poland

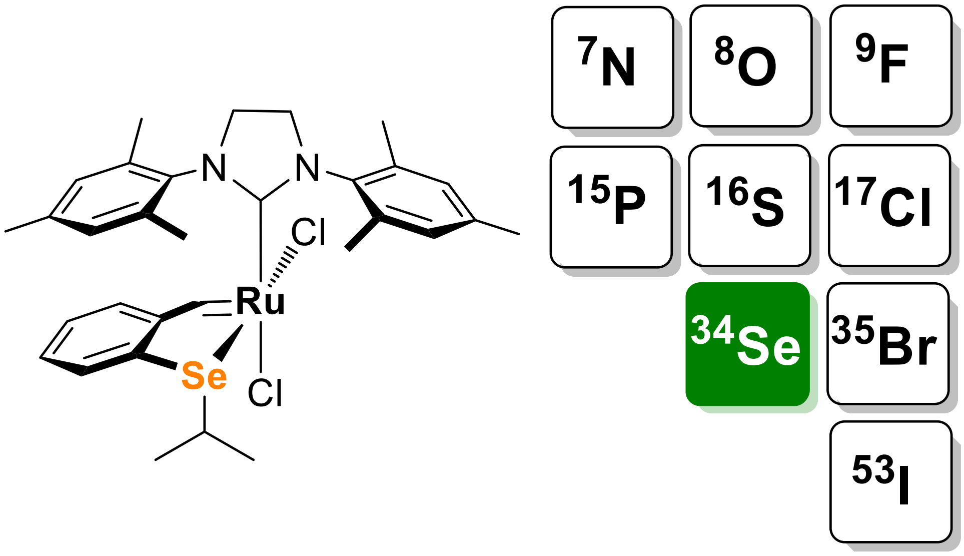

Supporting Information

l.monsigny@cent.uw.edu.pl
joel.cejassanchez@gmail.com
j.piatkowski2@student.uw.edu.pl
a.kajetanowicz@uw.edu.pl
prof.grela@gmail.com

Table of Contents

Experimental Details 3
Synthesis of the Ligand 5
Synthesis of the Ruthenium Complex 7
X-Ray Data Collection and Structure Refinement 8
Activation of **Ru8** and Catalytic Activity 15
Demonstration of **Ru8** Latency 15
Activation of **Ru8** in CD_2Cl_2 with UV-Irradiation 16
Activation of **Ru8** in C_6D_6 with UV-Irradiation 17
Activity of **Ru8** in RCM Reactions with Thermal Activation in C_6D_6 18
Activity of **Ru8** in RCM Reactions with Thermal Activation in $C_6D_5CD_3$ 20
Activity of **Ru8** in RCM Reactions with Photochemical Activation in DCM 23
Activity of **Ru8** in RCM Reactions with Photochemical Activation in Benzene 24
Polymerization Reactions Catalyzed by **Ru8** 26
Polymerization of Cyclooctene (**COE**) 26
Polymerization of Dicyclopentadiene (**DCPD**) 30
Polymerization of Norbornene 31
Polymerization of Cycloocta-1,5-diene (**COD**) 33
Self Cross-Metathesis of Methyl Oleate (**MO**) 34
NMR Spectra of New Compounds 39

Experimental Details

General Remarks: unless otherwise noted, all materials were purchased from commercial suppliers and used as received. All reactions requiring exclusion of oxygen and moisture were carried out in dry glassware with dry solvents (purified using *MBraun* SPS) under a dry and oxygen-free atmosphere using Schlenk technique. The bottles with ruthenium catalysts were stored under argon atmosphere, but no special precautions were taken to avoid air or moisture exposure in the moment of extracting catalysts from the bottles.

Nuclear Magnetic Resonance (NMR): NMR (^{1}H and ^{13}C) spectra were recorded on *Agilent* Mercury 400 MHz spectrometer at ambient temperature with $CDCl_3$ or CD_2Cl_2 used as a solvent. Chemical shifts (δ) are given in parts per million (ppm) downfield from tetramethylsilane with a residual non-deuterated solvent peak used as a reference: $CDCl_3$ (δ_H = 7.26 ppm, δ_C = 77.16 ppm), CD_2Cl_2 (δ_H = 5.32 ppm, δ_C = 53.84 ppm). Coupling constants (*J*) are reported in hertz (Hz) and refer to *H,H*-couplings. The following abbreviations are used in order to indicate the multiplicity of the signal: s (singlet), d (doublet), t (triplet), q (quartet), quin (quintet), sext (sextet), hept (heptet), dd (doublet of doublet), dt (doublet of triplet), ddd (doublet of doublet of doublet) etc., bs (broad signal), m (multiplet). The obtained data was processed with the software MestReNova.

Gas Chromatography (GC): GC measurements were carried out using *PerkinElmer Clarus 580* employing InertCap 5MS-Sil column and helium as a carrier gas.

Infrared Spectroscopy (IR): IR spectra were recorded using JASCO FT/IR-6200 spectrometer. The substances were examined as a film or in the solution. Wavenumbers ($\tilde{\upsilon}$) are reported in cm^{-1}. The obtained data was processed with the software Omni32.

Ultraviolet-Visible Spectroscopy (UV-Vis): UV-Vis spectra were recorded using *Thermo Fischer Scientific Evolution* 300 UV-Vis spectrophotometer in 10.00 mm QS cuvettes. Wavelengths (λ) are reported in nanometers (nm), whereas absorbance (*A*) is given in absorbance units (a.u.). Scan speed was set to 600 nm/min, range 300 – 500 nm, bandwidth 1 nm and data interval 1 nm.

High Resolution Mass Spectrometry (HRMS): HRMS measurements were carried out using AutoSpec Premier spectrometer.

Column Chromatography (CC): CC was performed using *Merck* Millipore silica gel (60, particle size 0.043 – 0.063 nm).

Thin Layer Chromatography (TLC): TLC was performed using Merck Silica Gel 60 F_{254} precoated aluminum sheets. Substances were visualized using UV-light (254 or 365 nm) or by stains after $KMnO_4/K_2CO_3$/NaOH reagent treatment (aqueous solution).

Size Exclusion Chromatography (SEC or GPC): Number-average molecular weights (Mn), weight-average molecular weight (Mw), and molecular weight distribution (Mw/Mn) were determined by a specialized laboratory at the University of Warsaw (Dr. Elżbieta Megiel, Faculty of Chemistry, Poland), using a Schimadzu Nexera's high-pressure liquid chromatograph (Shimadzu Corporation) in THF at 40 °C with a flow rate of 1.0 mL.min^{-1}. Two columns placed in series were used: ReproGel 1000 and 10000, (Dr. Maisch) 5 μm, 300 mm x 8 mm. Samples were detected with a refractive index detector

using a Shimadzu model RID-20A. The system was calibrated using poly(styrene) standards (PSS GmbH) in the range of molecular weights 2.09-323 kDa. Molecular weight analysis by Size Exclusion Chromatography (SEC) has been performed from samples dissolved in THF (1 mg/mL) followed by filtration on Pall PTFE membrane filters (0.2 μm); 20 uL injections were applied. The polymers were analyzed after the removal of the catalyst and unreacted monomers by precipitation of the polymers and filtration with methanol.

Synthesis of the Ligand

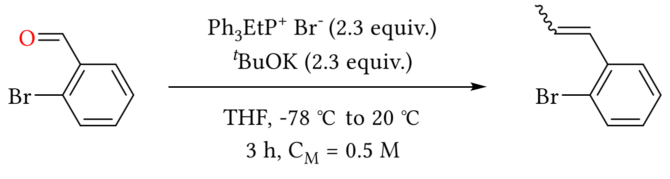

1
(1.9 g, 10.1 mmol)

2
(91%, *E*/*Z* = 1.7:1)

Scheme S1. Synthesis of bromostyrene derivative **2** *via* Wittig olefination.

Ethyltriphenylphosphonium bromide (8.7 g, 23.1 mmol, 2.3 equiv.) and potassium *tert*-butoxide (2.7 g, 23.1 mmol, 2.3 equiv.) were placed in an oven-dried flask. 20 mL of anhydrous THF was added. Reaction mixture was stirred at room temperature for 30 minutes. Then the mixture was cooled down to -78 °C and appropriate aldehyde **1** (1.9 g, 10.1 mmol, 1 equiv.) was added dropwise. Stirring was continued over 2 h at rt. The reaction mixture was quenched with saturated aqueous solution of NH_4Cl (50 mL) and the THF was removed under reduced pressure. DCM (50 mL) was added and the layers were separated. Aqueous phase was extracted with DCM (2 x 50 mL). Organic layer was dried over anhydrous $MgSO_4$ and afterwards, the solid was filtered off and the organic solution was concentrated by the evaporation of DCM. Crude product was purified using quick column chromatography (stationary phase: SiO_2, eluent: DCM) in order to remove triphenylphosphine oxide from the alkene. The product was obtained as a yellow oil (1.81 g, 91%) and can be used without further purification.

It is possible to further purified compound **2** by distillation using Kugelrohr apparatus (p ~ 10 mbar, T = 145 °C) to give pure product **2** (1.55 g, 78%, *E*/*Z* = 1.7:1).

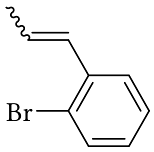

Major Isomer (*E*): ^{1}H NMR (400 MHz, $CDCl_3$) δ: 7.53 (dd, J = 8.0, 1.3 Hz, 1H), 7.47 (dd, J = 7.8, 1.7 Hz, 1H), 7.27 – 7.20 (m, 1H), 7.06 (ddd, J = 7.9, 7.3, 1.7 Hz, 1H), 6.74 (dq, J = 15.7, 1.8 Hz, 1H), 6.19 (dq, J = 15.6, 6.7 Hz, 1H), 1.93 (dd, J = 6.7, 1.8 Hz, 3H). **^{13}C NMR (101 MHz, $CDCl_3$) δ:** 137.8, 132.9, 130.1, 129.0, 128.2, 127.5, 126.9, 123.1, 18.8.

Minor Isomer (*Z*): ^{1}H NMR (400 MHz, $CDCl_3$) δ: 7.59 (dd, J = 8.0, 2.5 Hz, 1H), 7.33 – 7.27 (m, 2H),7.13 – 7.08 (m, 1H), 6.49 (dq, J = 11.4, 1.7 Hz, 1H), 5.90 (dq, J = 11.4, 7.1 Hz, 1H), 1.79 (dd, J = 7.1, 1.9 Hz, 3H). **^{13}C NMR (101 MHz, $CDCl_3$) δ:** 137.5, 132.7, 130.8, 129.5, 128.3, 128.2, 126.9, 124.2, 14.5.

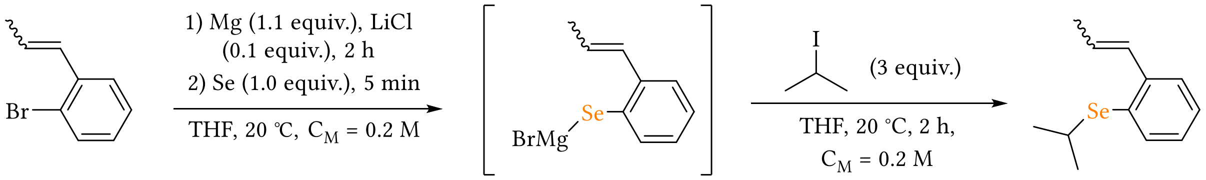

2 (200 mg, 1 mmol) — **not isolated** — **3** (74%, *E*/*Z* = 1.7:1)

Scheme S2. Synthesis of selenium-containing ligand **3** *via* selenium insertion with subsequent alkylation.

In an glovebox filled with argon, a 25 mL round-bottom flask was charged with Mg (finely ground, 26 mg, 1.1 mmol, 1.1 equiv.), lithium chloride (4.8 mg, 0.1 mmol, 0.1 equiv.) and THF (3 mL). To the resulting suspension, the solution of bromostyrene derivative **2** (200 mg, 1.0 mmol, 1.0 equiv.) in THF (2 mL) was added dropwise. The reaction was stirred at room temperature until the complete consumption of magnesium (leading to a clear colorless to pale yellow solution). The Grignard reagent was thus formed and gray Se (80 mg, 1.0 mmol, 1.0 equiv.) was added in one portion while stirring vigorously. The dark suspension of selenium in the reaction mixture turned rapidly (after 5 minutes) into a homogeneous clear orange solution indicating the full conversion of selenium. The flask was taken outside of the glovebox and 2-iodopropane (509 mg, 3 mmol, 3 equiv.) was added to the reaction mixture. After the addition of alkylating agent, a precipitate appears instantly leading to a grey to yellow turbid suspension. The reaction mixture was stirred at room temperature overnight and afterwards water was added (5 mL). The product was extracted with DCM (3 x 10 mL). The organic phases were combined, washed with brine and dried over anhydrous Na_2SO_4. The volatiles were removed under reduced pressure and the yellow oil was dried overnight under high vacuum ($1 \cdot 10^{-3}$ mbar). The product **3** was obtained as yellow oil (177.6 mg, 74%, *E*/*Z* = 1.7:1) and can be used without further purification.

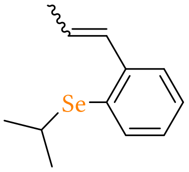

Major Isomer (*E*): ^{1}H NMR (400 MHz, $CDCl_3$) δ: 7.55 (dd, *J* = 7.7, 1.1 Hz, 1H), 7.47 (dd, *J* = 7.8, 1.2 Hz, 1H), 7.13 (m, 2H), 6.95 and 6.79 (dd, *J* = 15.6, 1.5 Hz, 1H), 6.22 – 6.04 (m, 1H), 3.41 (sept, *J* = 6.8 Hz, 1H), 1.91 (dd, *J* = 6.6, 1.7 Hz, 3H) 1.39 (d, *J* = 6.8 Hz, 6H). **^{13}C NMR (101 MHz, $CDCl_3$) δ:** 141.2, 135.8, 131.4, 127.9, 127.6, 127.15, 126.8, 33.9, 24.2, 18.8, 14.5, 14.4. **^{77}Se NMR (76 MHz, $CDCl_3$) δ:** 368.82.

Minor Isomer (*Z*): ^{1}H NMR (400 MHz, $CDCl_3$) δ: 7.55 (dd, *J* = 7.7, 1.1 Hz, 1H), 7.47 (dd, *J* = 7.8, 1.2 Hz, 1H), 7.26 – 7.01 (m, 2H), 6.63 and 6.56 (dd, *J* = 11.5, 1.6 Hz, 1H), 3.45 (sept, 1H), 1.77 (dd, *J* = 7.0, 1.8 Hz, 3H), 1.40 (d, *J* = 6.8 Hz, 6H). **^{13}C NMR (101 MHz, $CDCl_3$) δ:** 140.12, 134.04, 130.5, 127.94, 127.27, 127.1, 33.2, 24.1, 18.8, 14.5, 14.4. **^{77}Se NMR (76 MHz, $CDCl_3$) δ:** 379.53.

HRMS (ESI): calculated mass for $C_{12}H_{17}Se$ $[M+H]^+$: 241.0490; found: 241.0490(3).

Synthesis of the Ruthenium Complex

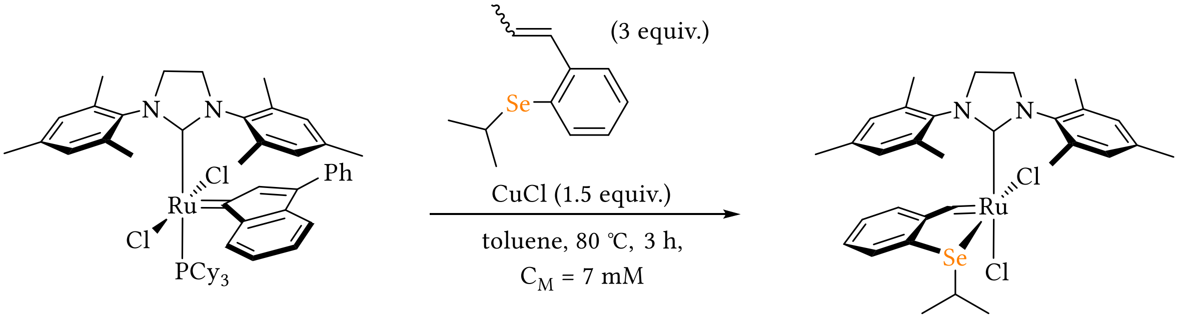

Ind-II
(100 mg, 0.105 mmol)

Ru8 (81%)

Scheme S3. Synthesis of selenium-containing ruthenium catalyst **Ru8**.

Dry Schlenk vessel was charged with ruthenium complex **Ind-II** (100 mg, 0.105 mmol, 1 equiv.) under argon atmosphere. Then, 15 mL of anhydrous toluene was added, resulting in a deep red solution. Selenium-containing styrene **3** (62 mg, 0.315 mmol, 3 equiv.) and anhydrous CuCl (15 mg, 0.158 mmol, 1.5 equiv.) were added to the reaction mixture and then it was stirred for 3 hours after heating up to 80 °C. Complete conversion of **Ind-II** catalyst was observed using TLC (stationary phase: SiO_2, eluent: EtOAc/*n*-hexane 10:90 v/v, $R_{F,\mathbf{Ind\text{-}II}}$ = 0.39, $R_{F,\mathbf{Ru8}}$ = 0) after that time. All the volatiles were removed under reduced pressure and the crude product was purified using column chromatography (stationary phase: SiO_2, eluent: EtOAc/*n*-hexane from 20:80 to 80:20 v/v). Product complex **Ru8** was obtained as a green solid (67 mg, 81%).

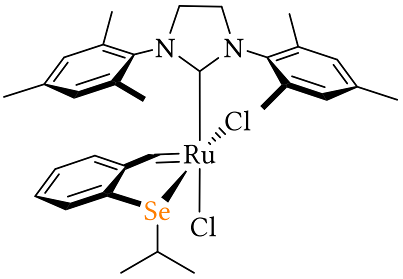

^{1}H NMR (400 MHz, CD_2Cl_2) δ: 17.47 (s, 1H), 7.52 – 7.41 (m, 2H), 7.20 (ddd, *J* = 7.7, 5.4, 3.0 Hz, 1H), 7.12 (s, 1H), 7.01 (s, 1H), 6.89 (s, 1H), 6.83 (d, *J* = 7.5 Hz, 1H), 5.94 (s, 1H), 4.25 – 3.72 (m, 5H), 2.67 (s, 1H), 2.53 (s, 1H), 2.47 (s, 1H), 2.36 (s, 1H), 2.15 (s, 1H), 1.54 (d, *J* = 4.4 Hz, 4H), 1.30 (t, *J* = 5.0 Hz, 2H), 1.02 (d, *J* = 6.8 Hz, 1H). **^{13}C NMR (101 MHz, CD_2Cl_2) δ:** δ 284.6, 215.0, 157.5, 140.3, 140.2, 138.4, 137.8, 136.8, 135.4, 132.4, 131.8, 130.7, 129.8, 129.7, 129.6, 129.2, 128.7, 124.9, 51.6, 51.3, 36.4, 23.7, 21.9, 21.1, 20.9, 20.3, 20.1, 18.7, 17.8. **^{77}Se NMR (76 MHz, CD_2Cl_2) δ**: 518.9.

X-Ray Data Collection and Structure Refinement

Good quality single-crystals of **Ru8** was selected for the X-ray diffraction experiments at T = 100(2) K. Diffraction data were collected on the Agilent Technologies SuperNova Dual Source diffractometer with Mo$K\alpha$ (λ = 0.71073) radiation using CrysAlis RED software.[1] The analytical numerical absorption correction using a multifaceted crystal model based on expressions derived by R.C. Clark & J.S. Reid,[2] implemented in SCALE3 ABSPACK scaling algorithm, were applied.[14] The structural determination procedure was carried out using the *SHELX* package.[3] The structures were solved with direct methods and then successive least-square refinement was carried out based on the full-matrix least-squares method on F^2 using the *SHELXL* program.[3] All H-atoms were positioned geometrically with C–H equal to 0.93, 0.96, 0.97 and 0.98 Å for the aromatic, methyl, methylene and methine H-atoms, respectively. The H-atoms were constrained to ride on their parent atoms with U_{iso}(H) = xU_{eq}(C), where x = 1.2 for the aromatic, methylene and methine H-atoms, and 1.5 for the methyl H-atoms, respectively. Additionally, several distinct peaks on the difference Fourier map of **Ru8** were indicating the presence of a disordered solvent molecule/s. However, all of the attempts to propose reasonable model of disorder have failed. Therefore, the solvent contribution was removed by applying the appropriate MASK procedure in the Olex2 program.[4] Calculated total solvent accessible volume / cell was 919.0 Å^3 occupied by 184.8 electrons per unit cell. The figures for this publication were prepared using *Olex2* and ORTEP-3 programs.[17-5]

---

1 *CrysAlis CCD and CrysAlis RE*D; Oxford Diffraction Ltd: Yarnton, **2008.**
2 R. C. Clark, J. S. Reid. *Acta Cryst. Sect. A* **1994**, 51, 887.
3 G. M. Sheldrick, *Acta Crystallogr. Sect. A* **2008**, 64, 112.
4 O. V. Dolomanov, L. J. Bourhis, R. J. Gildea, J. A. K. Howard, H. Puschmann, *J. Appl. Cryst.* **2009**, 42, 339.
5 L. J. Farrugia, *J. Appl. Cryst.* **2012**, 45, 849.

Table S1. Crystal data and structure refinement details for **Ru8**.

Compound	Ru8
Empirical formula	$C_{31}H_{38}Cl_2N_2RuSe$
Formula weight	689.56
Temperature/K	100(2)
Crystal system	Monoclinic
Space group	I2/a
a/Å	16.6540(2)
b/Å	16.4089(2)
c/Å	26.2915(3)
α/°	90
β/°	95.3149(11)
γ/°	90
Volume/Å^3	7153.86(15)
Z	8
ρ_{calc}g/cm^3	1.280
μ/mm^{-1}	1.624
F(000)	2800.0
Crystal size/mm^3	0.33 × 0.18 × 0.13
Radiation	MoKα (λ = 0.71073)
2Θ range for data collection/°	4.524 to 52.742
Index ranges	-20 ≤ h ≤ 20,
Reflections collected	-20 ≤ k ≤ 20,
Independent reflections	-32 ≤ l ≤ 32
Data/restraints/parameters	47448
Goodness-of-fit on F^2	7316 [R_{int} = 0.0267,
Final R indexes [I>=2σ (I)]	R_{sigma} = 0.0175]
Final R indexes [all data]	7316/0/342
Largest diff. peak/hole / e Å^{-3}	1.35/-1.30

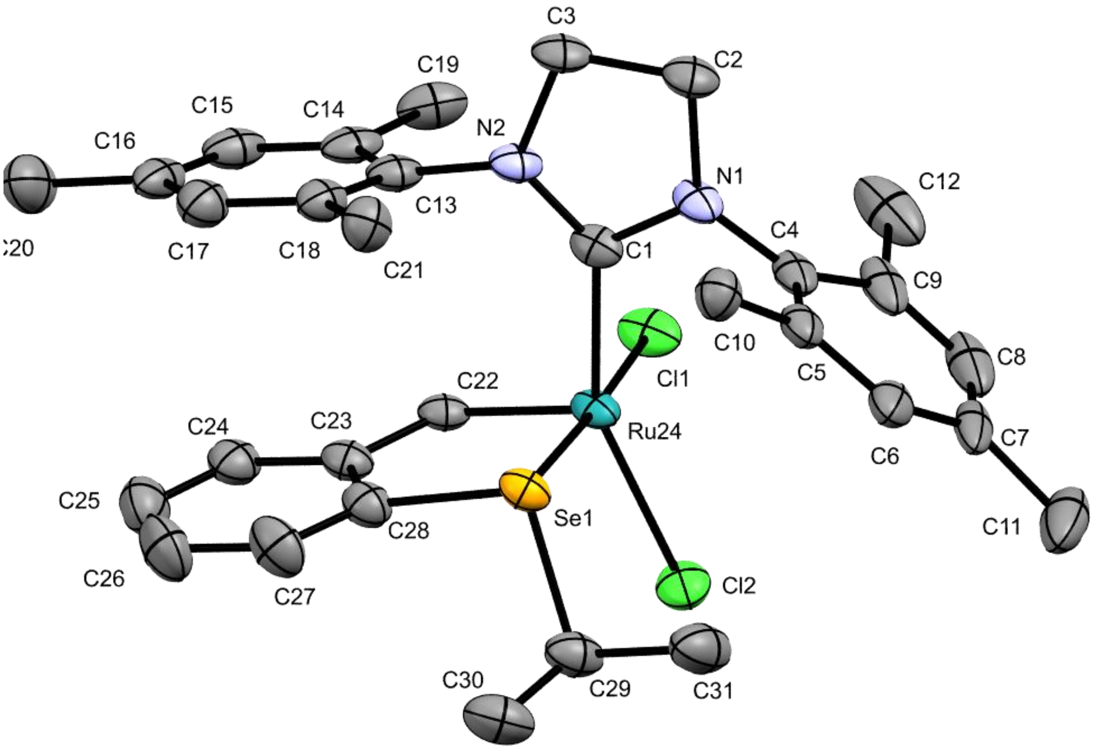

Figure S1. ORTEP diagram of **Ru8** with 50% probability ellipsoids; hydrogen atoms were omitted for clarity.

Table S2. Bond lengths for **Ru8.**

Atom	Atom	Length/Å	Atom	Atom	Length/Å
C1	N1	1.345(4)	C15	C16	1.379(5)
C1	N2	1.339(4)	C16	C17	1.403(5)
C1	Ru24	2.022(3)	C16	C20	1.513(5)
C2	C3	1.524(5)	C17	C18	1.397(5)
C2	N1	1.478(4)	C18	C21	1.515(5)
C3	N2	1.481(4)	C22	C23	1.459(5)
C4	C5	1.401(5)	C22	Ru24	1.825(3)
C4	C9	1.405(5)	C23	C24	1.404(5)
C4	N1	1.425(4)	C23	C28	1.391(4)
C5	C6	1.384(5)	C24	C25	1.381(6)
C5	C10	1.508(5)	C25	C26	1.390(6)
C6	C7	1.404(6)	C26	C27	1.390(7)
C7	C8	1.378(7)	C27	C28	1.387(5)
C7	C11	1.524(7)	C28	Se1	1.927(3)
C8	C9	1.367(7)	C29	C30	1.500(5)
C9	C12	1.513(6)	C29	C31	1.528(6)
C13	C14	1.408(4)	C29	Se1	1.991(4)
C13	C18	1.396(5)	Cl1	Ru24	2.3821(9)
C13	N2	1.429(4)	Cl2	Ru24	2.374(1)
C14	C15	1.373(5)	Ru24	Se1	2.4479(7)
C14	C19	1.508(5)			

Table S3. Values of valence angles for **Ru8**.

Atom	Atom	Atom	Angle/°	Atom	Atom	Atom	Angle/°
N1	C1	N2	108.4(3)	C22	C23	C24	122.1(3)
N1	C1	Ru24	118.4(2)	C22	C23	C28	119.2(3)
N2	C1	Ru24	133.0(2)	C24	C23	C28	118.7(3)
C2	C3	N2	103.2(3)	C23	C24	C25	120.1(3)
C5	C4	C9	121.2(3)	C24	C25	C26	120.5(4)
C5	C4	N1	118.5(3)	C25	C26	C27	120.2(4)
C9	C4	N1	120.3(3)	C26	C27	C28	119.1(4)
C4	C5	C6	117.7(3)	C23	C28	C27	121.4(3)
C4	C5	C10	120.9(3)	C23	C28	Se1	113.9(2)
C6	C5	C10	121.3(3)	C27	C28	Se1	124.5(3)
C5	C6	C7	121.3(4)	C30	C29	C31	112.2(3)
C6	C7	C8	118.8(4)	C30	C29	Se1	111.6(2)
C6	C7	C11	119.0(4)	C31	C29	Se1	104.9(3)
C8	C7	C11	122.2(4)	C1	N1	C2	113.3(3)
C7	C8	C9	121.9(4)	C1	N1	C4	121.7(3)
C4	C9	C8	118.4(4)	C2	N1	C4	124.3(3)
C4	C9	C12	121.5(4)	C1	N2	C3	112.6(3)

C8	C9	C12	120.0(4)	C1	N2	C13	128.2(3)
C14	C13	C18	122.2(3)	C3	N2	C13	118.8(3)
C14	C13	N2	118.7(3)	C1	Ru24	C22	97.4(1)
C18	C13	N2	118.9(3)	C1	Ru24	Cl1	88.82(9)
C13	C14	C15	117.9(3)	C1	Ru24	Cl2	154.20(9)
C13	C14	C19	120.8(3)	C1	Ru24	Se1	92.34(9)
C15	C14	C19	121.2(3)	C22	Ru24	Cl1	90.6(1)
C14	C15	C16	122.1(3)	C22	Ru24	Cl2	108.1(1)
C15	C16	C17	119.3(3)	C22	Ru24	Se1	85.1(1)
C15	C16	C20	121.8(3)	Cl1	Ru24	Cl2	87.12(3)
C17	C16	C20	118.9(3)	Cl1	Ru24	Se1	175.62(3)
C16	C17	C18	120.9(3)	Cl2	Ru24	Se1	93.63(3)
C13	C18	C17	117.6(3)	C28	Se1	C29	102.5(1)
C13	C18	C21	122.5(3)	C28	Se1	Ru24	94.8(1)
C17	C18	C21	119.9(3)	C29	Se1	Ru24	110.2(1)
C23	C22	Ru24	126.8(2)				

Table S4. Values of torsion angles for **Ru8**.

A	B	C	D	Angle/°	A	B	C	D	Angle/°
N2	C1	N1	C2	-2.9(4)	N2	C13	C18	C17	176.7(3)
N2	C1	N1	C4	167.5(3)	N2	C13	C18	C21	-2.2(5)
Ru24	C1	N1	C2	171.9(2)	C14	C13	N2	C1	-95.0(4)
Ru24	C1	N1	C4	-17.7(4)	C14	C13	N2	C3	92.6(4)
N1	C1	N2	C3	-1.9(4)	C18	C13	N2	C1	90.4(4)
N1	C1	N2	C13	-174.7(3)	C18	C13	N2	C3	-82.0(4)
Ru24	C1	N2	C3	-175.7(2)	C13	C14	C15	C16	0.1(5)
Ru24	C1	N2	C13	11.5(5)	C19	C14	C15	C16	-176.6(3)
N1	C1	Ru24	C22	-175.0(2)	C14	C15	C16	C17	1.4(5)
N1	C1	Ru24	Cl1	-84.5(2)	C14	C15	C16	C20	-179.0(3)
N1	C1	Ru24	Cl2	-3.6(4)	C15	C16	C17	C18	-1.0(5)
N1	C1	Ru24	Se1	99.7(2)	C20	C16	C17	C18	179.4(3)
N2	C1	Ru24	C22	-1.7(3)	C16	C17	C18	C13	-0.8(5)
N2	C1	Ru24	Cl1	88.8(3)	C16	C17	C18	C21	178.1(3)
N2	C1	Ru24	Cl2	169.7(2)	Ru24	C22	C23	C24	-177.7(3)
N2	C1	Ru24	Se1	-87.0(3)	Ru24	C22	C23	C28	3.1(5)
N1	C2	C3	N2	-6.5(3)	C23	C22	Ru24	C1	-96.5(3)
C3	C2	N1	C1	6.2(3)	C23	C22	Ru24	Cl1	174.6(3)
C3	C2	N1	C4	-163.9(3)	C23	C22	Ru24	Cl2	87.4(3)
C2	C3	N2	C1	5.6(3)	C23	C22	Ru24	Se1	-4.8(3)
C2	C3	N2	C13	179.2(3)	C22	C23	C24	C25	-178.8(3)
C9	C4	C5	C6	-8.8(5)	C28	C23	C24	C25	0.4(5)
C9	C4	C5	C10	168.0(3)	C22	C23	C28	C27	177.4(3)
N1	C4	C5	C6	174.2(3)	C22	C23	C28	Se1	1.8(4)
N1	C4	C5	C10	-9.0(5)	C24	C23	C28	C27	-1.9(5)

C5	C4	C9	C8	9.5(6)	C24	C23	C28	Se1	-177.4(2)
C5	C4	C9	C12	-167.4(4)	C23	C24	C25	C26	0.5(6)
N1	C4	C9	C8	-173.6(4)	C24	C25	C26	C27	-0.1(7)
N1	C4	C9	C12	9.5(6)	C25	C26	C27	C28	-1.2(6)
C5	C4	N1	C1	-83.2(4)	C26	C27	C28	C23	2.3(6)
C5	C4	N1	C2	86.1(4)	C26	C27	C28	Se1	177.4(3)
C9	C4	N1	C1	99.7(4)	C23	C28	Se1	C29	-116.0(3)
C9	C4	N1	C2	-91.0(4)	C23	C28	Se1	Ru24	-4.0(2)
C4	C5	C6	H6	-178.7	C27	C28	Se1	C29	68.6(3)
C4	C5	C6	C7	1.4(6)	C27	C28	Se1	Ru24	-179.5(3)
C10	C5	C6	C7	-175.4(4)	C30	C29	Se1	C28	-58.4(3)
C5	C6	C7	C8	5.3(7)	C30	C29	Se1	Ru24	-158.4(2)
C5	C6	C7	C11	-175.6(4)	C31	C29	Se1	C28	179.9(3)
C6	C7	C8	C9	-4.7(7)	C31	C29	Se1	Ru24	79.9(3)
C11	C7	C8	C9	176.2(4)	C1	Ru24	Se1	C28	101.4(1)
C7	C8	C9	C4	-2.5(7)	C1	Ru24	Se1	C29	-153.4(1)
C7	C8	C9	C12	174.4(4)	C22	Ru24	Se1	C28	4.2(1)
C18	C13	C14	C15	-2.1(5)	C22	Ru24	Se1	C29	109.3(1)
C18	C13	C14	C19	174.7(3)	Cl1	Ru24	Se1	C28	-4.0(4)
N2	C13	C14	C15	-176.4(3)	Cl1	Ru24	Se1	C29	101.2(4)
N2	C13	C14	C19	0.4(5)	Cl2	Ru24	Se1	C28	-103.7(1)
C14	C13	C18	C17	2.4(5)	Cl2	Ru24	Se1	C29	1.5(1)
C14	C13	C18	C21	-176.5(3)					

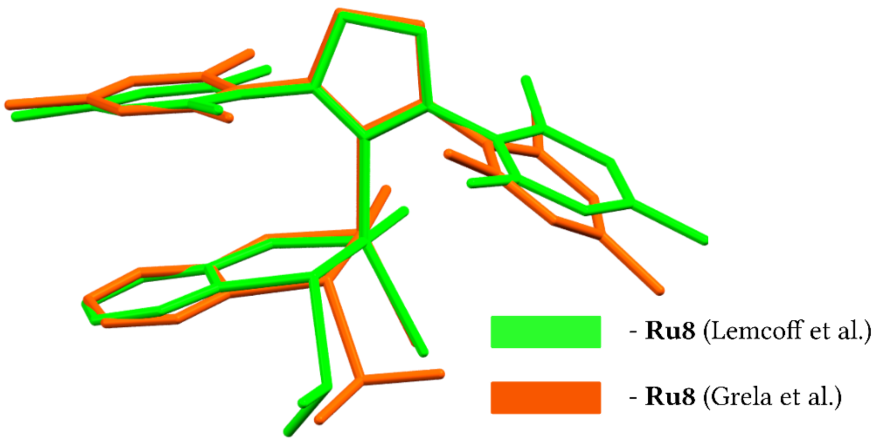

Figure S2. Superimposed molecules of **Ru8** obtained by us and **Ru8** (CCDC 790618) obtained by the group of Lemcoff.[6] The hydrogen atoms are omitted for clarity.

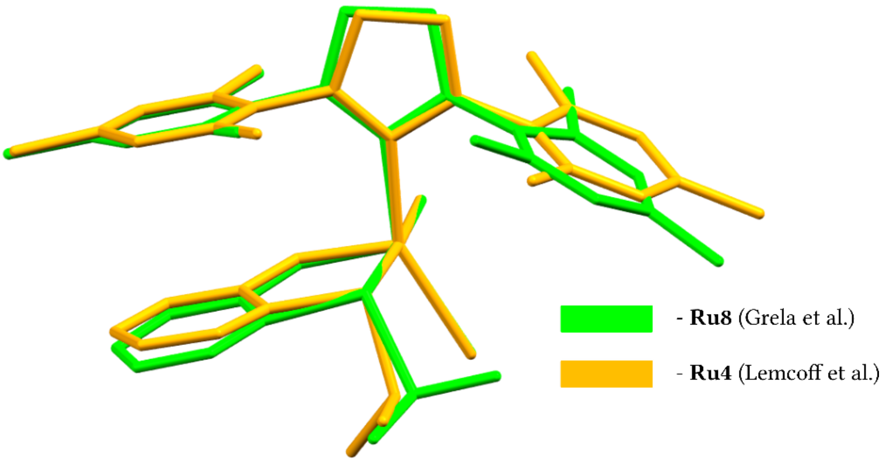

Figure S3. Superimposed molecules of **Ru8** obtained by us and **Ru4** (CCDC 689901) obtained by the group of Lemcoff.[7] The hydrogen atoms are omitted for clarity.

---

[6] C. S. Diesendruck, E. Tzur, A. Ben-Asuly, I. Goldberg, B. F. Straub, N. G. Lemcoff, *Inorg. Chem.* **2009**, 48, 10819.

[7] A. Ben-Asuly, E. Tzur, C. E. Diesendruck, M. Sigalov, I. Goldberg, N. G. Lemcoff, *Organometallics* **2008**, 27, 811.

Activation of Ru8 and Catalytic Activity

Demonstration of Ru8 Latency

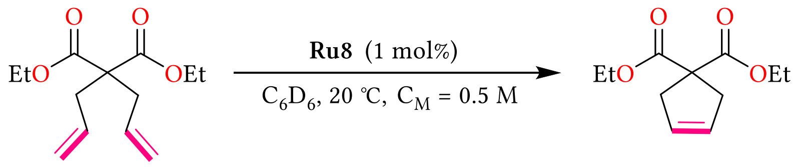

4
(48.5 mg, 0.2 mmol)

5
(<5% conv.)

Scheme S4. RCM reaction of **4** as a method for Ru catalysts activity assessment.

Procedure: In a glovebox filled with argon, diene **4** (48.5 mg, 50 µL, 0.2 mmol, 1 equiv.) was added to a solution of **Ru8** (1.38 mg, 0.002 mmol, 1 mol%) in C_6D_6 (0.4 mL) in a screw cap NMR tube. The NMR tube was taken outside of the glovebox and the reaction mixture was stirred at 20 °C for the required time. Data was recorded using 32 scans with a D1 delay time minimum of 1 s between each pulse. Progress of the reaction was monitored through the disappearance of the methylene signals of **4** (2.63 ppm) and the growth of the methylene proton signal of the product **5** (3.01 ppm). After the time of 1 month, the conversion of the diene **4** to the corresponding cycloalkene derivative **5** was not observable.

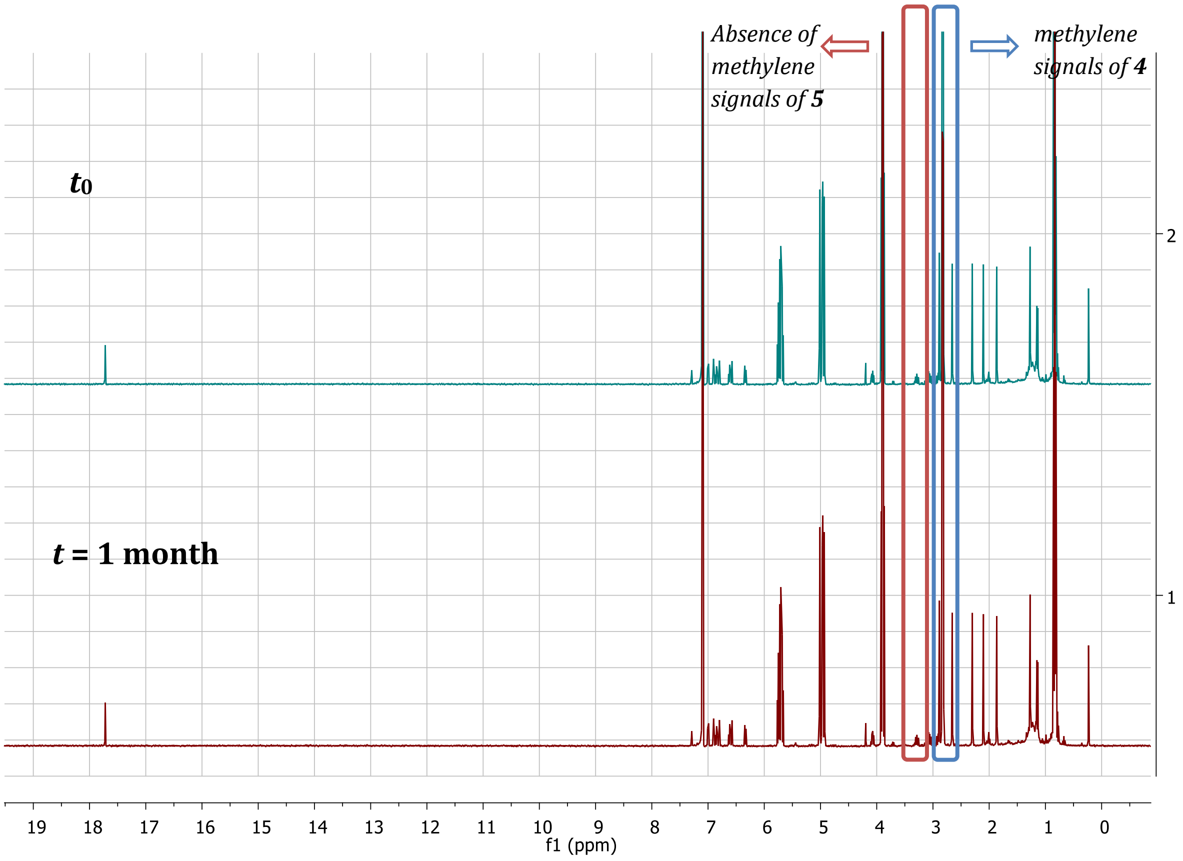

Figure S4. ^{1}H NMR spectra of reaction mixture at t_0 (top) and after the time of one month (bottom) in benzene.

Activation of Ru8 in CD_2Cl_2 with UV-Irradiation

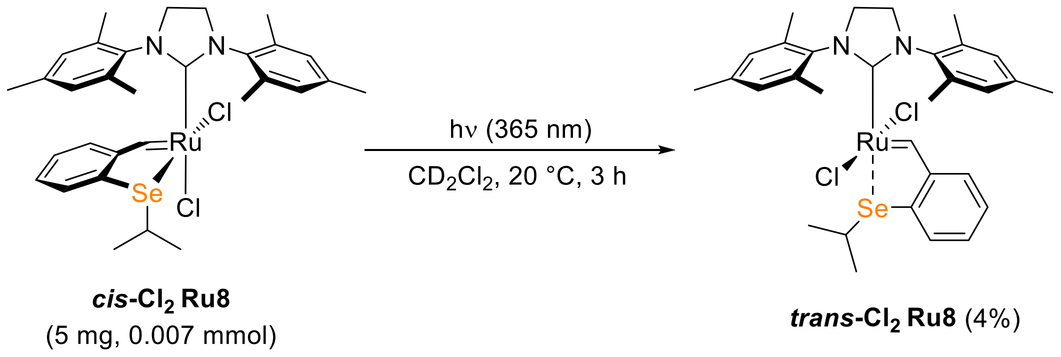

Scheme S5. *Cis*-*trans* isomerization of **Ru8** in DCM with UV-irradiation.

In a glovebox filled with argon, a screw cap NMR tube was charged with **Ru8** (5 mg, 0.0072 mmol) in CD_2Cl_2 (0.5 mL). The NMR tube was taken outside of the glovebox and placed in a UV-reactor. Data was recorded using 32 scans with a D1 delay time minimum of 1 s between each pulse. Progress of the reaction was monitored through the disappearance of the benzylidene signal of *cis*-Cl_2 **Ru8** (17.46 ppm) and the growth of the benzylidene signal of *trans*-Cl_2 **Ru8** (17.72 ppm).

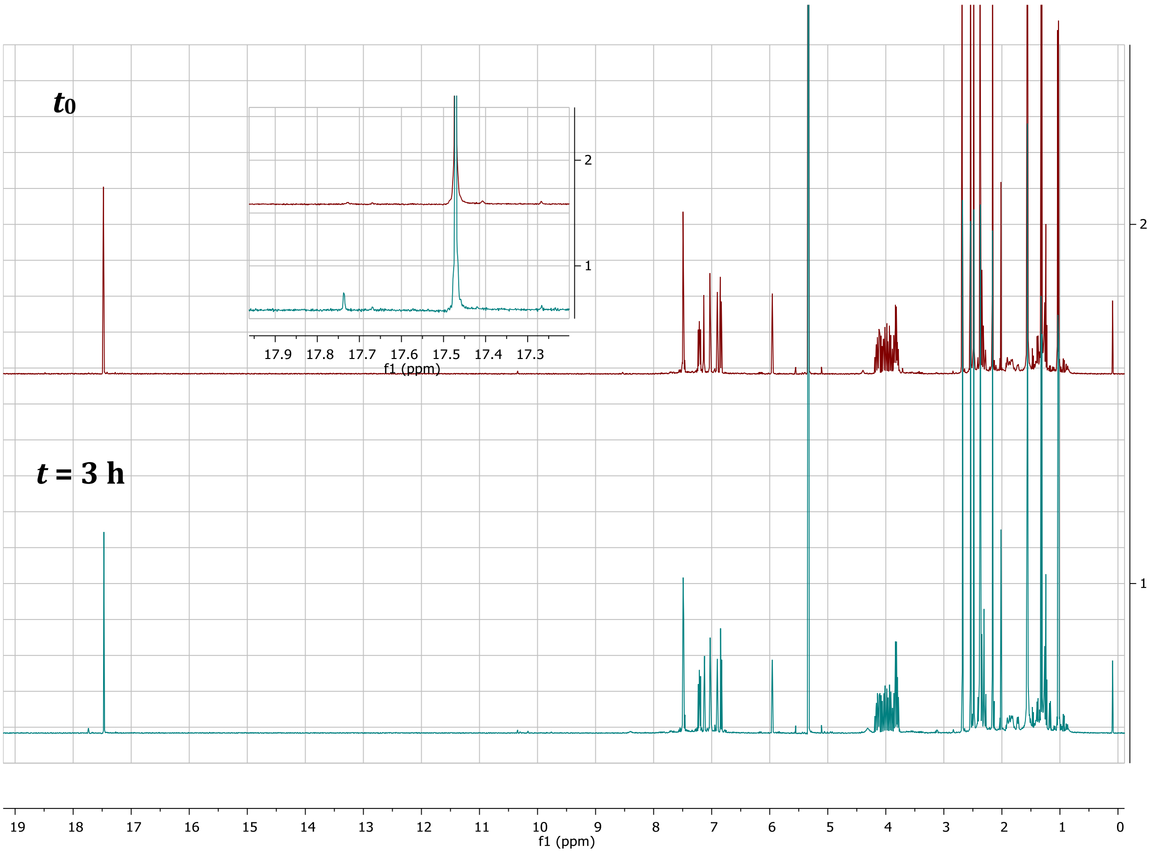

Figure S5. ^{1}H NMR spectra of **Ru8** in CD_2Cl_2 at t_0 (top) and after 3 h of UV-irradiation (bottom).

Activation of Ru8 in C_6D_6 with UV-Irradiation

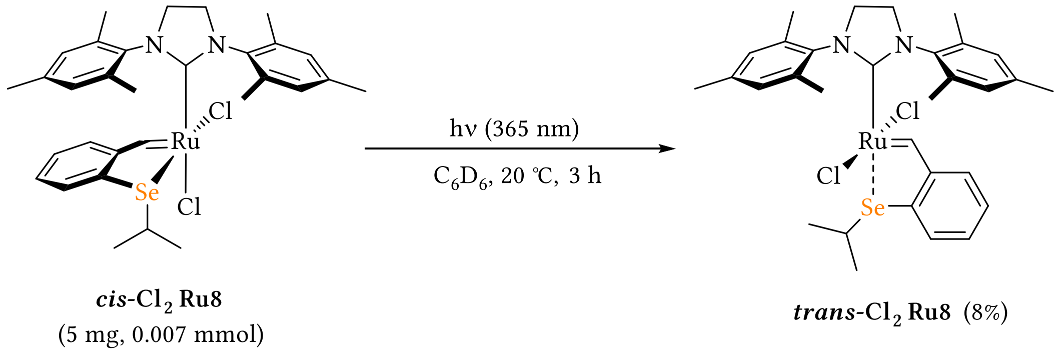

Scheme S6. *Cis*-*trans* isomerization of **Ru8** in benzene-d_6 with UV-irradiation.

In a glovebox filled with argon, a screw cap NMR tube was charged with **Ru8** (5 mg, 0.0072 mmol) in C_6D_6 (0.5 mL). The NMR tube was taken outside of the glovebox and placed in a UV-reactor. Data was recorded using 32 scans with a D1 delay time minimum of 1 s between each pulse. Progress of the reaction was monitored through the disappearance of the benzylidene signal of *cis*-Cl_2 **Ru8** (17.46 ppm) and the growth of the benzylidene signal of *trans*-Cl_2 **Ru8** (17.72 ppm).

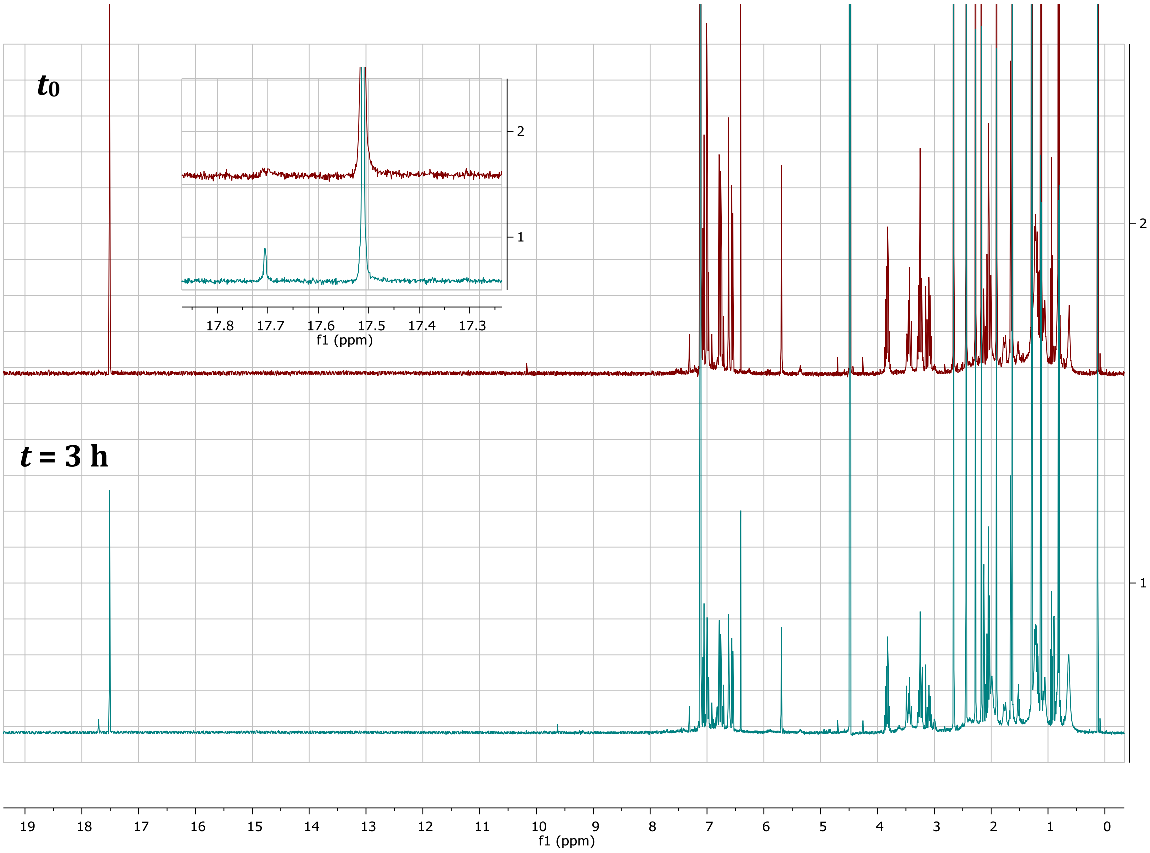

Figure S6. ^{1}H NMR spectra of **Ru8** in C_6D_6 at t_0 (top) and after 3 h of UV-irradiation (bottom).

Activity of Ru8 in RCM Reactions with Thermal Activation in C_6D_6

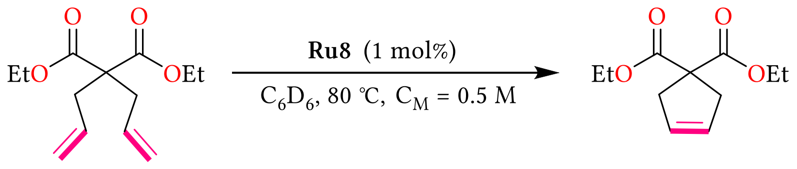

4
(48.5 mg, 0.2 mmol)

5
(29% conv. after 7 h,
63% conv. after 24 h)

Scheme S7. RCM reaction of **4** promoted by **Ru8** complex at increased temperature.

Procedure: In a glovebox filled with argon, diene **4** (48.5 mg, 50 µL, 0.2 mmol, 1 equiv.) was added to a solution of **Ru8** (1.38 mg, 0.002 mmol, 0.1 mol%) in C_6D_6 (0.4 mL) in a screw cap NMR tube. The NMR tube was taken outside of the glovebox and the reaction mixture was heated in an oil bath at 80 °C for the required time. Data was recorded using 32 scans with a D1 delay time minimum of 5 s between each pulse. Progress of the reaction was monitored through the disappearance of the methylene signals of **4** (2.63 ppm) and the growth of the methylene proton signal of the product **5** (3.01 ppm).

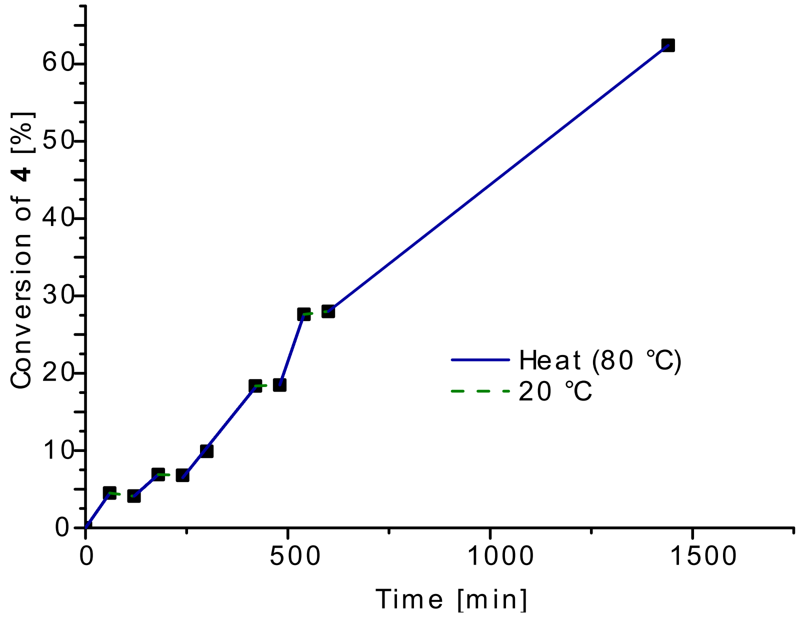

Figure S7. Progress of the RCM reaction of **4** in benzene-d_6 at 80 °C monitored by ^1H NMR.

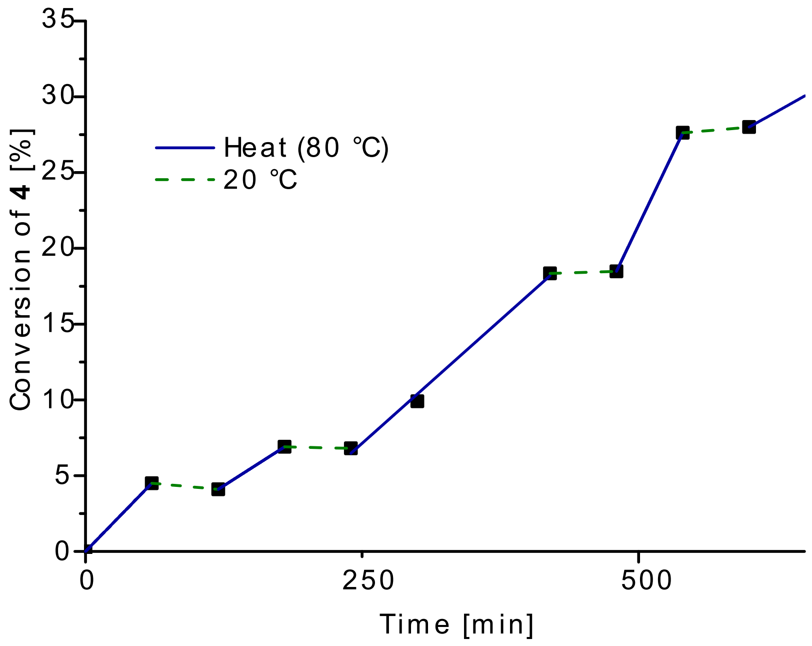

Figure S8. Progress of the RCM reaction of **4** in benzene-d_6 at 80 °C monitored by ^{1}H NMR.

Activity of Ru8 in RCM Reactions with Thermal Activation in $C_6D_5CD_3$

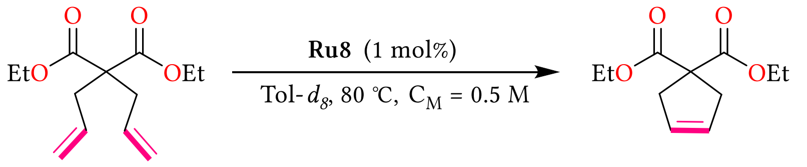

Scheme S8. RCM reaction of **4** promoted by **Ru8** complex at increased temperature.

Procedure: In a glovebox filled with argon, diene **4** (48.5 mg, 50 μL, 0.2 mmol, 1 equiv.) was added to a solution of **Ru8** (1.38 mg, 0.002 mmol, 0.1 mol%) in deuterated toluene (0.4 mL) in a screw cap NMR tube. The NMR tube was taken outside of the glovebox and the reaction mixture was heated in an oil bath at 80 °C for the required time. Data was recorded using 32 scans with a D1 delay time minimum of 5 s between each pulse. Progress of the reaction was monitored through the disappearance of the methylene signals of **4** (2.63 ppm) and the growth of the methylene proton signal of the product **5** (3.01 ppm).

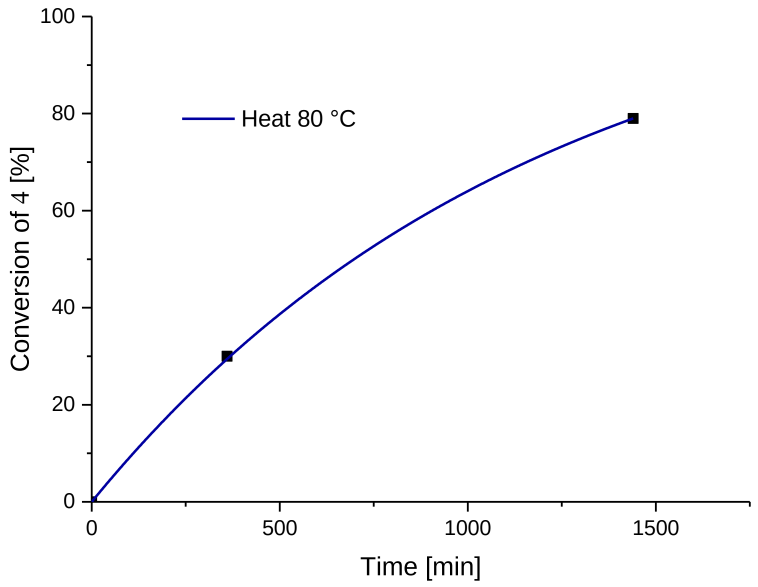

Figure S9. Progress of the RCM reaction of **4** promoted by **Ru8** in deuterated toluene at 80 °C monitored by ^{1}H NMR.

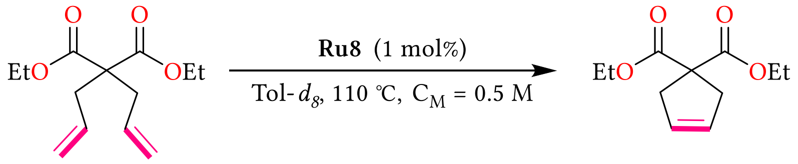

4
(48.5 mg, 0.2 mmol)

5
78% conv. after 5 h

Scheme S9. RCM reaction of **4** promoted by **Ru8** complex at increased temperature.

Procedure: In a glovebox filled with argon, diene **4** (48.5 mg, 50 µL, 0.2 mmol, 1 equiv.) was added to a solution of **Ru8** (1.38 mg, 0.002 mmol, 0.1 mol%) in deuterated toluene (0.4 mL) in a screw cap NMR tube. The NMR tube was taken outside of the glovebox and the reaction mixture was heated in an oil bath at 110 °C for the required time. Data was recorded using 32 scans with a D1 delay time minimum of 5 s between each pulse. Progress of the reaction was monitored through the disappearance of the methylene signals of **4** (2.63 ppm) and the growth of the methylene proton signal of the product **5** (3.01 ppm).

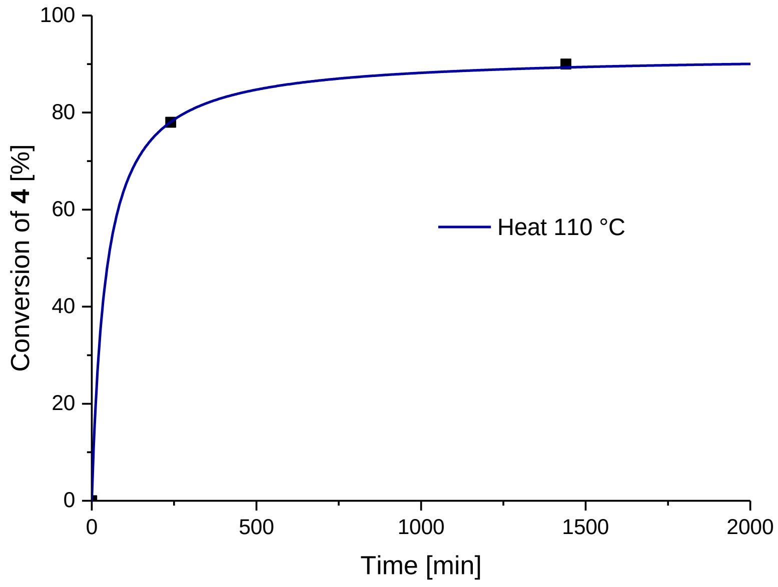

Figure S10. Progress of the RCM reaction of **4** promoted by **Ru8** in deuterated toluene at 110 °C monitored by ^{1}H NMR.

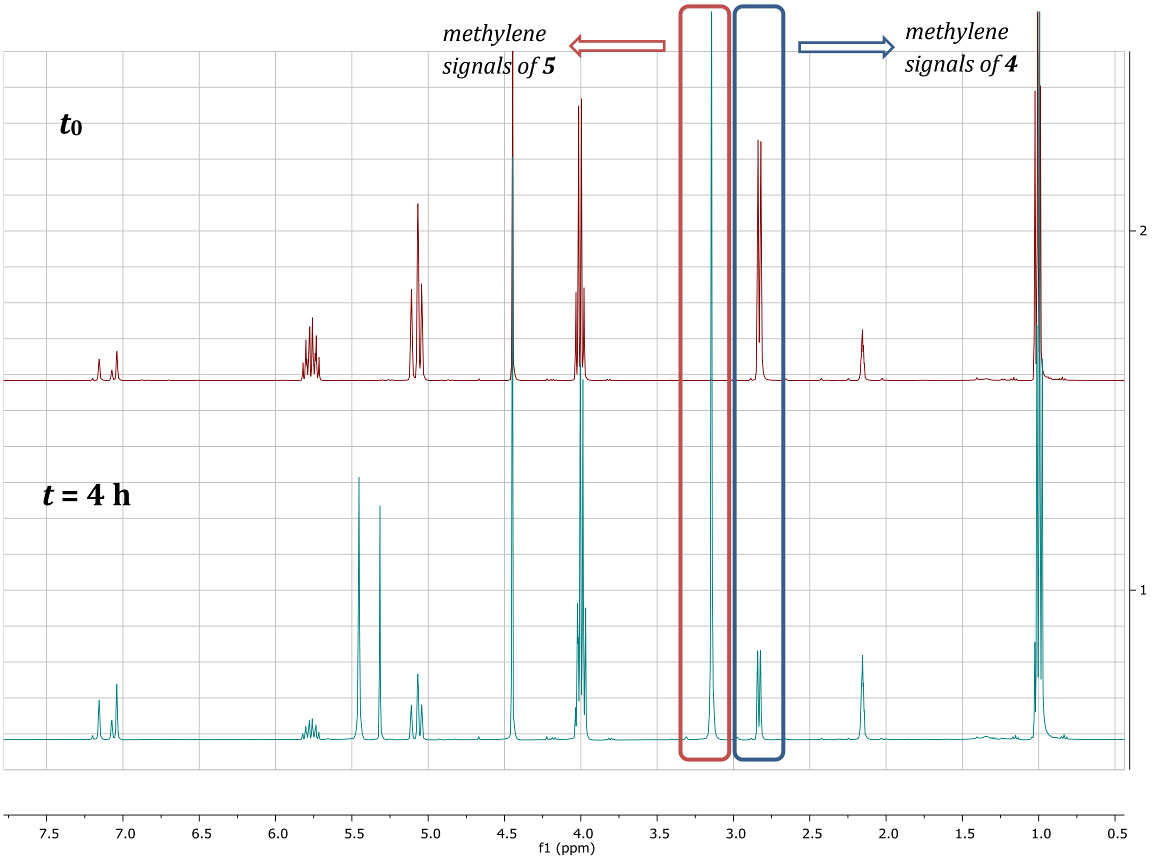

Figure S11. ^1H NMR spectra of a crude mixture of **4** in presence of **Ru8** in toluene at 110 °C at t=0 (top) and t= 4 h (bottom).

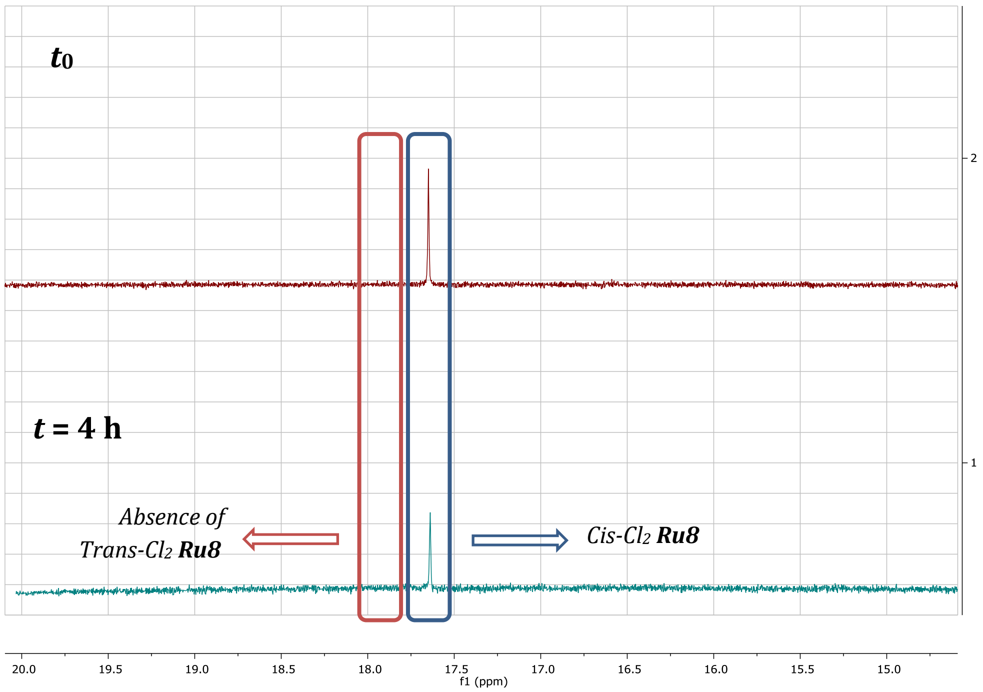

Figure S12. Partial ^1H NMR spectra (benzylidene region) of a crude mixture of **4** in presence of **Ru8** in toluene at 110 °C at t=0 (top) and t= 4 h (bottom).

Activity of Ru8 in RCM Reactions with Photochemical Activation in DCM

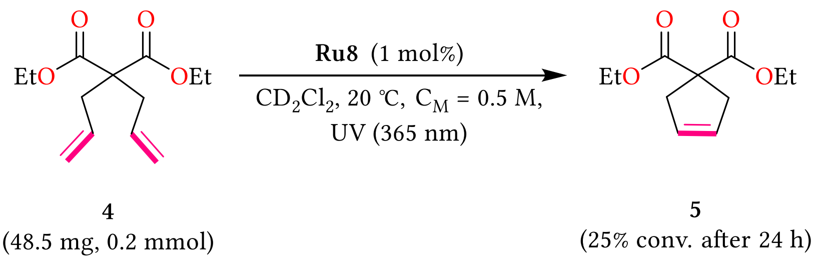

Scheme S10. RCM reaction of **4** promoted by **Ru8** complex under irradiation.

Procedure: In a glovebox filled with argon, diene **4** (48.5 mg, 50 µL, 0.2 mmol, 1 equiv.) was added to a solution of **Ru8** (1.38 mg, 0.002 mmol, 0.1 mol%) in CD_2Cl_2 (0.4 mL) in a screw cap NMR tube. The NMR tube was taken outside of the glovebox and placed in the UV photoreactor (λ = 365 nm) for a required time. Data was recorded using 32 scans with a D1 delay time minimum of 1 s between each pulse. Progress of the reaction was monitored through the disappearance of the methylene signals of **4** (2.63 ppm) and the growth of the methylene proton signal of the product **5** (3.01 ppm).

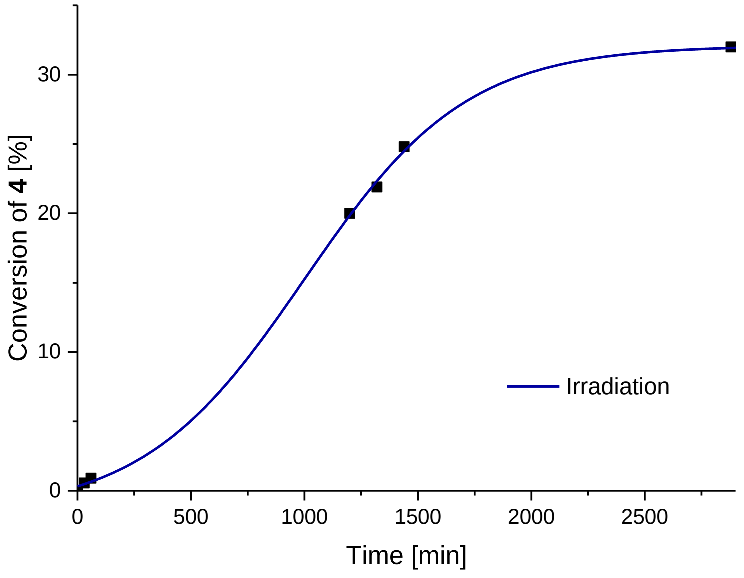

Figure S13. Progress of the RCM reaction of **4** promoted by **Ru8** in DCM under UV irradiation (365 nm) monitored by ^{1}H NMR.

Activity of Ru8 in RCM Reactions with Photochemical Activation in Benzene

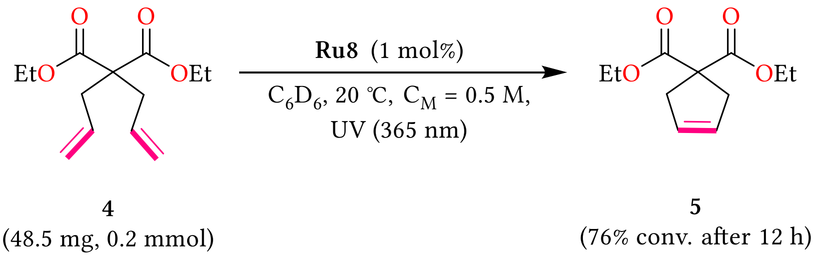

Scheme S11. RCM reaction of **4** promoted by **Ru8** complex under irradiation.

Procedure: In a glovebox filled with argon, diene **4** (48.5 mg, 50 µL, 0.2 mmol, 1 equiv.) was added to a solution of **Ru8** (1.38 mg, 0.002 mmol, 0.1 mol%) in C_6D_6 (0.4 mL) in a screw cap NMR tube. The NMR tube was taken outside of the glovebox and placed in the UV photoreactor (λ = 365 nm) for a required time. Data was recorded using 32 scans with a D1 delay time minimum of 1 s between each pulse. Progress of the reaction was monitored through the disappearance of the methylene signals of **4** (2.63 ppm) and the growth of the methylene proton signal of the product **5** (3.01 ppm).

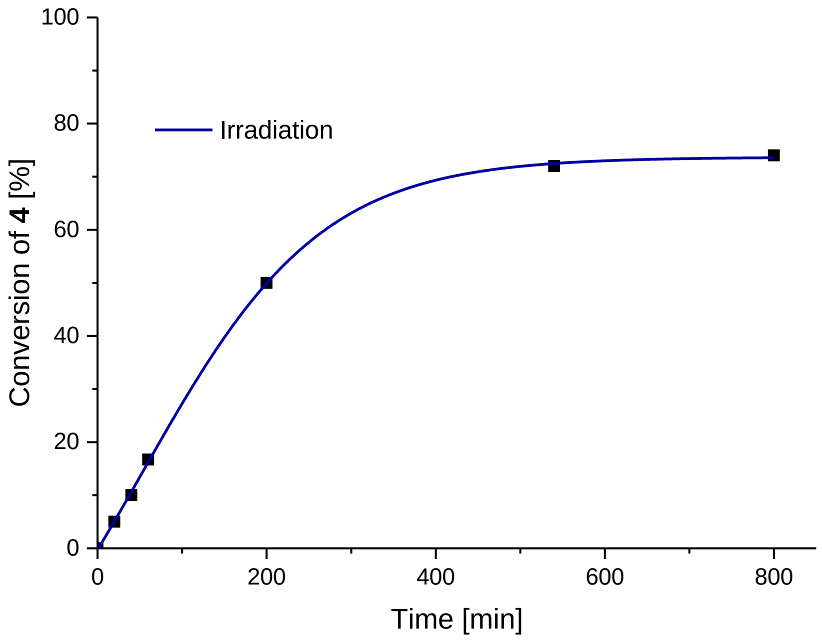

Figure S14. Progress of the RCM reaction of **4** promoted by **Ru8** in benzene-d_6 under UV irradiation (365 nm) monitored by ^{1}H NMR.

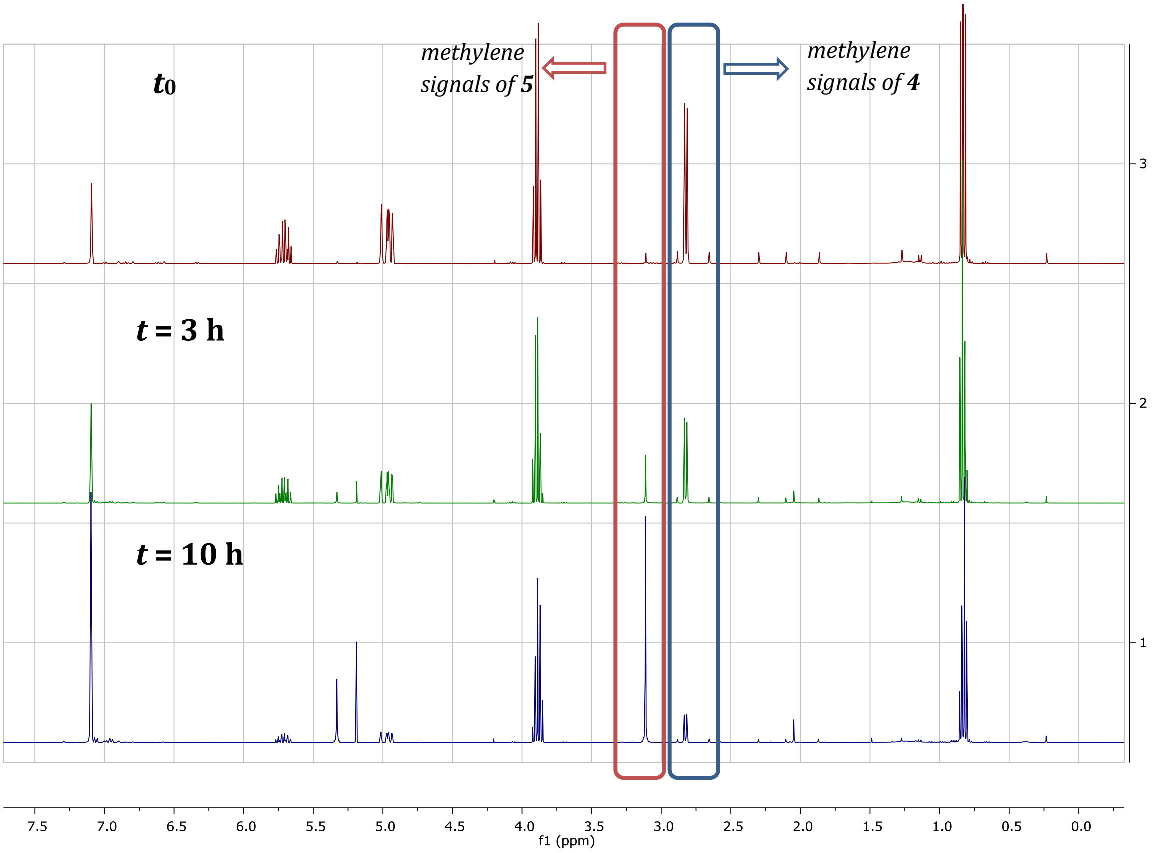

Figure S15. ^{1}H NMR spectra of a crude mixture of **4** in presence of **Ru8** in benzene under light irradiation at t=0 (top), t=3 h (middle) and t= 7 h bottom

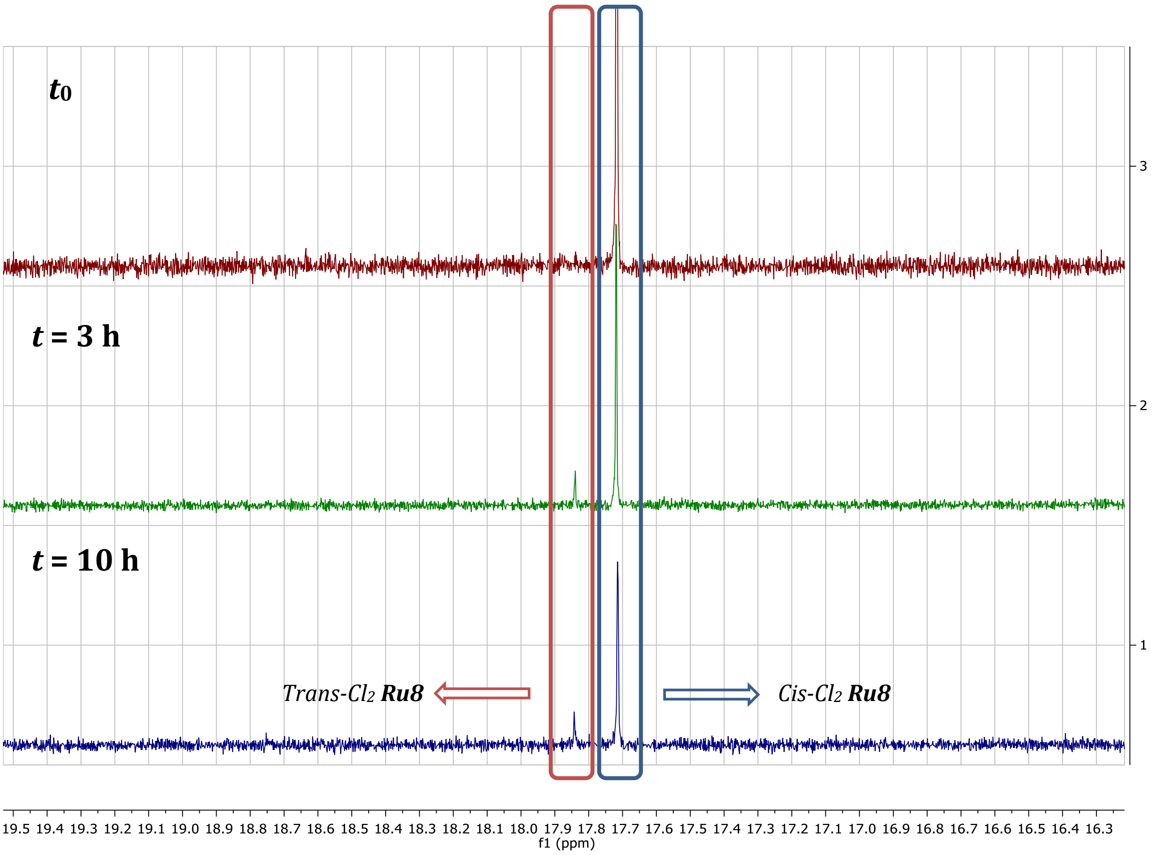

Figure S16. Partial^{1}H NMR spectra of a crude mixture of **4** in presence of **Ru8** in benzene under light irradiation at t=0 (top), t=3 h (middle) and t= 10 h bottom.

Polymerization Reactions Catalyzed by Ru8

Polymerization of Cyclooctene (COE)

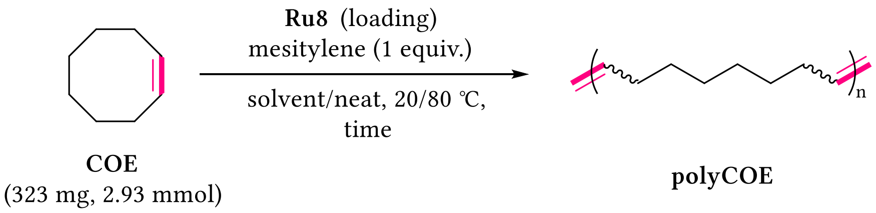

Scheme S12. ROMP polymerization of **COE** promoted by **Ru8** catalyst.

15 mL vial equipped with a screw cap was charged with substrate **COE** (323 mg, 385 µL, 2.93 mmol, 1 equiv.). Unless the reaction was carried out in neat, an appropriate solvent (6 mL) was added (DCM or toluene). To the resulting solution mesitylene (353 mg, 407 µL, 2.93 mmol, 1 equiv.) was added as internal standard followed by the addition of **Ru8** catalyst stock solution in DCM (1 M, 0.3 mL, 0.303 mg, 0.44 µmol, 150 ppm, 0.0150 mol%). For the reactions carried out in neat conditions, the solvent (DCM) was removed under the reduced pressure before photochemical activation. The vial was then placed in the UV-reactor (365 nm) or in heated oil bath (80 °C) and was left for a required time affording a highly viscous solution. NMR of this crude solution was recorded and highlighted the presence of the polymer. The resulting polymer was precipitated out with acetone and methanol then filtered off. In the case of reaction in neat, the solid was washed with hexane and DCM and filtered. The filtrate was analyzed by ^{1}H NMR to assess the conversion of the reaction.

In case of reaction with 10 ppm of **Ru8**, the following conditions has been used:
To a 15 mL screw capped vial containing **COE** (840 mg, 1000 µL, 7.62 mmol, 1 equiv.), mesitylene (916 mg, 1056 µL, 7.62 mmol, 1 equiv.) was added as internal standard followed by the addition of **Ru8** catalyst stock solution in DCM (1 mg/mL, 0.050 mL, 0.05 mg, 0.076 µmol, 10 ppm, 0.0010 mol%).

Table S5. Results of ROMP reactions involving **COE** as a substrate.

Entry	Solvent	Ru8 [ppm]	Activa-tion	t [h]	conv. [%]	Aspect	$M_{n,theo}$ [kDa]	M_n [Da]	M_w [Da]	PDI
**1.**	neat	10	hν	20	82	elastic rubber	9 900	21 528	56 934	2.64
**2.**		150		1	99		726	173 961	302 767	1.74
**3.**		150	80 °C	72	99		726	127 272	974 892	7.66
**4.**	DCM	150	hν	2	37	-	-	-	-	-
**5.**		150		20	37	-	271	6445	14578	2.26
**6.**	toluene	150		12	99	Foam	726	188439	188.4	2.21

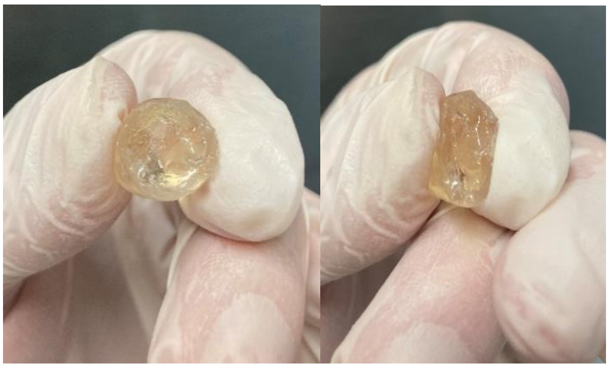

Figure S17. Pictures of the **polyCOE** obtained from the reaction of **COE** (1.0 equiv.) with 10 ppm of **Ru8** in neat with light activation.

GPC Summary

Chromatogram Detector 1 Ch1

#	Title	Mn	Mw	Mz	Mz1	Mw/Mn
1	2502_PCO_4.lcd	173961	302767	568940	996511	1,74043
	Average	173961	302767	568940	996511	1,74043

GPC Graph

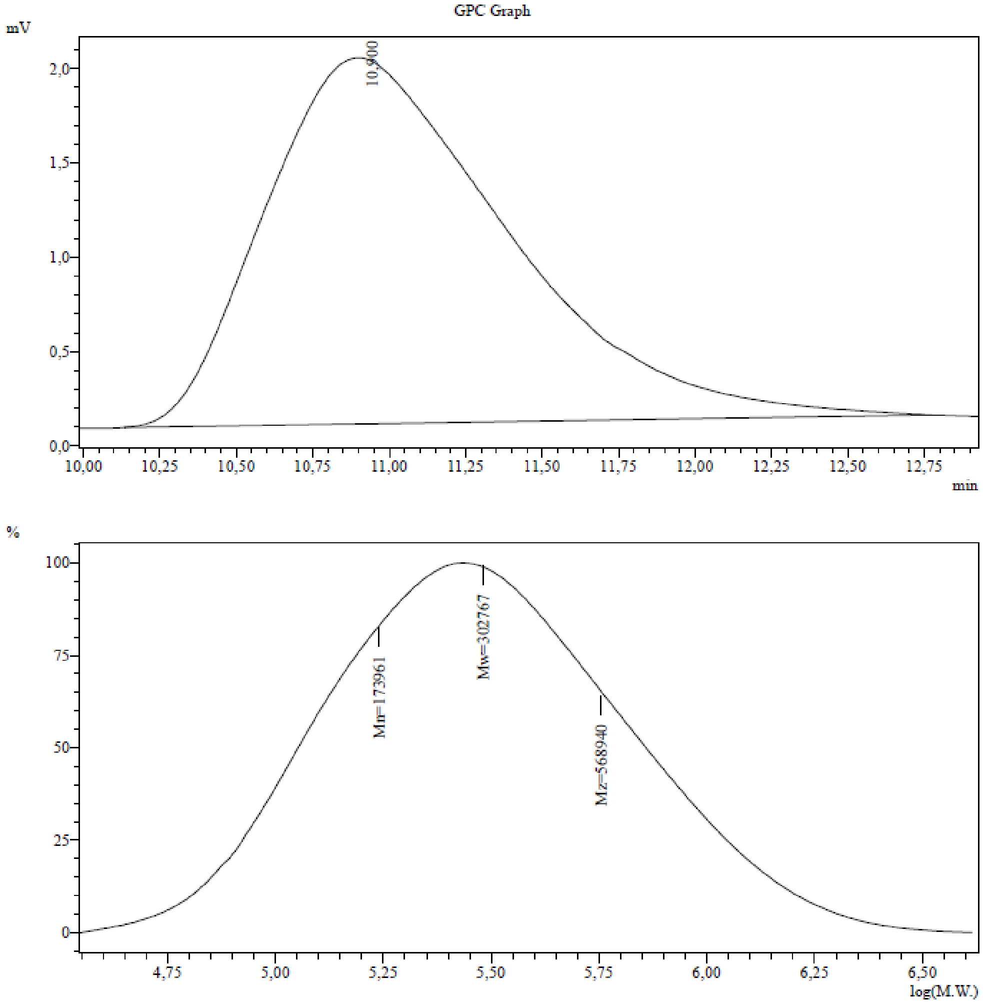

Figure S18. GPC traces of the **polyCOE** obtained from the reaction of **COE** (1.0 equiv.) with 150 ppm of **Ru8** in neat with light activation.

GPC Graph

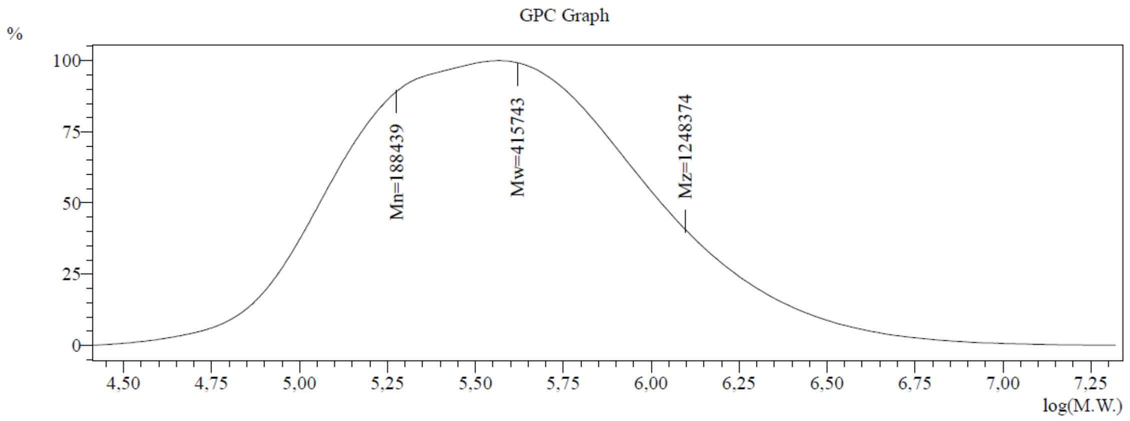

GPC Summary

Chromatogram Detector 1 Ch1

Title	Mn	Mw	Mz	Mw/Mn
1-1208.lcd	188439	415743	1248374	2.20624

Figure S19. GPC traces of the **polyCOE** obtained from the reaction of **COE** (1.0 equiv.) with 150 ppm of **Ru8** in toluene with light activation.

GPC Graph

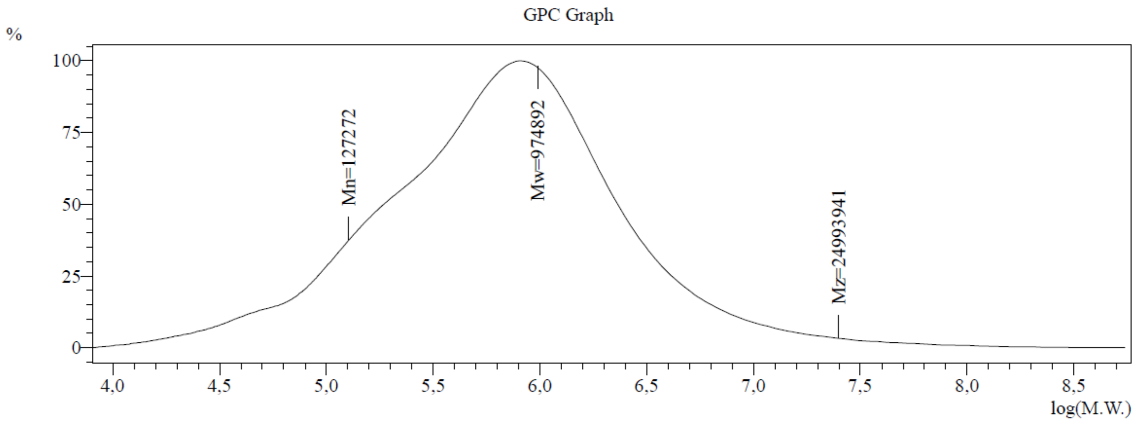

GPC Summary

Chromatogram Detector 1 Ch1

Title	Mn	Mw	Mz	Mw/Mn
0103_COE_heat.lcd	127272	974892	24993941	7.65994

Figure S20. GPC traces of the **polyCOE** obtained from the reaction of **COE** (1.0 equiv.) with 150 ppm of **Ru8** in neat with heat activation.

GPC Graph

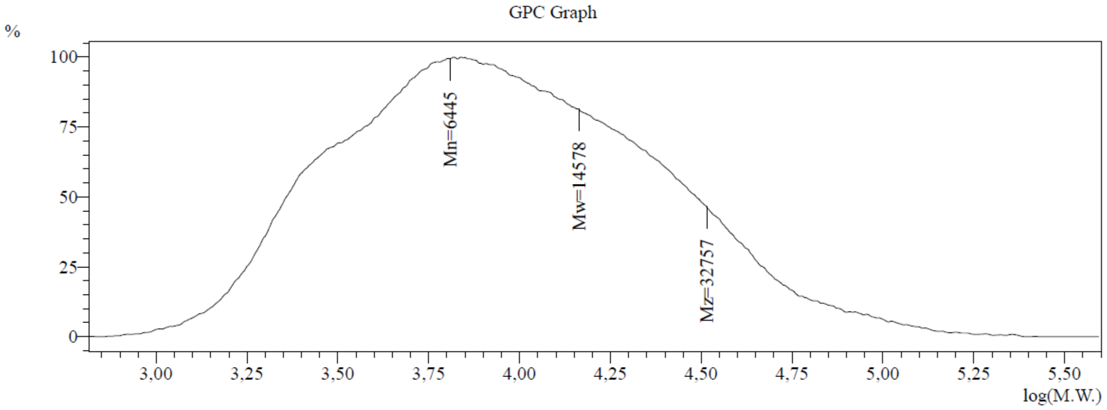

GPC Summary

Chromatogram Detector 1 Ch1

Title	Mn	Mw	Mz	Mw/Mn
0303 COE hv DCM.lcd	6445	14578	32757	2.26179

Figure S21. GPC traces of the **polyCOE** obtained from the reaction of **COE** (1.0 equiv.) with 150 ppm of **Ru8** in DCM with light activation.

GPC Graph

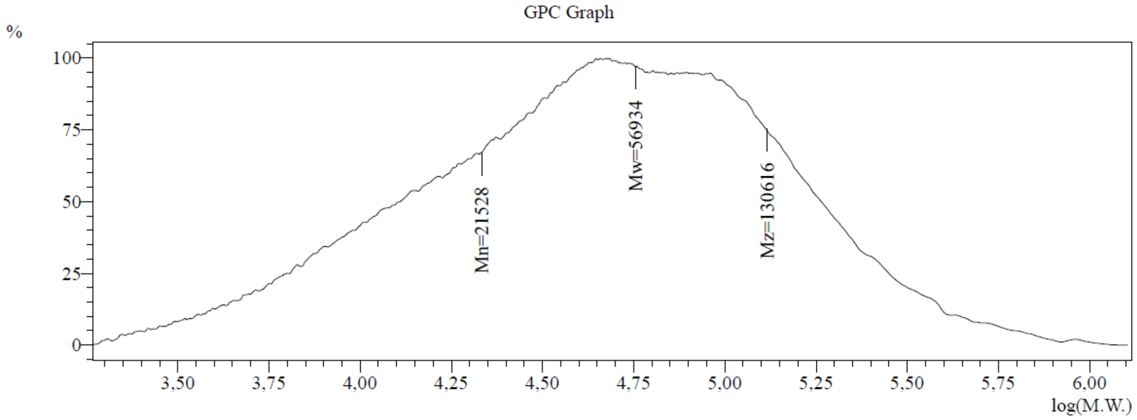

GPC Summary

Chromatogram Detector 1 Ch1

Title	Mn	Mw	Mz	Mw/Mn
0303 COE 10ppm.lcd	21528	56934	130616	2.64459

Figure S22. GPC traces of the **polyCOE** obtained from the reaction of **COE** (1.0 equiv.) with 10 ppm of **Ru8** in neat with light activation.

Polymerization of Dicyclopentadiene (DCPD)

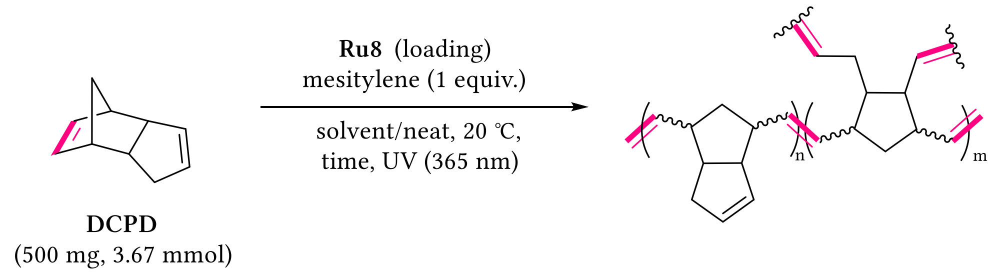

Scheme S13. ROMP polymerization of **DCPD** promoted by **Ru8** catalyst under UV irradiation.

15 mL vial equipped with a screw cap was charged with **DCPD** (500 mg, 3.67 mmol, 1 equiv.) and mesitylene (441 mg, 508 µL, 3.67 mmol, 1 equiv.) as internal standard. To the resulting mixture was added **Ru8** catalyst stock solution (1 M, 0.38 mL, 0.378 mg, 0.55 µmol, 150 ppm, 0.0150 mol%). The solvent (DCM) was removed under the reduced pressure before photochemical activation. The vial was then placed in the UV-reactor (365 nm) and was left for a required time affording a transparent solid polymer (hard and odorless). The table containing the results of conducted ROMP reactions is presented below.

In case of reaction with 10 ppm of **Ru8**, the following conditions has been used:
To a 15 mL screw capped vial containing **DCPD** (1200 mg, 8.8 mmol, 1 equiv.), mesitylene (1058 mg, 1220 µL, 8.8 mmol, 1 equiv.) was added as internal standard followed by the addition of **Ru8** catalyst stock solution in DCM (1 mg/mL, 0.06 mL, 0.06 mg, 0.08 µmol, 10 ppm, 0.0010 mol%).

Table S6. Results of ROMP reactions involving dicyclopentadiene as a substrate.

Entry	Solvent	Ru [ppm]	t [h]	conv. [%]	Aspect
**1.**	neat	10	6	71	transparent soft rubber
**2.**		10	12	84	transparent soft rubber
**3.**		150	1	99	transparent hard rubber

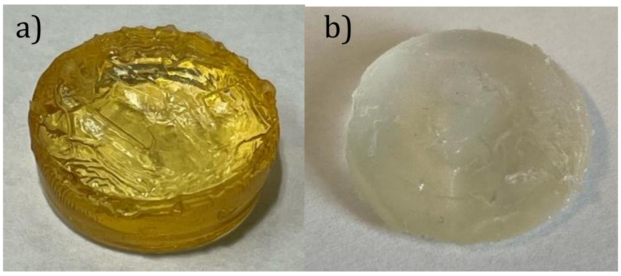

Figure S23. Pictures of **polyDCPD** obtained from the reaction of **DCPD** (1.0 equiv.) in neat with light activation with a) 150 ppm of **Ru8** b) 10 ppm of **Ru8**.

Polymerization of Norbornene

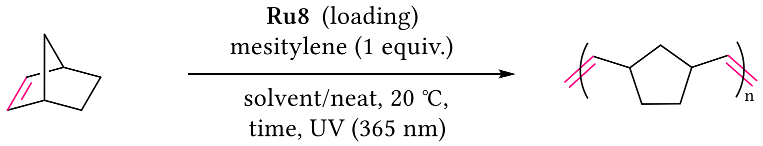

6
(360 mg, 2.93 mmol)

7

Scheme S14. ROMP polymerization of norbornene promoted by **Ru8** catalyst under UV irradiation.

15 mL vial equipped with a screw cap was charged with substrate **6** (276 mg, 2.93 mmol, 1 equiv.). Unless the reaction was carried out in neat, an appropriate solvent (6 mL) was added (DCM or toluene). To the resulting solution mesitylene (353 mg, 407 µL, 2.93 mmol, 1 equiv.) was added as internal standard followed by the addition of **Ru8** catalyst stock solution in DCM (1 M, 0.3 mL, 0.303 mg, 0.44 µmol, 150 ppm, 0.0150 mol%). For the reactions carried out in neat conditions, the solvent (DCM) was removed under the reduced pressure before photochemical activation. The vial was then placed in the UV-reactor (365 nm) or in heated oil bath (80 °C) and was left for a required time affording a highly viscous solution. NMR of this crude solution was recorded. The resulting polymer was precipitated out with acetone and methanol then filtered off. In the case of reaction in neat, the solid was washed with hexane and DCM and filtered. The filtrate was analyzed by ^{1}H NMR to assess the conversion of the reaction.

In case of reaction with 10 ppm of **Ru8**, the following conditions has been used:
To a 15 mL screw capped vial containing **6** (1026 mg, 10.9 mmol, 1 equiv.), mesitylene (1310 mg, 1510 µL, 10.9 mmol, 1 equiv.) was added as internal standard followed by the addition of **Ru8** catalyst stock solution in DCM (1 mg/mL, 0.075 mL, 0.075 mg, 0.11 µmol, 10 ppm, 0.0010 mol%).

Table S7. Results of ROMP reactions involving norbornene as a substrate.

Entry	Solvent	Ru [ppm]	t [h]	conv. [%]	Aspect	$M_{n,theo}$ [Da]	M_n [Da]	M_w [Da]	PDI
**1.**	neat	10	12	99	colorless rubber	9 325 800	318 624	650 241	2.04
**2.**		150	1	99		621 720	-	-	-
**3.**	DCM	150	20	91	grey solid	571 480	25 410	65 697	2.59

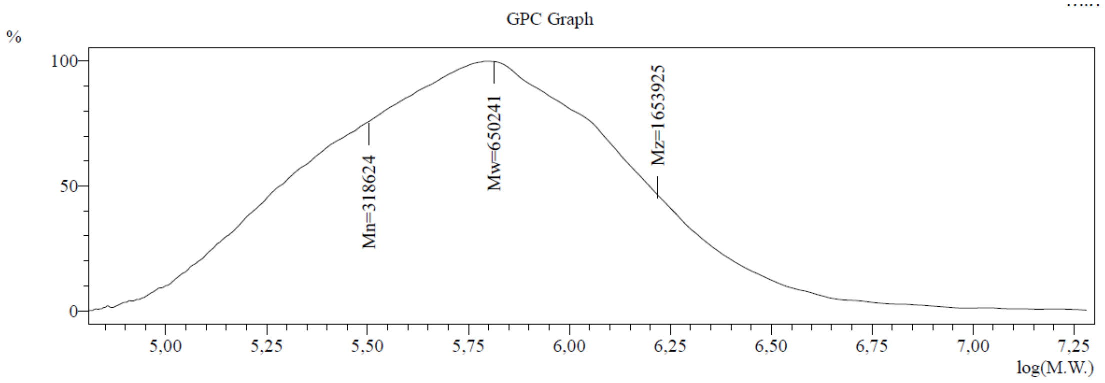

GPC Summary

Chromatogram Detector 1 Ch1

#	Mn	Mw	Mz	Mw/Mn
1	318624	650241	1653925	2,04078

Figure S24. GPC traces of the polynorbornene obtained from the reaction of **6** (1.0 equiv.) with 10 ppm of **Ru8** in neat with light activation.

GPC Graph

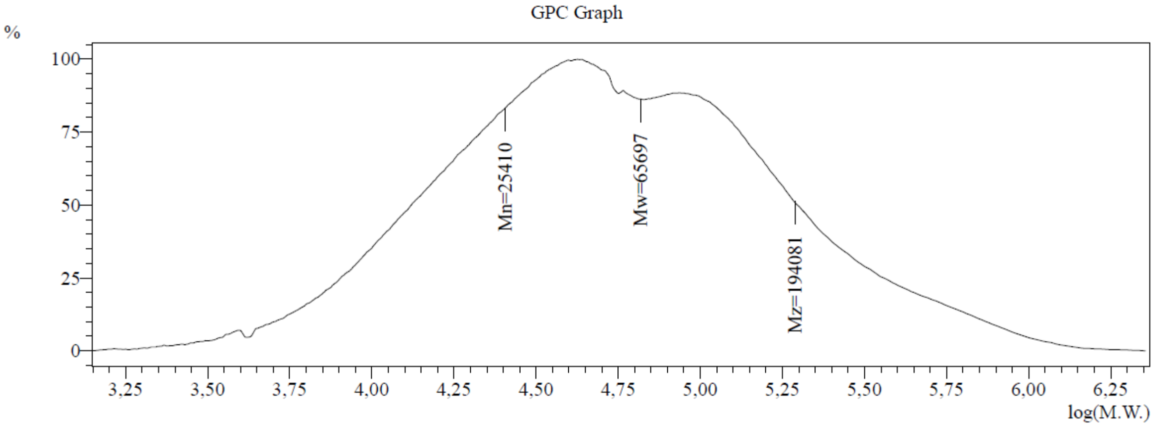

GPC Summary

Chromatogram Detector 1 Ch1

Title	Mn	Mw	Mz	Mw/Mn
0303_norn_hv_150ppm_DCM.lcd	25410	65697	194081	2,58550

Figure S25. GPC traces of the polynorbornene obtained from the reaction of **6** (1.0 equiv.) with 10 ppm of **Ru8** in DCM with light activation.

Polymerization of Cycloocta-1,5-diene (COD)

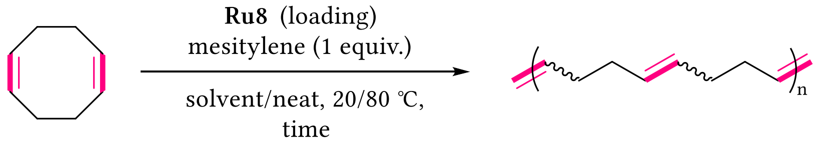

COD
(324 mg, 2.93 mmol)

polyCOD

Scheme S15. ROMP polymerization of cyclooctadiene promoted by **Ru8** catalyst.

15 mL vial equipped with a screw cap was charged with substrate **COD** (324 mg, 367 µL, 2.93 mmol, 1 equiv.). Unless the reaction was carried out in neat, an appropriate solvent (6 mL) was added (DCM or toluene). To the resulting solution mesitylene (353 mg, 407 µL, 2.93 mmol, 1 equiv.) was added as internal standard followed by the addition of **Ru8** catalyst stock solution in DCM (1 M, 0.3 mL, 0.303 mg, 0.44 µmol, 150 ppm, 0.0150 mol%). For the reactions carried out in neat conditions, the solvent (DCM) was removed under the reduced pressure before photochemical activation. The vial was then placed in the UV-reactor (365 nm) or in heated oil bath (80 °C) and was left for a required time affording a highly viscous solution. NMR of this crude solution was recorded and highlighted the presence of the polymer **polyCOD**, which was subsequently precipitated out with acetone and then filtered off. Despite the flexibility of the obtained material, it can be broken easily on elongation. The filtrate was analyzed by NMR spectroscopy to determine the conversion of the substrate. The table containing the results of conducted ROMP reactions is presented below.

Table S8. Results of ROMP reactions involving **COD** as a substrate. N.D. = not detected.

Entry	Solvent	Ru [ppm]	Activation	t [h]	conv. [%]	Aspect	$M_{n,theoretical}$ [Da]	M_n [Da]	M_w [Da]	PDI
**1.**	neat	150	hν	6	68	elastic rubber	-	N.D	N.D	N.D
**2.**		150	80 °C	72	0	-	-	-	-	-
**3.**	DCM	150	hν	20	0	-	-	-	-	-

Self Cross-Metathesis of Methyl Oleate (MO)

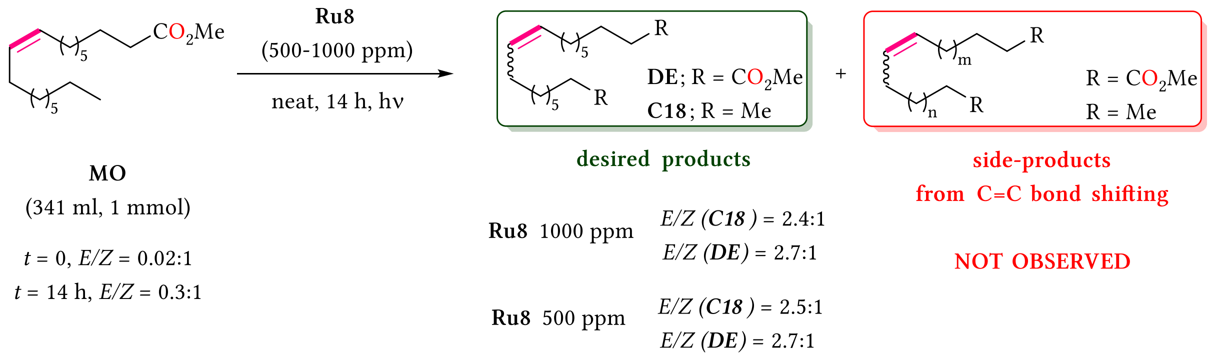

Scheme S16. Self-CM of **MO** promoted by **Ru8** catalyst.

Methyl oleate (**MO**) (341 µL, 1 mmol) was introduced into a 1.5 mL screw-capped vial. The air in the flask was replaced by argon. The catalyst **Ru8** (0.69 mg, 0.001 equiv., 1 µmol, 0.1 mol% or 0.35 mg, 0.5 µmol, 0.05 mol%) was added as DCM solution (1 mg/mL). The solvent (DCM) was evaporated from the vessel under reduced pressure followed by refilling the vial with argon atmosphere before placing the vial in a UV-reactor. Then, the reaction mixture was irradiated for 14 h. After this time, a solution of SnatchCat was added and the reaction mixture was diluted in toluene. The resulting solution was injected to the GC-MS.

Calculation of % of isomerization:[8]

$$\%iso\ (\boldsymbol{C18}) = \frac{\ldots + \%(\mathbf{C18}-1) + \%(\mathbf{C18}+1) + \cdots}{\ldots + \%(\mathbf{C18}-1) + \%(\mathbf{C18}) + \%(\mathbf{C18}+1) + \cdots} \times 100$$

$$\%iso\ (\boldsymbol{MO}) = \frac{\ldots + \%(\mathbf{MO}-1) + \%(\mathbf{MO}+1) + \cdots}{\ldots + \%(\mathbf{MO}-1) + \%(\mathbf{MO}) + \%(\mathbf{MO}+1) + \cdots} \times 100$$

$$\%iso\ (\boldsymbol{DE}) = \frac{\ldots + \%(\boldsymbol{DE}-1) + \%(\boldsymbol{DE}+1) + \cdots}{\ldots + \%(\boldsymbol{DE}-1) + \%(\boldsymbol{DE}) + \%(\boldsymbol{DE}+1) + \cdots} \times 100$$

$$Global\ \%iso = \frac{\%iso\ (\boldsymbol{C18}) + \%iso\ (\boldsymbol{MO}) + \%iso\ (\boldsymbol{DE})}{3}$$

[8] D. Butilkov, A. Frenklah, I. Rozenberg, S. Kozuch, N. G. Lemcoff, *ACS Catal*. **2017**, *7*, 7634.

Calculation of selectivity toward C=C bond shifting:

$$Selectivity\ (\boldsymbol{C18}) = \frac{\%(\mathbf{C18})}{\dots + \%(\mathbf{C18}-1) + \%(\mathbf{C18}) + \%(\mathbf{C18}+1) + \cdots} \times 100$$

$$Selectivity\ (\boldsymbol{MO}) = \frac{\%(\mathbf{MO})}{\dots + \%(\mathbf{MO}-1) + \%(\mathbf{MO}) + \%(\mathbf{MO}+1) + \cdots} \times 100$$

$$Selectivity\ \ (\boldsymbol{DE}) = \frac{\%(\boldsymbol{DE})}{\dots + \%(\boldsymbol{DE}-1) + \%(\boldsymbol{DE}) + \%(\boldsymbol{DE}+1) + \cdots} \times 100$$

$$Global\ Selectivity\ = \frac{Selectivity\ (\boldsymbol{C18}) + Selectivity\ (\boldsymbol{MO}) + Selectivity\ (\boldsymbol{DE})}{3}$$

Table S9. GC-MS chromatogram analysis of self-CM of **MO** with **Ru8**.

Compound	R. T. (min)	Corr. area	% total
1000 ppm of **Ru8**			
Z-**C18**	9.986	24310.65	2.44
E-**C18**	10.029	57824.65	5.79
Z-Imp	11.128	11587.71	1.16
E-Imp	11.213	18193.05	1.82
Z-**MO**	12.666	598067.81	59.92
E-**MO**	12.693	158880.77	15.92
Z-**DE**	14.986	25075.30	2.51
E-**DE**	15.022	66965.84	6.71
500 ppm of **Ru8**			
Z-**C18**	9.984	29994.57	2.49
E-**C18**	10.028	74072.23	6.16
Z-Imp	11.124	12595.86	1.05
E-Imp	11.209	21613.80	1.80
Z-**MO**	12.667	716577.19	59.59
E-**MO**	12.691	196341.23	16.33
Z-**DE**	14.982	30115.75	2.50
E-**DE**	15.019	80346.05	6.68
1000 ppm dark control			
E-Imp	11.116	12193.41	1.86
Z-Imp	11.201	16013.88	2.45
E-**MO**	12.656	601909.25	92.01
Z-**MO**	12.801	12264.73	1.87

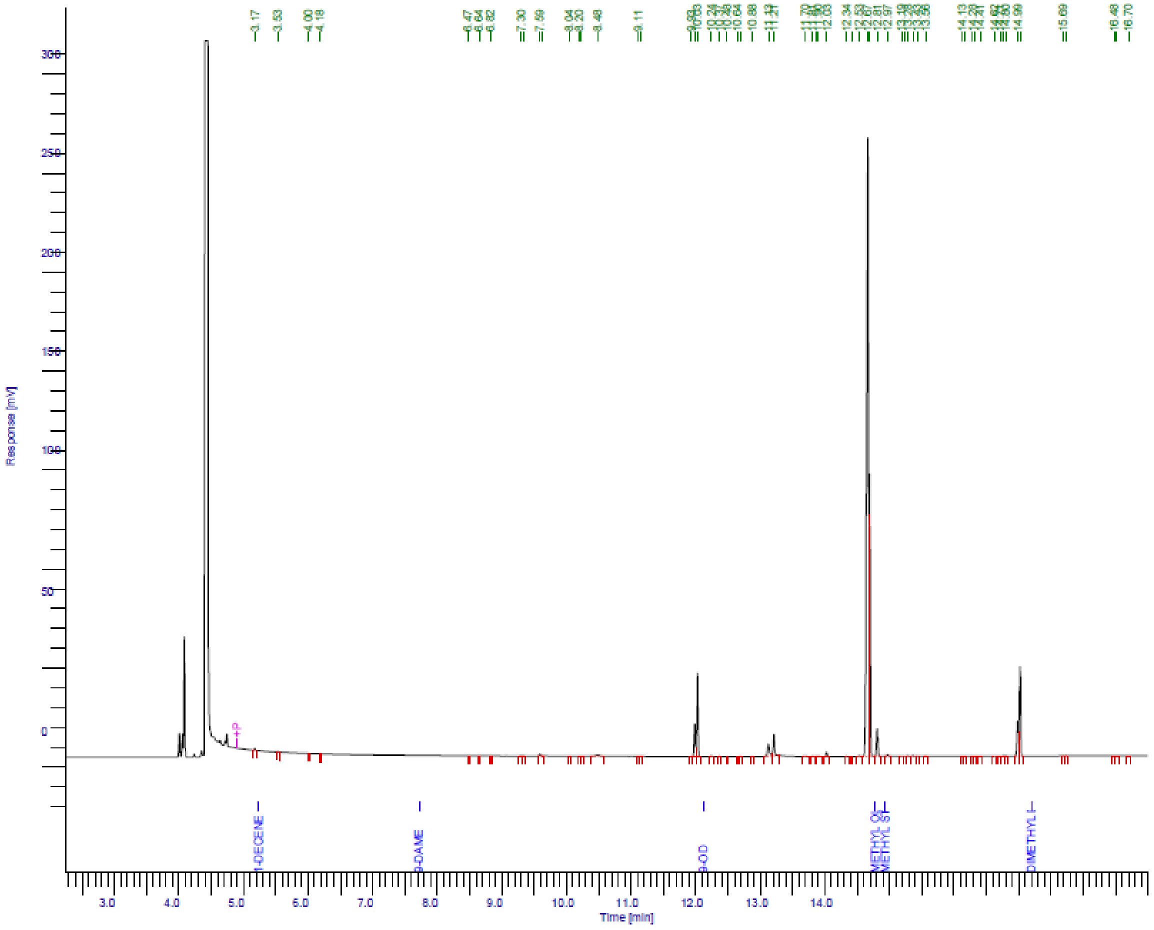

Figure S26. Chromatogram of self-CM of **MO** promoted by 1000 ppm of **Ru8** under light irradiation.

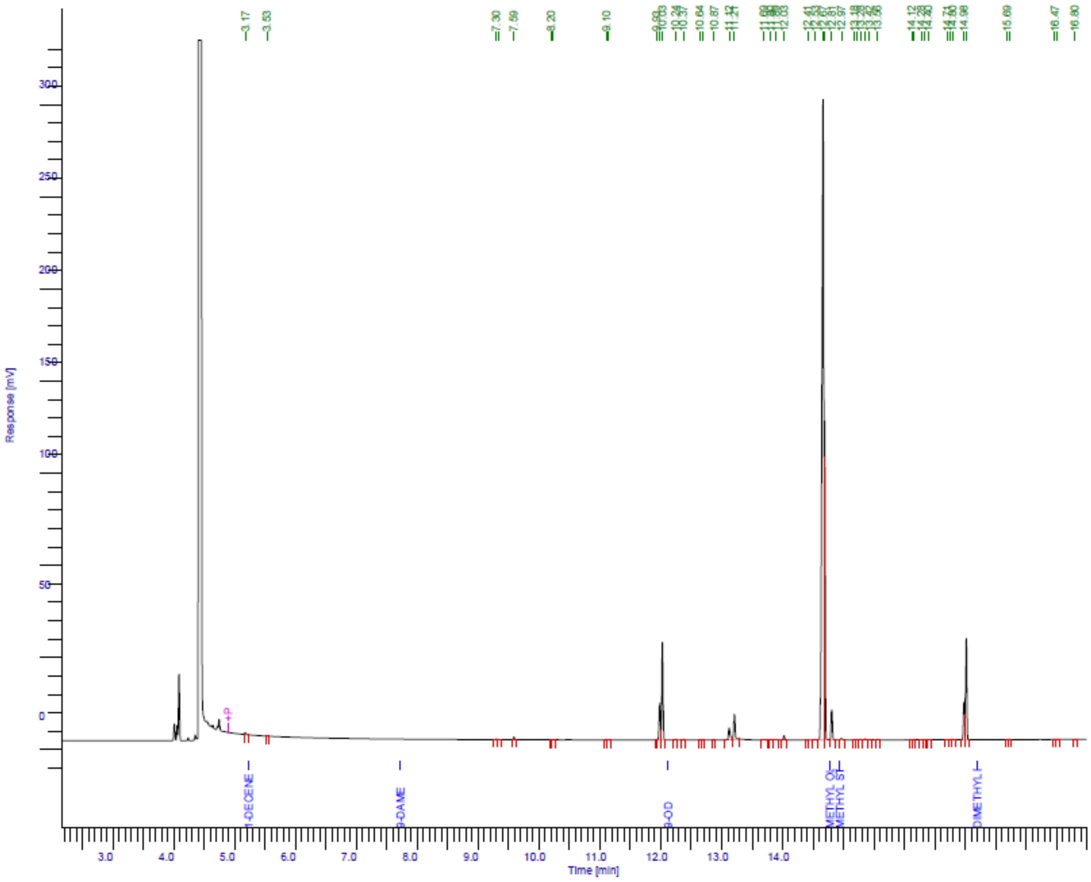

Figure S27. Chromatogram of self-CM of **MO** promoted by 500 ppm of **Ru8** under light irradiation.

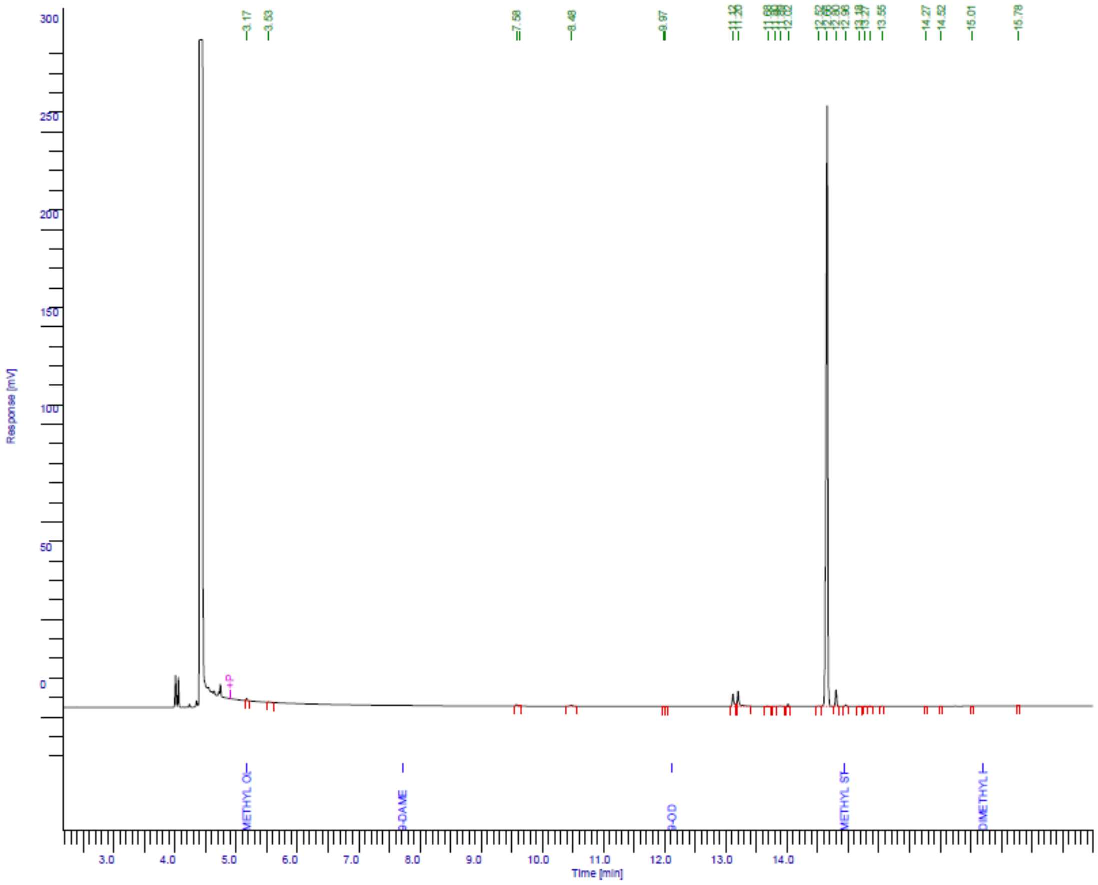

Figure S28. Chromatogram of self-CM of **MO** promoted by 500 ppm of **Ru8** in the dark.

NMR Spectra of New Compounds

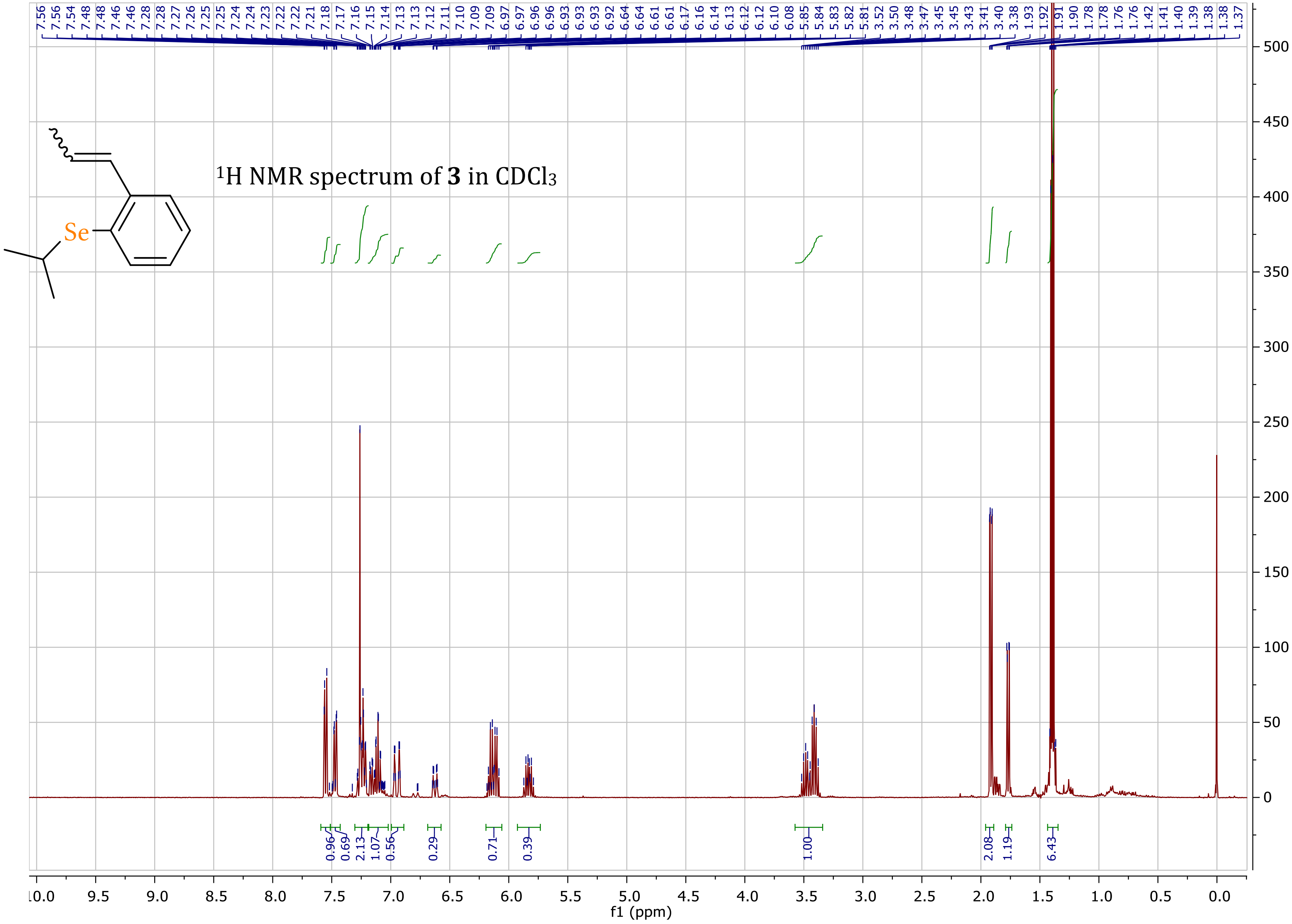

Figure S29. ^{1}H NMR spectrum of **3** in $CDCl_3$.

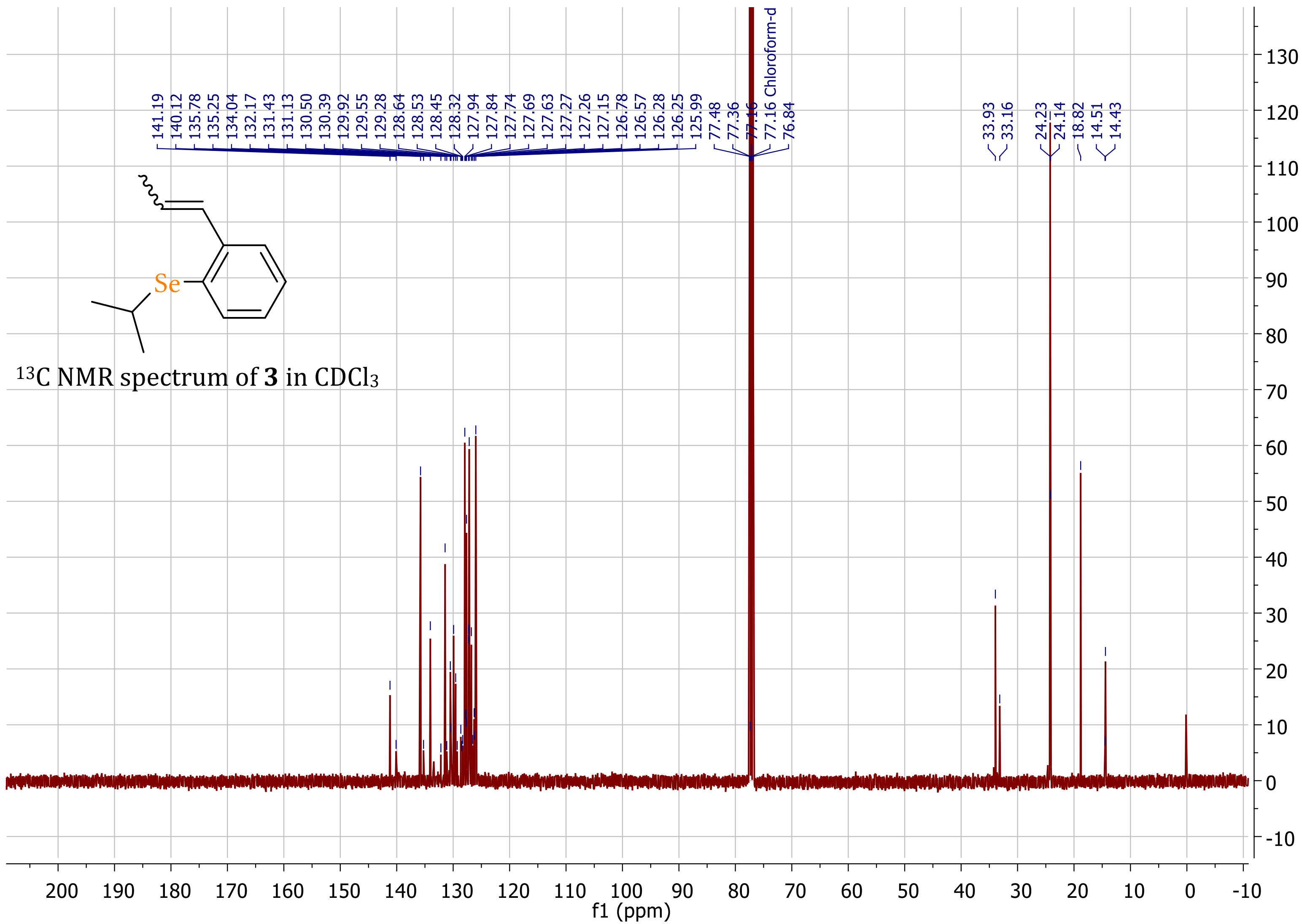

Figure S30. ^{13}C NMR spectrum of **3** in $CDCl_3$.

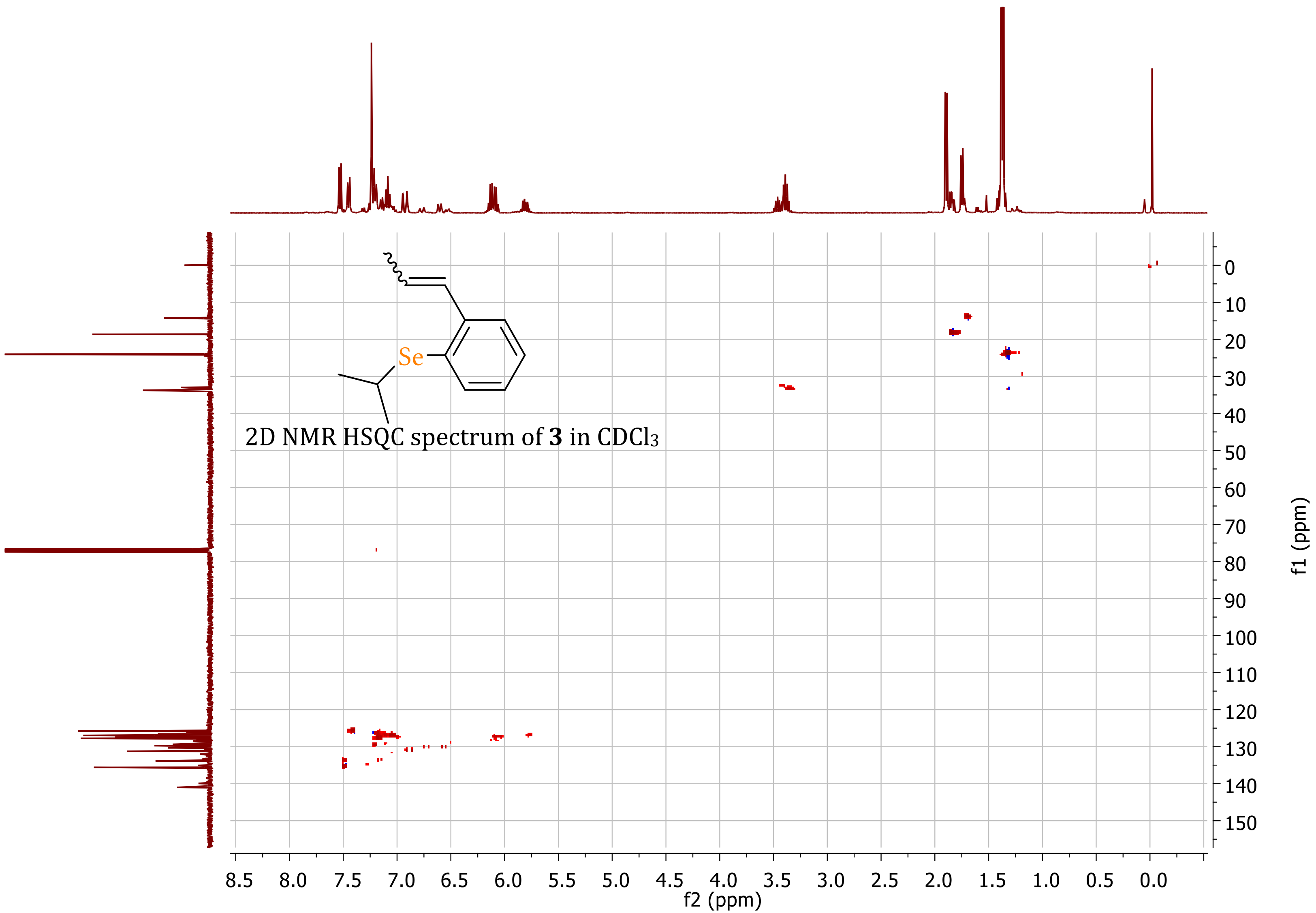

Figure S31. 2D NMR HSQC spectrum of **3** in $CDCl_3$.

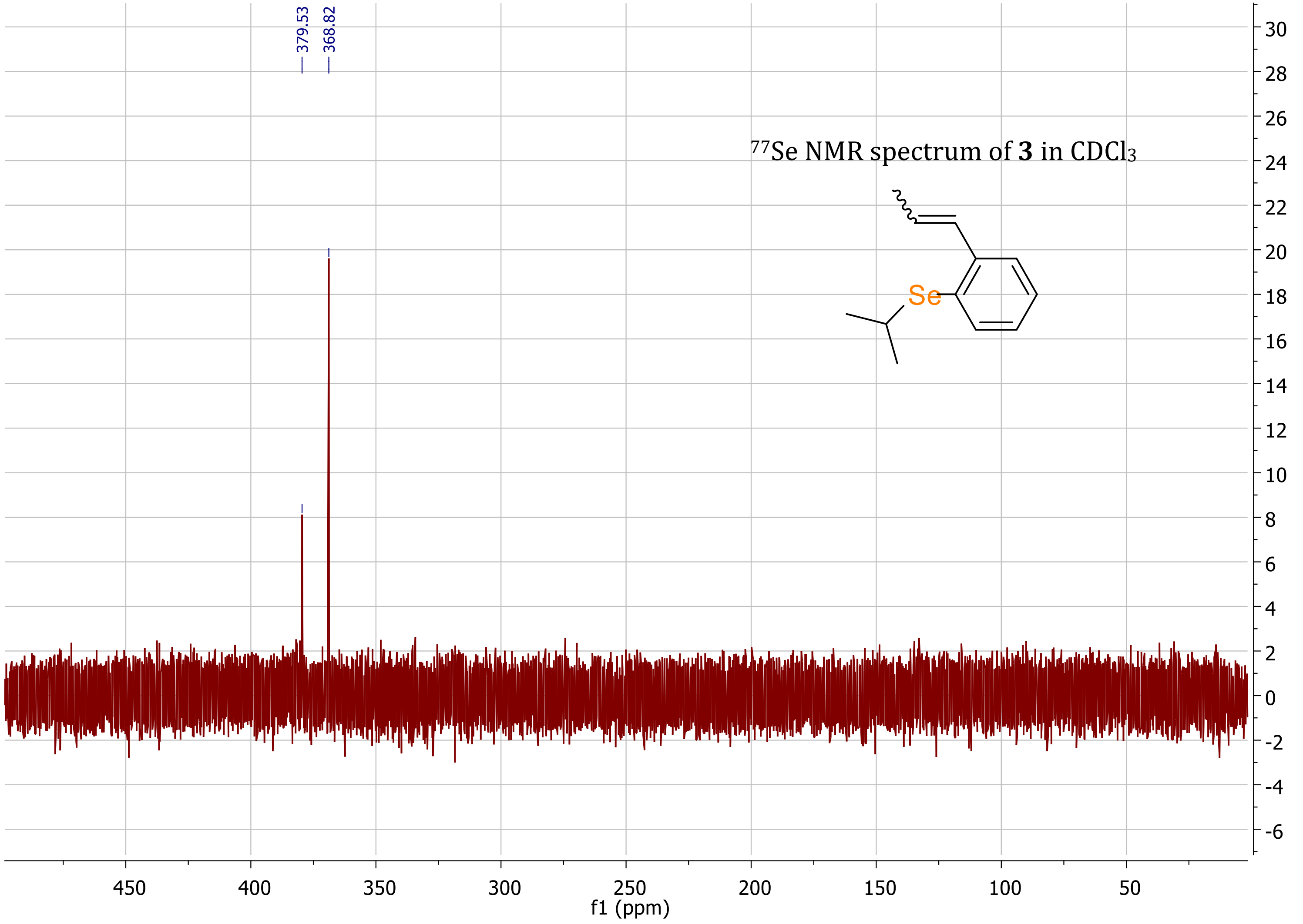

Figure S32. ^{77}Se NMR spectrum of **3** in $CDCl_3$.

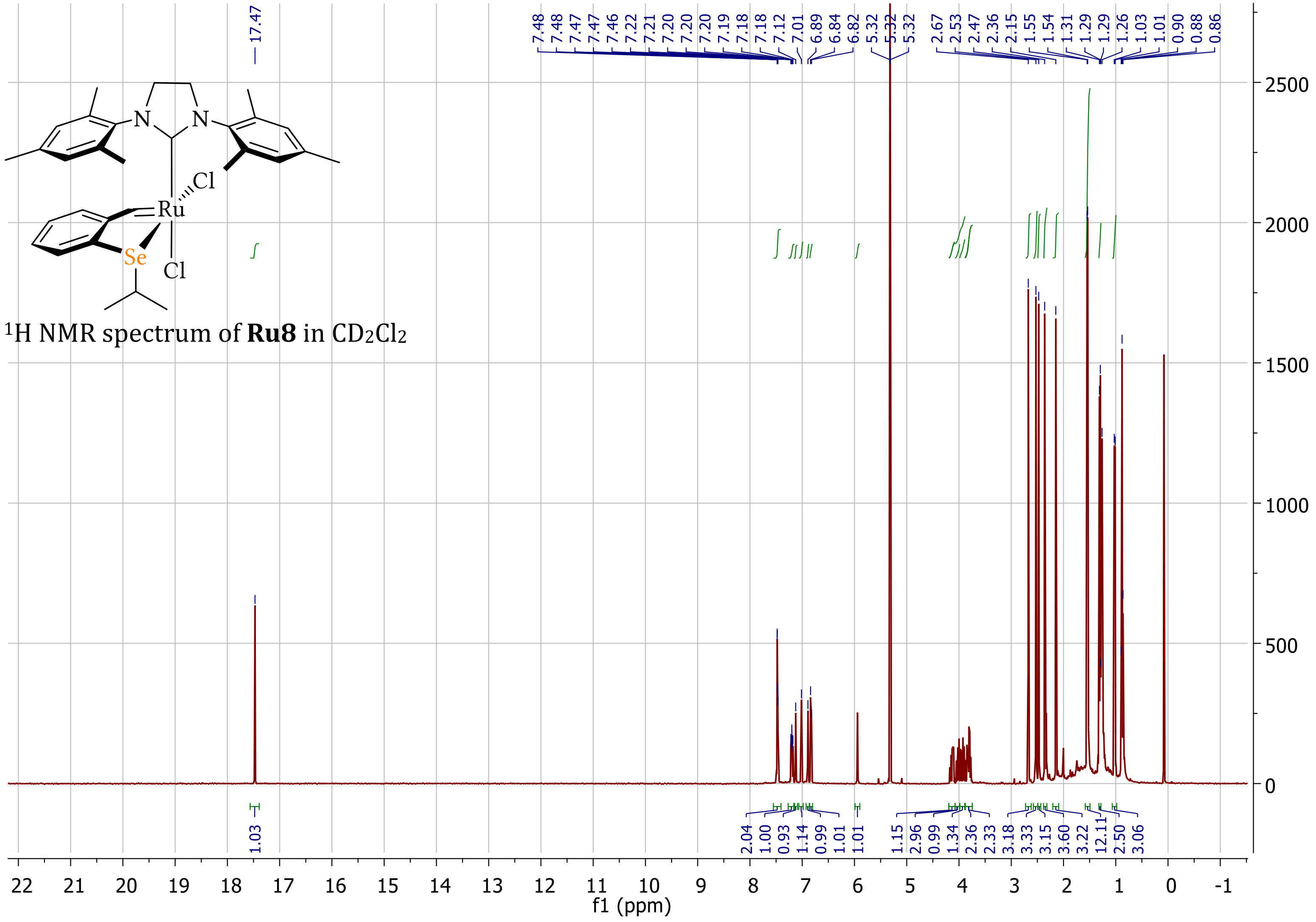

Figure S33. ^{1}H NMR spectrum of **Ru8** in CD_2Cl_2.

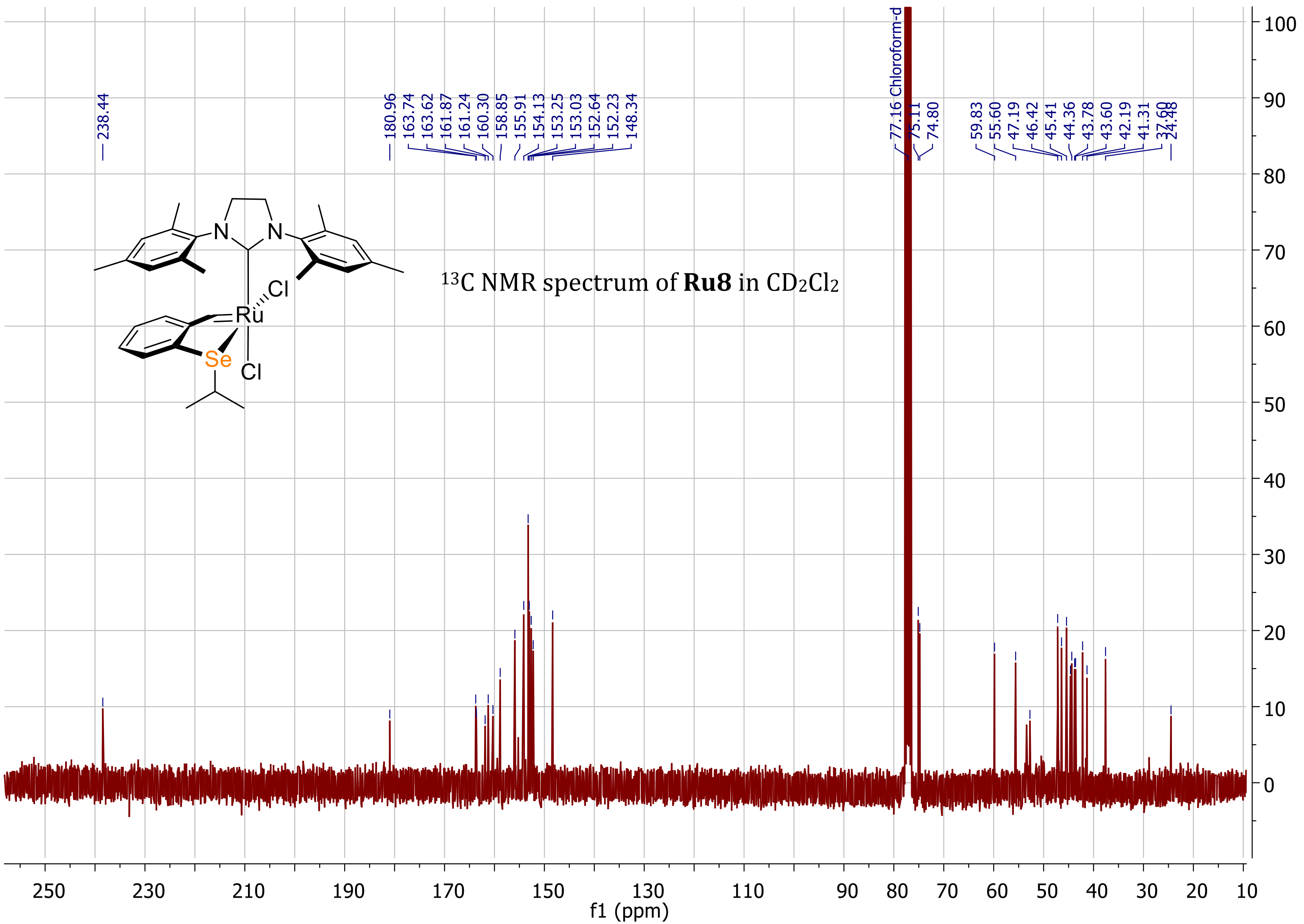

Figure S34. ^{13}C NMR spectrum of **Ru8** in CD_2Cl_2.

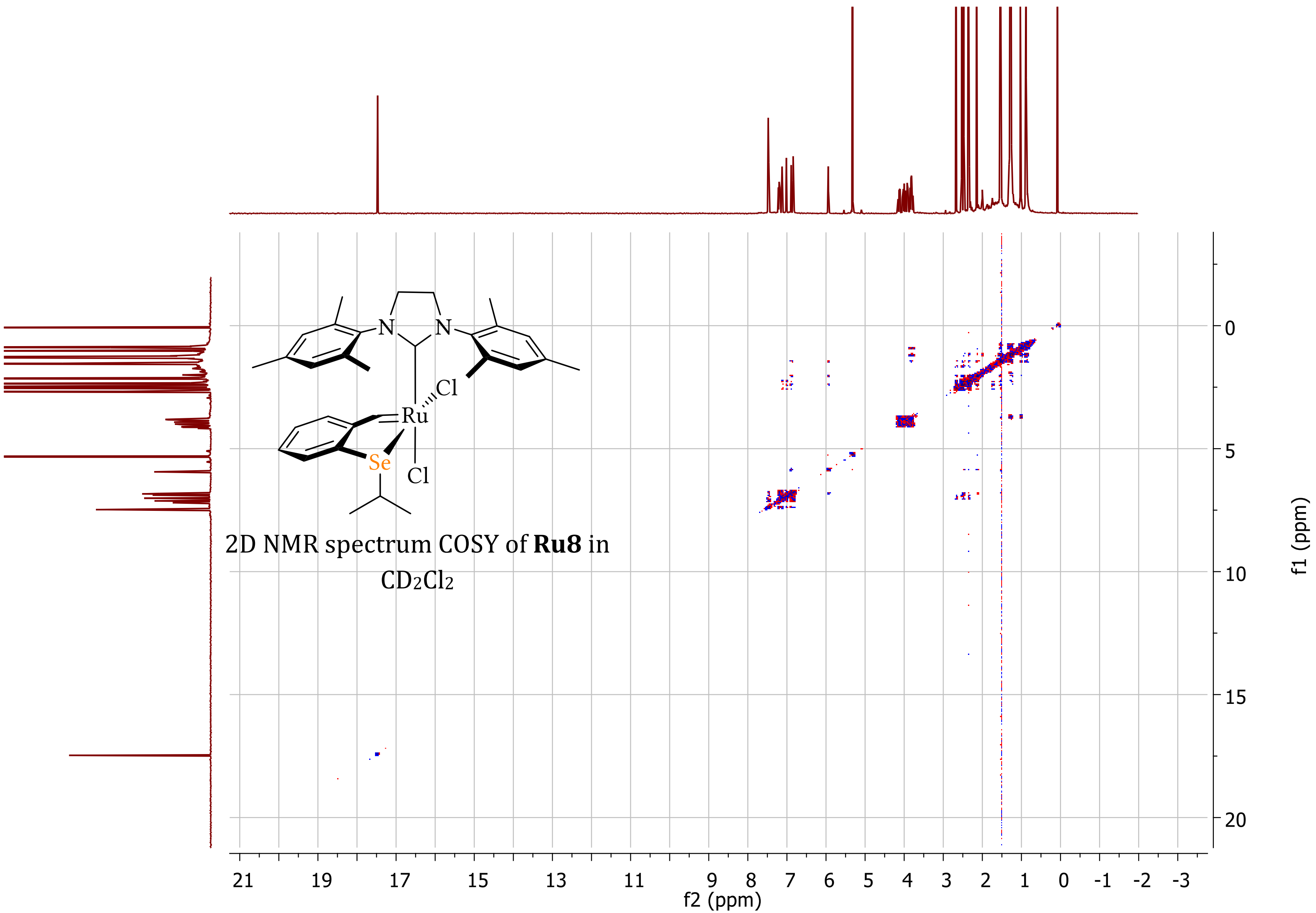

Figure S35. 2D NMR spectrum COSY of **Ru8** in CD_2Cl_2.

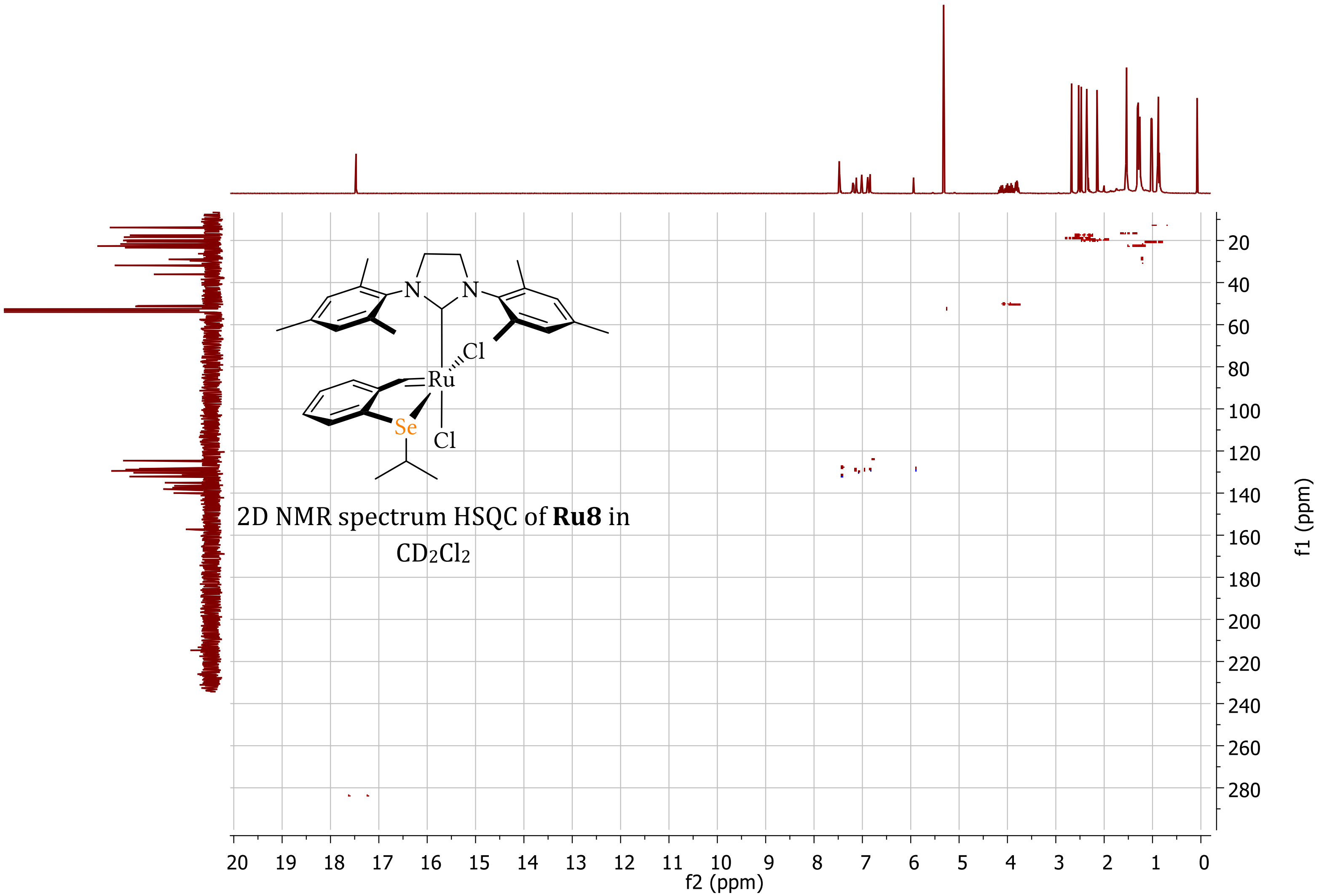

Figure S36. 2D NMR spectrum HSQC of **Ru8** in CD_2Cl_2.

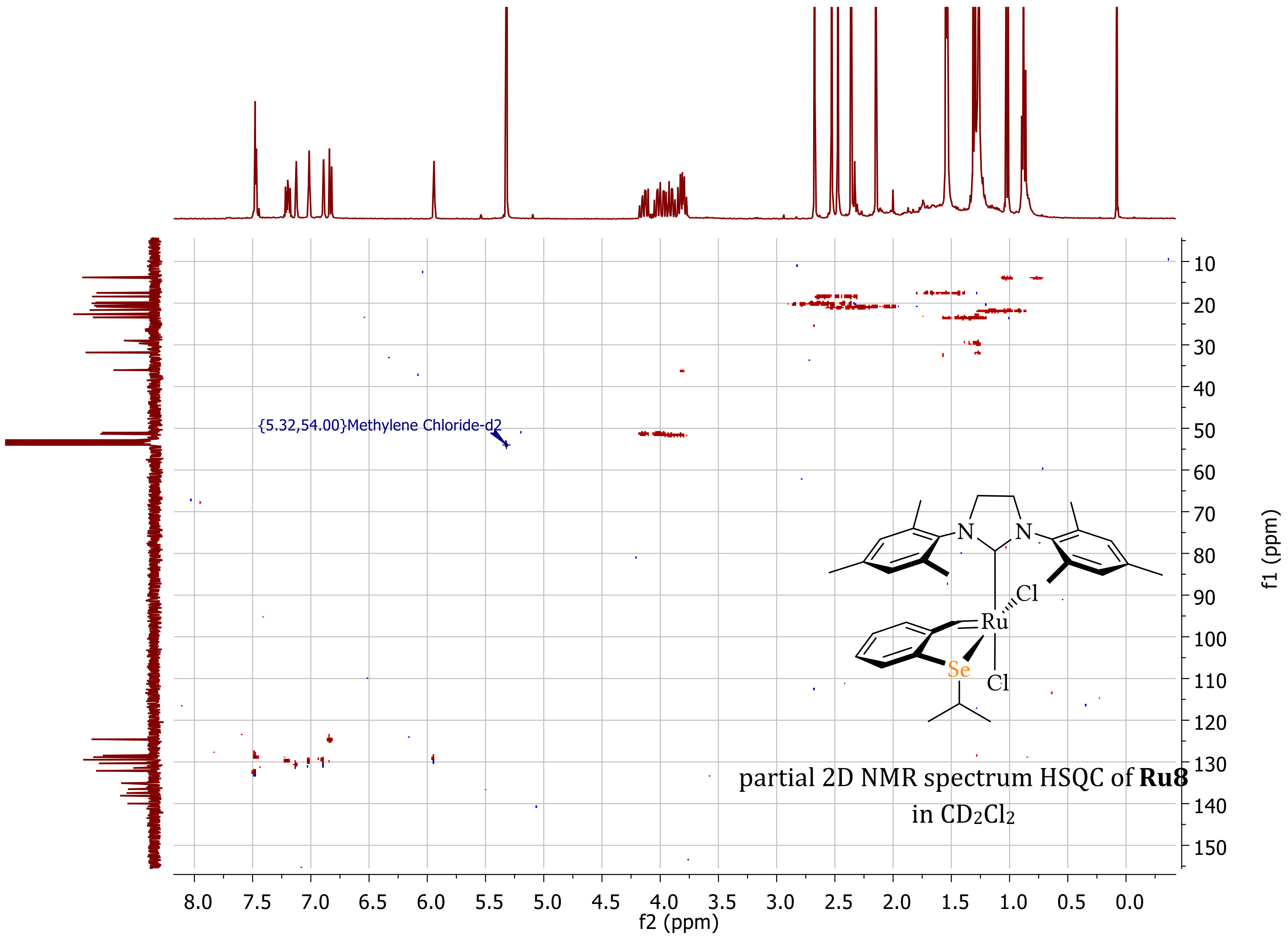

Figure S37. Partial 2D NMR spectrum HSQC of **Ru8** in CD_2Cl_2.

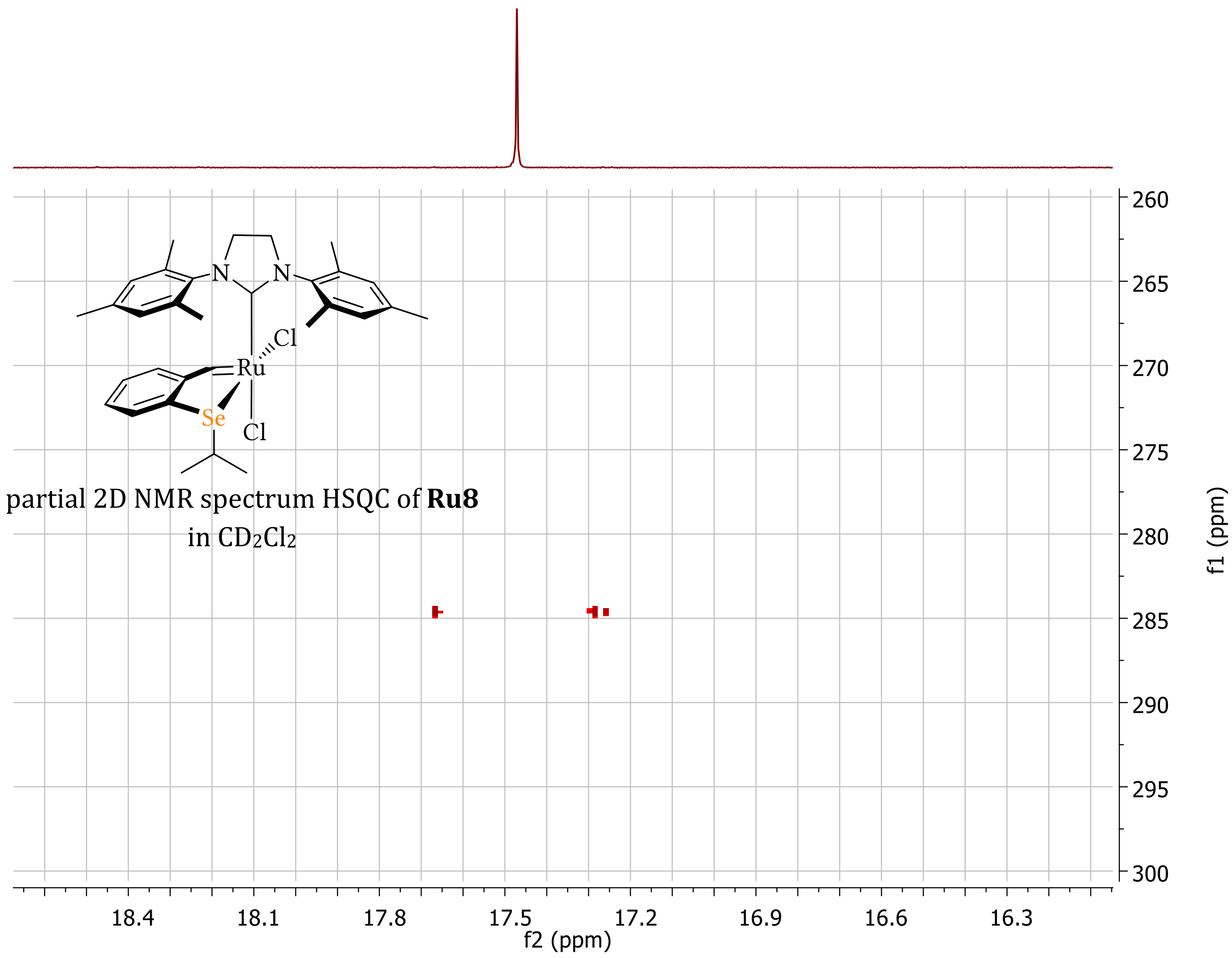

Figure S38. Partial 2D NMR spectrum HSQC of **Ru8** in CD_2Cl_2.

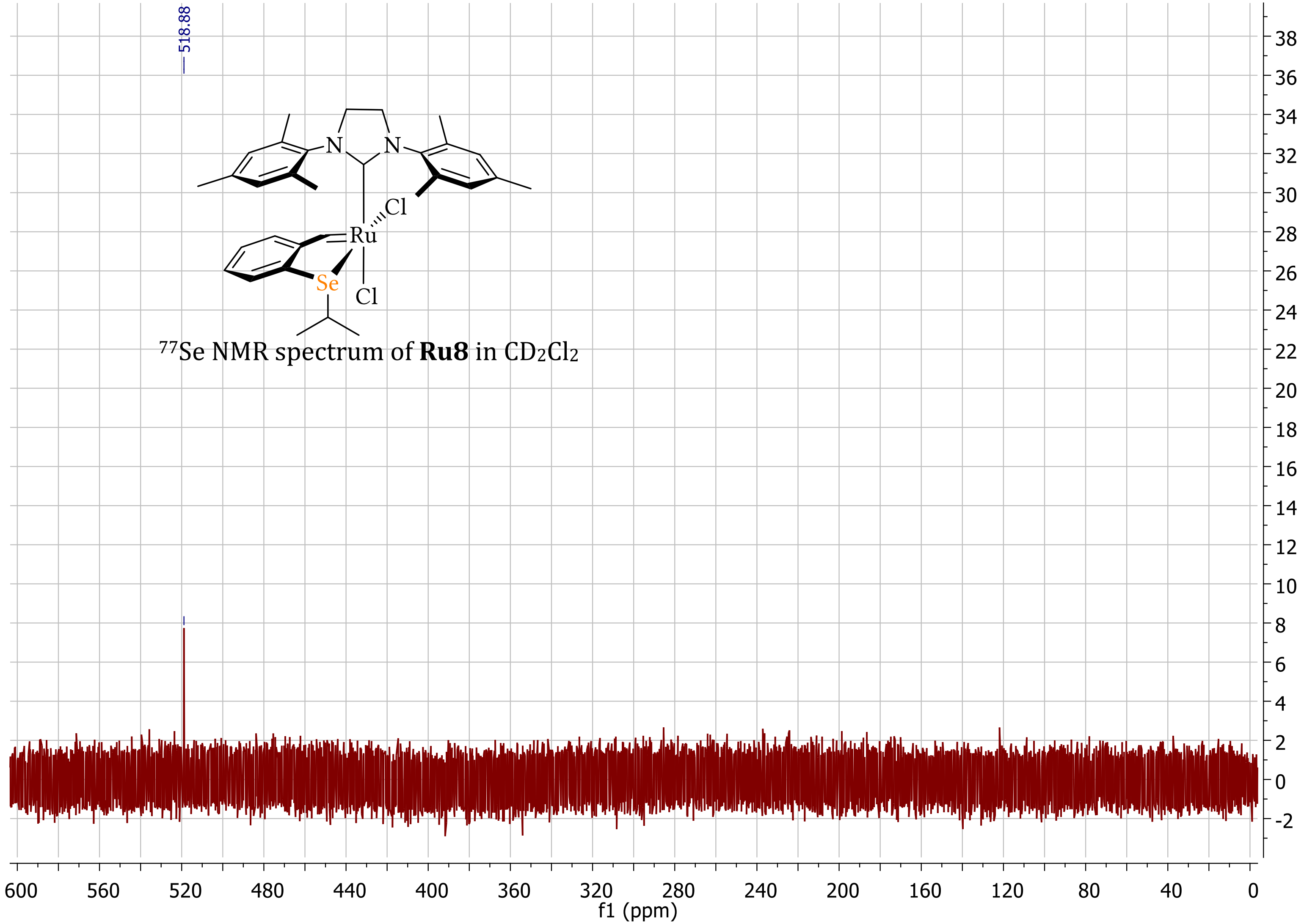

Figure S39. ^{77}Se NMR spectrum of **Ru8** in CD_2Cl_2.

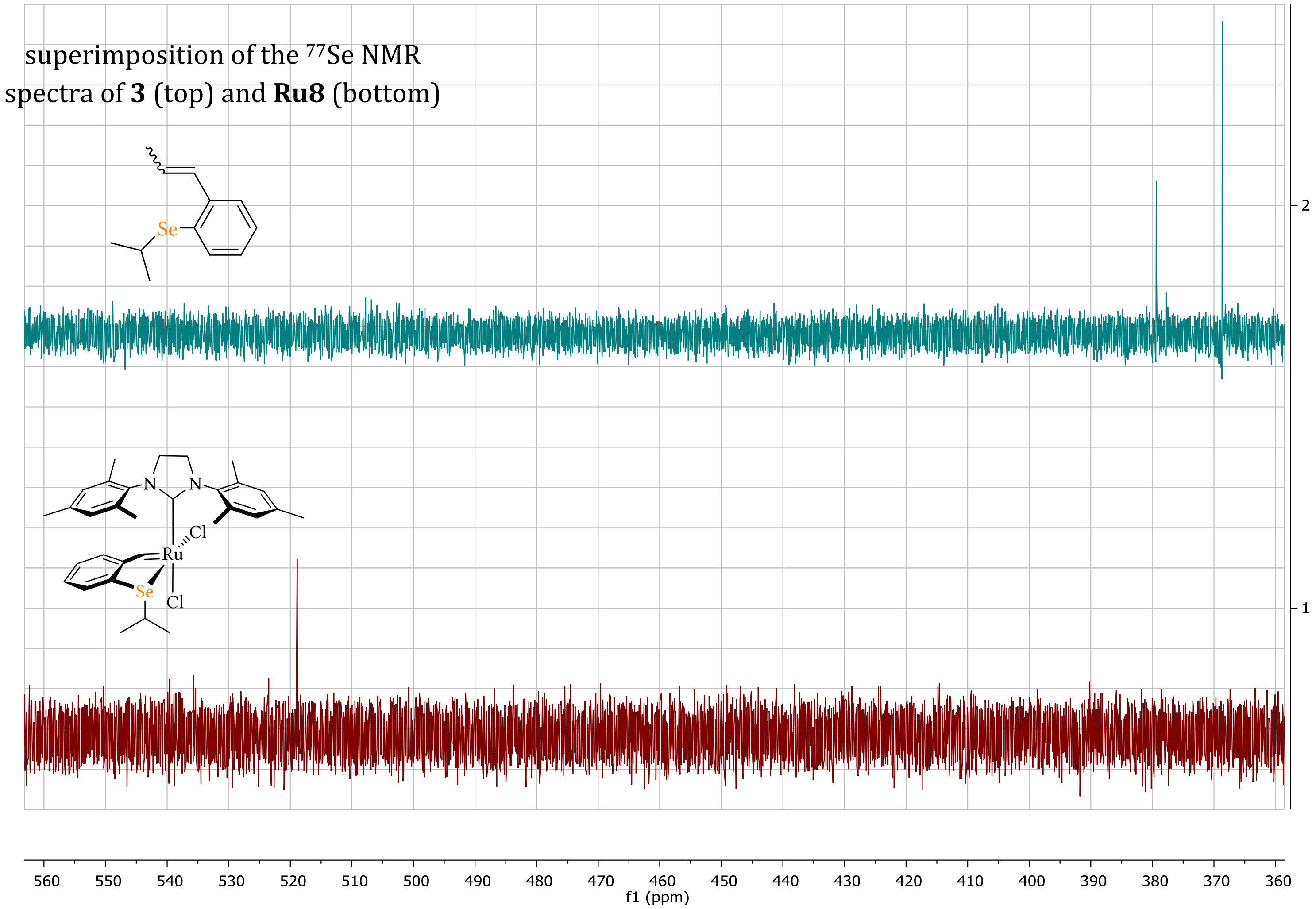

Figure S40. Superimposition of the ^{77}Se NMR spectra of **3** (top) and **Ru8** (bottom).
